# Ichnotaxonomic Review of Large Ornithopod Dinosaur Tracks: Temporal and Geographic Implications

**DOI:** 10.1371/journal.pone.0115477

**Published:** 2015-02-12

**Authors:** Ignacio Díaz-Martínez, Xabier Pereda-Suberbiola, Félix Pérez-Lorente, José Ignacio Canudo

**Affiliations:** 1 CONICET—Instituto de Investigación en Paleobiología y Geología, Universidad Nacional de Río Negro, General Roca 1242, 8332 Fisque Menuco (General Roca), Río Negro, Argentina; 2 Facultad de Ciencas, Estudios Agroalimentarios e Informática, Universidad de La Rioja, Madre de Dios 51–53, 26006 Logroño, La Rioja, Spain; 3 Universidad del País Vasco/Euskal Herriko Unibertsitatea, Facultad de Ciencia y Tecnología, Departamento de Estratigrafía y Paleontología, Apartado 644, 48080, Bilbao, Spain; 4 Grupo Aragosaurus-IUCA, Área de Paleontología, Facultad de Ciencias, Universidad de Zaragoza, Pedro Cerbuna 12, 50009, Zaragoza, Spain

## Abstract

**Background:**

Large ornithopod tracks are known from the Upper Jurassic to the uppermost Cretaceous rocks of all continents but Antarctica. They include the tracks historically called *Iguanodon* footprints, iguanodontid footprints, hadrosaur/hadrosaurid footprints, and other large ornithopod tracks that have been used to define ichnotaxa. More than 40 ichnospecies based on large ornithopod tracks have been defined, but the validity of many of them is questionable.

**Methodology/Principal Findings:**

34 ichnogenera and 44 ichnospecies have been analysed in this work. Many of them are considered to be invalid because they have been defined on the basis of poorly preserved tracks without diagnostic features, have an inadequate diagnosis, or are based on temporal and/or geographical criteria. Only eight ichnospecies belonging to the ichnogenera *Caririchnium*, *Iguanodontipus* and *Hadrosauropodus* are here regarded as valid.

**Conclusions/Significance:**

The monospecific ichnogenus *Iguanodontipus* (*I. burreyi*) is characterized by a small, rounded heel and elongate, narrow digit impressions. Its distribution is limited to the Berriasian-Valanginian of Europe. *Caririchnium* consists of four ichnospecies (*C. magnificum* [type ichnospecies], *C. kortmeyeri*, *C. billsarjeanti* and *C. lotus*) with a large, rounded heel and short, wide digit impressions. This ichnogenus ranges from the Berriasian-Hauterivian to the Aptian-Albian of South America, North America, Asia and Europe. Finally, *Hadrosauropodus* (three ichnospecies: *H. langstoni* [type ichnospecies], *H. leonardii* and *H. kyoungsookimi*) shows a large, bilobed heel and short, wide digit impressions. It is known from the Aptian-Albian to the Maastrichtian of North America, Asia and Europe. The ichnofamily Iguanodontipodidae includes large iguanodontian tracks characterized mainly by mesaxonic, tridactyl and subsymmetrical pes tracks that are as wide as (or wider than) long and have one pad impression in each digit and one in the heel. Its distribution is confidently limited to the Cretaceous of Europe, Asia, North America and South America.

## Introduction

Large ornithopod tracks have been studied since the beginnings of vertebrate ichnology in 19th-century Europe (e.g., [1–5]). Subsequently, large ornithopod tracks have been found in Asia (e.g., [6–7]), North America (e.g., [8–9]), South America (e.g., [10–11]), Australia [12] and Africa [13–14]. They include the tracks historically called *Iguanodon* footprints, iguanodont/iguanodontid footprints, hadrosaur/hadrosaurid footprints, as well as others that have been used to define ichnotaxa (e.g. *Amblydactylus*, *Iguanodontipus*, *Caririchnium*). The term “large ornithopod tracks” (or “large ornithopod footprints”) has been used in many ichnological papers (e.g., [15–18]). Thulborn [19] proposed that large ornithopod tracks are those larger than 25 cm and related them with iguanodonts and hadrosaurs. Nevertheless, this kind of track has not been formally defined. Recently, Moreno et al. [17] described them as follows: “Tridactyl, mesaxonic, with the lengths of digits II, III, and IV only slightly different; wide digits with rounded ends; digits converge proximally into a broad metatarsophalangeal impression (‘heel pad’). Ornithopod ichnites are similar in anteroposterior and mediolateral dimensions, and their general shapes resemble a clover”.

Some topics relating to large ornithopod tracks are currently under discussion: for example, the taxonomic affinity of their trackmakers (e.g., [16, 20]); whether the trackmakers were biped or quadrupeds [21–22]; and the ichnotaxonomy (e.g., [16, 18, 23–24]). This last point is analysed in the present study.

Díaz-Martínez et al. [25] suggested that most of the more than 40 large ornithopod ichnotaxa defined so far are not valid. According to Hunt and Lucas [26], there are several problems associated with the classification of this kind of tracks. For example, new ichnotaxa have been defined on the basis of poorly-preserved tracks and without diagnostic features (ichnotaxobases) [23], with an inadequate diagnosis [24], or on the basis of temporal and/or geographical criteria [27]. Early Cretaceous tracks have been considered different from Late Cretaceous ones because the former were impressed by “iguanodontids” and the latter by hadrosaurids [24, 27]. As a result, the large ornithopod tracks from the Early Cretaceous have usually been assigned to *Iguanodontipus*, *Amblydactylus* and *Caririchnium*, and those from the Late Cretaceous to *Hadrosauropodus* [26]. Lockley et al. [18] included the ichnotaxon *Ornithopodichnus* from the Early Cretaceous and *Jiayinosauropus* from the Late Cretaceous. In the last years, the need for a comprehensive review of large ornithopod tracks is deemed worthy of consideration [17, 23–25]. In a recent paper of Lockley et al. [18], which was published at the same time that the present work was under revision, the ichnotaxonomy of large ornithopod ichnospecies is reviewed, but several comments should be made (see below).

Here we present an exhaustive work, with special emphasis on the systematic ichnology of large ornithopod tracks and its geographic and temporal implications.

### Brief historical background

The first discovery of large ornithopod tracks occurred in 1846, when the Reverend Edward Tagart presented a single tridactyl footprint from the Wealden (Early Cretaceous) near Hastings, Sussex (England) to the Geological Society of London; Tagart’s accompanying letter included the statement that “Dr. Harwood suspects them to be the footprints of the *Iguanodon*” ([1], see [23, 28]). After this, other researchers found further large ornithopod tracks in England (e.g., [2–5]). Initially, Beckles [2–3] related them with *Ornithoidichnites* (a classification based on Hitchcock [29], who thought that he was classifying bipedal bird tracks), but subsequently Beckles [4] and Tylor [5] considered them to be *Iguanodon* tracks. In Germany, the earliest reports were published by Struckmann [30] and Grabbe [31], who classified the tracks as *Ornithoidichnites*, and Ballerstedt [32–34], who assigned them to *Iguanodon* tracks. Dollo [35] studied the foot bones of several *Iguanodon* skeletons from Bernissart (Belgium) and reconstructed the foot osteology of the possible trackmaker of the English footprints.

In Europe, large ornithopod tracks were regarded as *Iguanodon* footprints for a long time (e.g., [36–37] in England; [38–39] in Spain). Outside Europe, Zhen et al. [40] used this terminology for an ichnite from China. With the aim of giving a formal ichnotaxonomical name to this kind of footprint, Sarjeant et al. [23] published a synthetic work on the European tracks assigned to *Iguanodon*, and defined the ichnogenus *Iguanodontipus*.

The term “hadrosaur track” is more recent than “*Iguanodon* track”. It was used to describe the large ornithopod tracks found in the Upper Cretaceous rocks of North America. The first author to use the term was Langston [9] after studying a large Maastrichtian footprint from Alberta, Canada. He regarded it as a hadrosaurian ichnite on the basis of morphological differences relative to *Iguanodon* tracks and because of the discovery of a hadrosaurian skeleton near the site. Subsequently, other researchers described hadrosaur tracks (e.g., [24, 41–42]).

Moreover, other nomenclatural alternatives have been proposed for denominating large ornithopod tracks, such as “iguanodont”, “iguanodontid”, “iguanodontian”, “iguanodontoid” and “hadrosaurid” footprints (see [16, 19, 23, 41, 43–44], and references therein).

The first ichnotaxa defined on the basis of large ornithopod tracks were the ichnogenera *Amblydactylus*, *Gypsichnites* and *Dinosauropodes*, the former two described by Sternberg [8] and the latter one by Strevell [45]. Subsequently, Kuhn [46] defined *Wealdenichnites* and *Sinoichnites* on the basis of material previously studied by Dietrich [47] and Young [48] respectively.

## Material and Method

**Fig 1:**
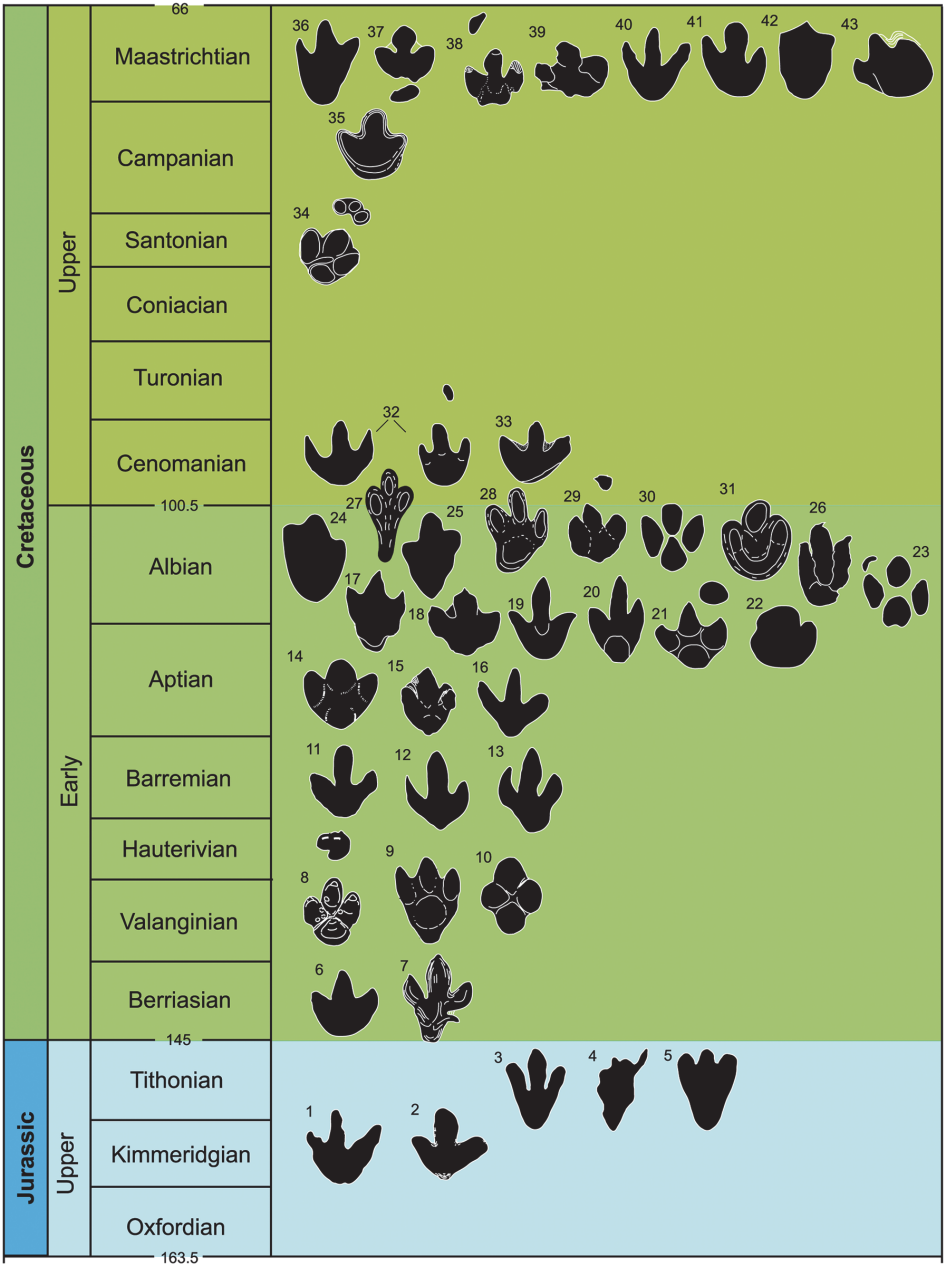
Temporal distribution of the large ornithopod ichnotaxa (outline drawings of holotypic tracks). Tracks are not to scale. 1. *Camptosauropus vialovi* (redrawn from [74]); 2. *Sinoichnites youngi* (redrawn from [46]); 3. *Kharkushosauropus kharkushensis* (redrawn from [56]); 4. *Iguanodonichnus frenkii* (redrawn from [70]); 5. *Camptosaurichnus fasolae* (redrawn from [70]); 6. *Iguanodontipus burreyi* (redrawn from [23]); 7. *Wealdenichnites iguanodontoides* (redrawn from [46]); 8. *Caririchnium magnificum* (redrawn from [11]); 9. *Gigantoshiraminesauropus matsuoi* (redrawn from [82]); 10. *Sousaichnium pricei* (redrawn from [10]); 11. *Staurichnium diogenis* (redrawn from [10]); 12. *Brachyguanodonipus prejanensis* (redrawn from [68]); 13. *Hadrosaurichnoides igeensis* (redrawn from [92]); 14. *Iguanodonipus cuadrupedae* (redrawn from [68]); 15. *Shiraminesauropus reini* (redrawn from [93]); 16. *Shiraminesauropus hayashidaniensis* (redrawn from [93]); 17. *Amblydactylus gethingi* (redrawn from [8]); 18. *Amblydactylus kortmeyeri* (redrawn from [57]); 19. *Gypsichnites pacensis* (redrawn from [8]); 20. *Iguanodonopus xingfuensis* (redrawn from [102]); 21. *Iguanodontipus billsarjeanti* (redrawn from [105]); 22. *Ornithopodichnus masanensis* (redrawn from [20]); 23. *Caririchnium kyoungsookimi* (redrawn from [80]); 24. *Akmechetosauropus makhkamovi* (redrawn from [56]); 25. *Babatagosauropus bulini* (redrawn from [56]); 26. *Yangtzepus yipingensis* (redrawn from [7]); 27. *Bonaparteichnium tali* (redrawn from [66]); 28. *Sousaichnium monettae* (redrawn from [66]); 29. *Caririchnium leonardii* (redrawn from [76]); 30. *Caririchnium lotus* (redrawn from [81]); 31. *Limayichnus major* (redrawn from [66]); 32. *Caririchnium protohadrosaurichnos* (redrawn from [78]); 33. *Jiayinosauropus johnsoni* (redrawn from [107]); 34. *Apulosauripus federicianus* (redrawn from [62]); 35. *Hadrosaurichnus titicaensis* (redrawn from [96]); 36. *Hadrosaurichnus australis* (redrawn from [93]); 37. *Hadrosauripeda hauboldi* (redrawn from [98]); 38. *Hadrosauropodus langstoni* (redrawn from [24]); 39. *Hadrosauropodus nanxiongensis* (redrawn from [99]); 40. *Orcauichnites garumniensis* (redrawn from [111]); 41. *Ornithopodichnites magna* (redrawn from [111]); 42. *Taponichnus donottoi* (redrawn from [119]); 43. *Telosichnus saltensis* (redrawn from [119]). The ichnogenus *Goseongosauripus kimi* has not been included because it was not possible to find a drawing of its holotype in the literature.

In the present paper, the ichnotaxa assigned to large ornithopod tracks (*Iguanodon* and hadrosaur tracks, iguanodont tracks, etc.) or associated with large ornithopod trackmakers have been revised. In total, 34 ichnogenera and 44 ichnospecies of large ornithopod tracks have been studied (Fig. 1, Table 1).

**Table 1:** Data on large ornithopod ichnotaxa.

Ichnotaxon	Reference		Age	Country
***Akmechetosauropus makhkamovi***		[56]	Albian	Tajikistan
***Amblydactylus gethingi***		[8]	Aptian-Albian [91]	Canada
***Amblydactylus kortmeyeri***		[57]	Aptian-Albian [91]	Canada
***Apulosauripus federicianus***		[62]	Santoniense	Italy
***Babatagosauropus bulini***		[56]	Albian	Tajikistan
***Bonaparteichnium tali***		[66]	Albian-Cenomanian?	Argentina
***Brachyguanodonipus prejanensis***		[68]	Basal Barremian-middle Albian [124]	Spain
***Camptosaurichnus fasolae***		[70]	Thitonian [71]	Chile
***Camptosauropus vialovi***		[74]	Upper Jurassic [75]	Tajikistan
***Caririchnium magnificum***		[11]	Berriasian-Hauterivian [146]	Brazil
***Caririchnium leonardii***		[76]	Albian-Cenomanian	USA
***Caririchnium protohadrosaurichnos***		[78]	Cenomanian	USA
***Caririchnium lotus***		[81]	“mid” Cretaceous	China
***Caririchnium kyounsookimi***		[80]	Upper Albian	Korea
***Gigantoshiraminesauropus matsuoi***		[82]	Hauterivian-Barremian [83]	Japan
***Goseongosauripus kimi***		[85]	Aptiense-Albiense [86]	Korea
***Gypsichnites pacensis***		[8]	Aptian-Albian [91]	Canada
***Hadrosaurichnoides igeensis***		[92]	Basal Barremian-middle Albian [124]	Spain
***Hadrosaurichnus australis***		[93]	Maastrichtian	Argentina
***Hadrosaurichnus titicaensis***		[96]	Campanian-Maastrichtian	Peru
***Hadrosauripeda hauboldi***		[97]	Maastrichtian	Canada
***Hadrosauropodus langstoni***		[24]	Maastrichtian	Canada
***Hadrosauropodus nanxiongensis***		[99]	Maastrichtian	China
***Iguanodonichnus frenkii***		[70]	Thitonian [671]	Chile
***Iguanodonipus cuadrupedae***		[68]	Basal Barremian-middle Albian [124]	Spain
***Iguanodonopus xingfuensis***		[102]	Aptian-Albian [203]	China
***Iguanodontipus burreyi***		[23]	Berriasian	England
***Iguanodontipus billsarjeanti***		[105]	Lower-upper Albian	Switzerland
***Jiayinosauropus johnsoni***		[107]	Albian-Cenomanian	China
***Kharkushosauropus kharkushensis***		[56]	Thitonian	Tajikistan
***Limayichnus major***		[66]	Albian-Cenomanian?	Argentina
***Orcauichnites garumniensis***		[111]	Maastrichtian	Spain
***Ornithopodichnites magna***		[111]	Maastrichtian	Spain
***Ornithopodichnus masanensis***		[20]	Albian	Korea
***Shiraminesauropus reini***		[82]	Hauterivian-Barremian [83]	Japan
***Shiraminesauropus hayashidaniensis***		[82]	Hauterivian-Barremian [83]	Japan
***Sinoichnites youngi***		[47]	Upper Jurassic [204]	China
***Sousaichnium pricei***		[10]	Berriasian-Hauterivian [123]	Brazil
***Sousaichnium monettae***		[65]	Albian-Cenomanian?	Argentina
***Staurichnium diogenis***		[10]	Berriasian-Hauterivian [123]	Brazil
***Taponichnus donottoi***		[119]	Maastrichtian	Argentina
***Telosichnus saltensis***		[119]	Maastrichtian	Argentina
***Wealdenichnites iguanodontoides***		[47]	Berriasian [103]	Germany
***Yangtzepus yipingensis***		[62]	Upper Lower Cretaceous [99]	China

Data on large ornithopod ichnotaxa, including the author, age and country.

*Ornithopodichnites* has not been considered in this study since 19th-century researchers classified all bipedal dinosaur tracks in this way in accordance with the approach of Hitchcock [29]. The proposal put forward by Strevell [45] has not been followed because the author defined one ichnogenus and eight ichnospecies on the basis of differently shaped natural casts removed from mines and without giving a diagnosis (see [49]).

The papers in which large ornithopod ichnotaxa were defined have been analysed taking into account the diagnosis, holotypes (photographs and drawings), type locality and type horizon when possible (Table 1, S1 Text). In addition, the ichnotaxonomic approaches of Sarjeant [50], Lockley et al. [51], Lockley et al. [24], Romero et al. [27], Bertling et al. [52], Demathieu and Demathieu [53], Díaz-Martínez et al. [54] and Lockley et al. [18] have been followed in determining the validity of these ichnotaxa. The main proposals from these papers that we consider relevant to the present analysis are:

a) If possible, the holotype and paratypes should be deposited in an official collection and should be available to the public. When it is impossible to deposit the original tracks, an artificial cast should be deposited.
b) The holotype must be an elite track, a very well-preserved true track or a natural cast that reproduces the anatomical features of the sole of the dinosaur pes and /or manus. Thus, ichnotaxa defined from tracks on the basis of extramorphological features, or from tracks that are poorly preserved or not impressed in the layer where the animal stepped (undertracks, underprints, etc.), should be avoided.
c) The holotype should not be the only track assigned to the ichnotaxon and, if possible, it should belong to a trackway. In the ichnological literature there are many ichnotaxa that are based on unique and isolated material, whose validity is uncertain. Tracks from the same trackway or other tracks of the type series show the intraichnotaxonomic variation.
d) New ichnotaxa should not be described until all the bibliography has been revised. Geographical or temporal distribution should not be considered discriminatory criteria.
e) The diagnosis of a new ichnotaxon must be as accurate as possible to avoid ambiguity. Moreover, it is important to add a detailed description (qualitative and quantitative) of the tracks that are used in the type series.
f) The diagnosis should be based on the morphological features of the tracks. Trackway data such as pace length, degree of rotation, external and internal width, etc. are not valid ichnotaxobases because they reflect dinosaur behaviour, and may be variable.
g) The description of the tracks must include good, perpendicular photographs and drawings of the holotype and paratypes. It is also recommended to publish drawings and photographs of other tracks and trackways assigned to the ichnotaxon.
h) The nomenclature of the trace fossils follows the International Code of Zoological Nomenclature (Fourth edition 1999; ICZN).

Taking into account these points, the ichnotaxa analysed in this work have been classified as follows: valid ichnotaxa, non-ornithopod ichnotaxa, *nomina nuda* and *nomina dubia*. The ichnotaxa without a formal definition (unpublished texts) or without any definition have been considered *nomina nuda*. The ichnotaxa based on unique, isolated, poorly-preserved tracks or with an ambiguous diagnosis have been classified as *nomina dubia*. The ichnotaxa based on tracks that do not show the morphology of the sole of the dinosaur pes (or manus) because they are affected by extramorphological features (taphotaxon *sensu* Lucas [55]), or impressed in a stratigraphic layer different from where the animal stepped (undertrack, underprint, etc.), have also been considered *nomina dubia*. The ichnotaxa that can be assigned to other kind of tracks such as theropods, thyreophorans, sauropods, etc. (*sensu* [19, 27]) have been classified as non-ornithopod. Finally, the ichnotaxa that comply with the main proposals suggested above are classified as valid ichnotaxa and their systematic affinity will be discussed (synonyms, amendments, new combinations, etc.).

**Fig 2:**
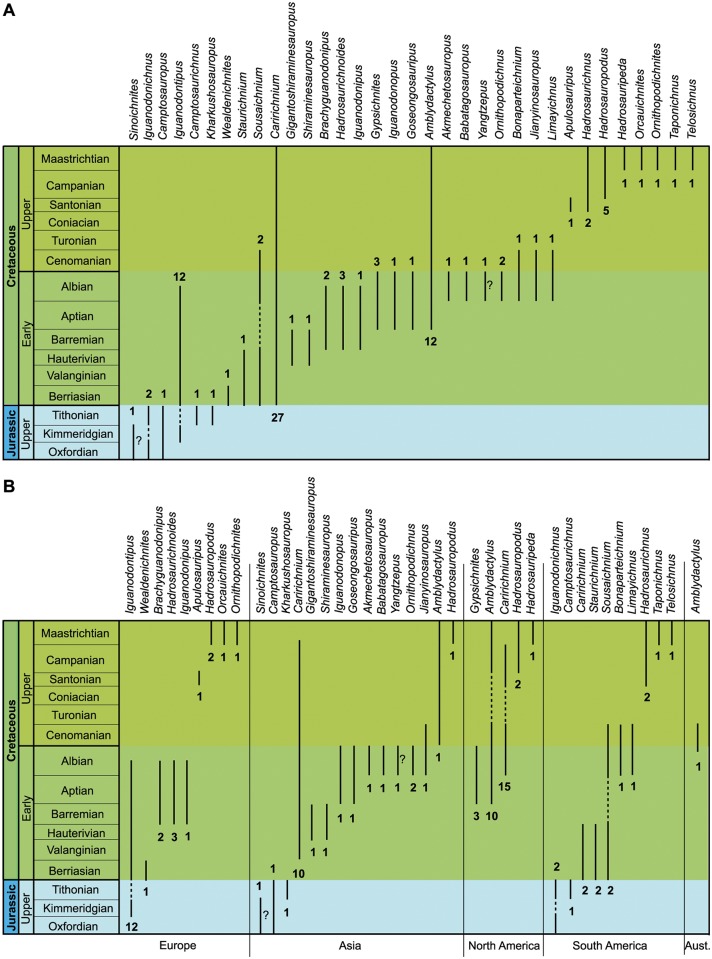
Distribution of studied large ornithopod ichnogenera. A, temporal distribution; B, temporal distribution by continents. Based on the data from S1 Table. Discontinuous line, there are no data.?, doubtful data. Aus., Australia.

All the articles in which tracks have been assigned to large ornithopod ichnotaxa have been analysed. The data obtained have been studied taking into account temporal and geographical points of view and the factor of abundance (Fig. 2, S1 Table).

### Institutional abbreviations

BC, British Columbia Provincial Museum, Victoria, Canada; BNSS, Geological Museum of Bournemouth Natural Science Society, Bournemouth, Hampshire, England, U.K.; NHCG, Natural Heritage Center (Geology), National Research Institute of Cultural Heritage, Daejeon, South Korea; QJGM, Exhibition Hall of Qijiang County Bureau of Land and Resources, China; NMB, Naturhistorisches Museum Basel, Basel, Switzerland; PMA, Royal Alberta Museum (formerly Provincial Museum of Alberta), Edmonton, Canada; TMP, Royal Tyrrell Museum of Palaeontology, Drumheller, Alberta, Canada.

## Results and Discussion

### General considerations concerning the studied ichnotaxa

In the literature, all the large ornithopod ichnogenera but six are monospecific (Fig. 1, Table 1). *Caririchnium* consists of five ichnospecies: *C*. *magnificum*, *C*. *leonardii*, *C*. *protohadrosaurichnos*, *C*. *lotus* and *C*. *kyoungsookimi*. The other ichnogenera include two ichnospecies: *Amblydactytus* (*A*. *gethingi* and *A*. *kortmeyeri*); *Hadrosaurichnus* (*H*. *australis* and *H*. *titicaensis*); *Hadrosauropodus* (*H*. *langstoni* and *H*. *nanxiongensis*); *Iguanodontipus* (*I*. *burreyi* and *I*. *billsarjeanti*); and *Sousaichnium* (*S*. *pricei* and *S*. *monettae*).

Large ornithopod ichnotaxa have been identified from the Late Jurassic to the Maastrichtian (Fig. 2A). The temporal distribution shows that there are five stages in which the joint presence of several ichnogenera has been cited. In the Berriasian, tracks are assigned to five different ichnogenera, four of which started their distribution in this stage. Eigth ichnogenera have been cited in the Barremian, and four of them occur for the first time in this stage. There are nine ichnogenera in the Aptian, and three of them have their origin in this stage. In the Albian, 17 ichnogenera have been cited, seven of which start in this stage. Finally, in the Maastrichtian there are nine ichnogenera, five of which have been described only in this stage. At least 19 out of the 34 ichnogenera of large ornithopod footprints are grouped in the Barremian-Albian period.

Of the 34 ichnogenera, 23 have been cited only once (in the paper where they are defined), five in two articles, two in three works, and one in seven papers (see S1 Table). Only three ichnogenera have over 10 citations: *Iguanodontipus* and *Amblydactylus* 13, and *Caririchnium* 29.

Concerning the distribution by continents (Fig. 2B, S1 Table), ten ichnogenera have been identified in South America, and five in North America. Six of these ichnotaxa have only one citation, five have two citations and one ichnotaxon has five citations. The ichnotaxa most abundantly cited are *Amblydactylus* and *Caririchnium*, with 10 and 17 (15 in North America and 2 in South America) citations respectively. In Asia 15 ichnogenera have been identified, 13 of which have one citation, while *Ornithopodichnus* has two, and *Caririchnium* 10. In Europe, eleven ichnogenera have been analysed. Five of them have been cited only once, *Brachyguanodonipus*, *Hadrosauropodus* and *Caririchnium* have been cited twice, *Hadrosaurichnoides* three times, and *Iguanodontipus* 13 times. Finally, in Australia the only ichnogenus mentioned is *Amblydactylus*.

Almost all the ichnogenera have been identified only in one continent (Fig. 2B). Nevertheless, *Caririchnium* has been cited in Europe, North America, South America and Asia (from Berriasian to Maastrichtian), *Amblydactylus* in Europe, North America, Asia and Oceania (from Barremian to Maastrichtian), and *Hadrosauropodus* in North America, Asia and Europe (from Campanian to Maastrichtian).

Of the 44 ichnospecies studied in this work, more than half (27) were described in the 1980s and 1990s (Table 1). In the 1990s alone 17 ichnospecies were erected. Large ornithopod ichnotaxa have been described in 15 different countries. China and Argentina are the countries where most ichnospecies have been described (six in each). Five ichnospecies have been described in both Spain and Canada, and four in Tajikistan. Brazil, Korea and Japan have three ichnospecies, Chile and the United States two, and the other countries (Germany, Italy, Peru, the United Kingdom and Switzerland) only one.

### Validity of large ornithopod ichnotaxa

***Akmechetosauropus makhkamovi* Dzhalilov and Novikov**, [56]

**Fig 3:**
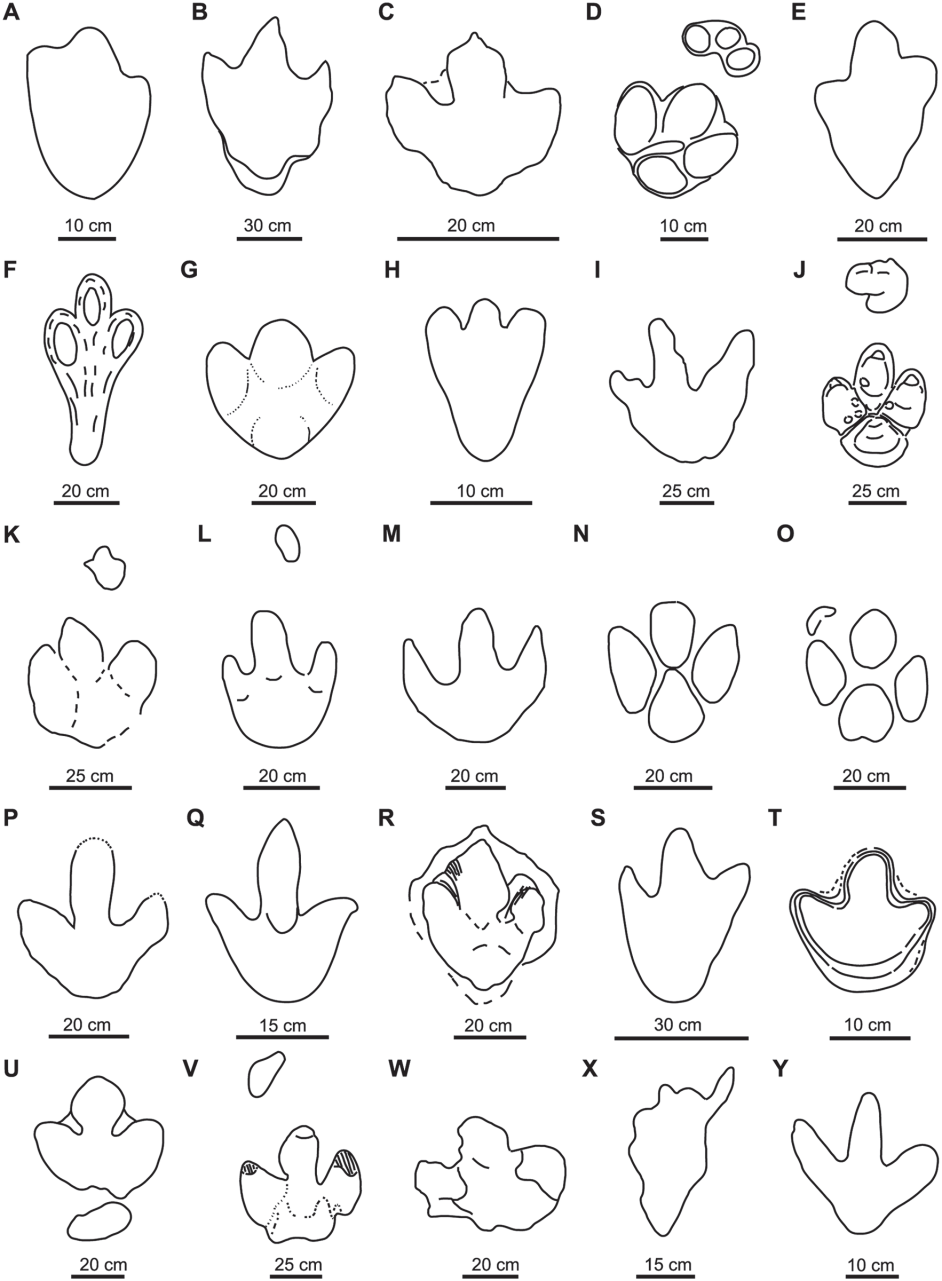
Holotypes of studied large ornithopod ichnotaxa. A, *Akmechetosauropus makhkamovi* (redrawn from [56]); B, *Amblydactylus gethingi* (redrawn from [8]); C, *Amblydactylus kortmeyeri* (redrawn from [57]); D, *Apulosauripus federicianus* (redrawn from [62]); E, *Babatagosauropus bulini* (redrawn from [56]); F, *Bonaparteichnium tali* (redrawn from [66]); G, *Brachyguanodonipus prejanensis* (redrawn from [68]); H, *Camptosaurichnus fasolae* (redrawn from [70]); I, *Camptosauropus vialovi* (redrawn from [74]); J, *Caririchnium magnificum* (redrawn from [11]); K, *Caririchnium leonardii* (redrawn from [76]); L-M, *Caririchnium protohadrosaurichnos* (redrawn from [78]); N, *Caririchnium lotus* (redrawn from [81]); O, *Caririchnium kyoungsookimi* (redrawn from [80]); P, *Gigantoshiraminesauropus matsuoi* (redrawn from [82]); Q, *Gypsichnites pacensis* (redrawn from [8]); R, *Hadrosaurichnoides igeensis* (redrawn from [92]); S, *Hadrosaurichnus australis* (redrawn from [93]); T, *Hadrosaurichnus titicaensis* (redrawn from [96]); U, *Hadrosauripeda hauboldi* (redrawn from [98]); V, *Hadrosauropodus langstoni* (redrawn from [24]); W, *Hadrosauropodus nanxiongensis* (redrawn from [99]); X, *Iguanodonichnus frenkii* (redrawn from [70]); Y, *Iguanodonipus cuadrupedae* (redrawn from [68]).

This was described as a middle-sized track from the Albian of Tajikistan that belongs to the ichnofamily Hadrosauripodidae (sensu [56]). The diagnosis is not precise, the figure of the holotype (Fig. 3A) is very schematic and the authors suggest that the footprints are poorly preserved. For these reasons, we consider this ichnotaxon to be a *nomen dubium*.

***Amblydactylus gethingi* Sternberg**, [8]

This ichnospecies was established with a footprint found in the Aptian-Albian of Canada [57], which is currently under the waters of the W.A.C. Bennett Dam [41]. A cast of the holotype (plastotype) was deposited in the National Museum of Canada [8]. The track (Fig. 3B) is quite deep, has no pad impressions, the distal end of the digits is acuminate, and there is a depression in the proximal part of the heel interpreted as the metatarsal impression [8]. Lockley et al. [18] suggested that this ichnotaxon is poorly defined. Currie and Sarjeant [57] have pointed out that after several surveys in the area where *A*. *gethingi* was defined they have not found any footprints attributable to this ichnotaxon. In later works, Currie [41, 43] assigned bipedal trackways to this ichnospecies.

Currie and Sarjeant [57] emended the diagnosis of *Amblydactylus* when defining the ichnospecies *A*. *kortmeyeri*. They proposed as diagnostic features the heel pad and the digit pad impressions. These features are evident in *A*. *kortmeyeri*, but Sternberg [8] noted that there were no pad impressions in the footprint of *A*. *gethingi*. Moreover, Sternberg [8] and Currie and Sarjeant [57] suggested that *Amblydactylus* had interdigital webs. According to Lockley et al. [24], there is no real evidence of these structures in ornithopods. Consequently, some footprints from New Mexico with web impressions have been reinterpreted as mud structures [58]. Thus, this feature should be interpreted as an extramorphological structure. Currie [43] referred quadruped trackways with bilobed pes to *Amblydactylus* isp., but this character was not previously assigned to either *A*. *gethingi* or *A*. *kortmeyeri*.

Currie [41] pointed out that *Irenesauripus occidentalis* Sternberg, [8] is a synonym of *Amblydactylus gethingi*. *I*. *occidentalis* was defined in the same work as *A*. *gethingi*, but on a previous page. In this case, *Amblydactylus* would be a junior synonym of *Irenesauripus*. On the other hand, Currie [43] considered that *Amblydactylus* was a senior synonym of *Caririchnium*, Lockley [59] suggested that *Amblydactylus* and *Caririchnium* were senior synonyms of *Iguanodontipus*, and Gierlinski et al. [60] stated that *Iguanodontipus* was a junior synonym of *Amblydactylus*.

Taking into account the above data on *A*. *gethingi*, we consider it not valid. The holotype shape is likely conditioned by extramorphological features (metatarsal mark, collapsed digit impressions, interdigital webs and absence of pad marks). Currie and Sarjeant [57] affirmed that the differences between the *A*. *gethingi* and *A*. *kortmeyeri* could be explained as differences in the circumstances of track formation. Moreover, although a plastotype is preserved in the National Museum of Canada, the holotype is under the waters of a dam. As suggested by Gangloff et al. [61], a neotype found close to the type locality would be advisable for further systematic discussions. Therefore, pending further works confirming the validity of *A*. *gethingi*, it is provisionally considered a *nomen dubium*.

***Amblydactylus kortmeyeri* Currie and Sarjeant**, [57]

This ichnotaxon was defined in the same formation as *A*. *gethingi* (Gething Formation, Aptian-Albian, Canada). The type series, tracks and trackways, are well preserved. The tracks (Fig. 3C) are tridactyl, with pointed digits, a rounded heel impression, one pad in each digit [57], and the diagnosis reflects the main features of the tracks. Therefore, we consider that this ichnotaxon is valid.

***Apulosauripus federicianus* Nicosia, Marino, Mariotti, Muraro, Panigutti, Petti and Sacchi**, [62]

This quadrupedal ichnotaxon was described in the Santonian of Italy. The type series (Fig. 3D) is well preserved, and the diagnosis is accurate. *Apulosauripus* was first related to hadrosaurs [62], but subsequent studies have suggested that a thyreophoran is the possible trackmaker [63–65]. Accordingly, we consider it a non-ornithopod ichnotaxon.

***Babatagosauropus bulini* Dzhalilov and Novikov**, [56]

This ichnotaxon was described in the Albian of Tajikistan and assigned to the ichnofamily Hadrosauripodidae [56]. Due to its insufficient diagnosis, its poor state of preservation (according to the authors) and the schematic drawing of the holotype (Fig. 3E) figure, we consider it a *nomen dubium*.

***Bonaparteichnium tali* Calvo**, [66]

This was found in the Albian-Cenomanian? of Argentina. *Bonaparteichnium* is mainly characterized by a long, wide and robust heel impression [66]. Calvo [67] stated that *Bonaparteichnium* tracks are conditioned by the dinosaur gait, which impressed the metatarsus when walking. He considered this ichnotaxon a *nomen vanum* and junior synonym of *Limayichnus*. The metatarsal impression of *Bonaparteichnium* implies that the footprint shape (Fig. 3F) is conditioned by the behaviour of the dinosaur and/or features of the substrate. Therefore, this ichnotaxon is a taphotaxon (*sensu* [55]) and should not be considered ichnotaxonomically valid. We regard it as a *nomen dubium*.

***Brachyguanodonipus prejanensis* Moratalla**, [68]

This ichnotaxon (Fig. 3G) is based on tracks from the Early Cretaceous of La Rioja in Spain. It was described in the unpublished doctoral thesis of Moratalla [68]. Díaz-Martínez et al. [69] regarded it as one of four morphotypes of *Iguanodon*-like footprints found in the Enciso Group (Cameros Basin). We consider it a *nomen nudum* because it has never been described formally.

***Camptosaurichnus fasolae* Casamiquela**, [70]

This was defined on the basis of Early Cretaceous tracks from Chile. Casamiquela and Fasola [70] classified *Camptosaurichnus* within the family Iguanodontidae. The footprints (Fig. 3H) are long and narrow, and show manus impressions. The heel impressions are acuminate and the digit impressions are narrow and sinuous, with claw marks [70]. The authors suggested that these features are due to the action of the mud. Moreno and Rubilar [71] assigned several tracks to this ichnotaxon. Sarjeant et al. [23] interpreted the morphology of the footprints as typically theropod. Nevertheless, Moreno and Pino [72] and later Moreno and Benton [73] maintained the ornithopod affinity of this ichnotaxon. We agree with Lockley et al. [24] in suggesting that the tracks of *C*. *fasolae* are poorly preserved and, therefore, in considering the ichnotaxon a *nomen dubium*.

***Camptosauropus vialovi* Gabunia and Kurbatov**, [74]

This ichnotaxon was described from the Late Jurassic of Tajikistan [75], and was assigned to the ichnofamily Iguanodontopodidae [74]. We consider *Camptosauropus vialovi* a *nomen dubium* because the diagnosis is insufficient and the holotype (Fig. 3I) has not been adequately figured.

***Caririchnium magnificum* Leonardi**, [11]

This was defined on the basis of a quadruped trackway from the Early Cretaceous of Brazil. Leonardi [11] proposed different diagnoses for the ichnogenus and the ichnospecies. The tracks (Fig. 3J) are well preserved. The pes tracks are large, tridactyl, with one pad impression in each digit and one in the heel, and they have short, wide digits. The manus tracks are smaller than the pes tracks. The ichnospecies diagnosis is very detailed and accurately reflects the morphology of the tracks. We consider *C*. *magnificum* to be a valid ichnotaxon.

***Caririchnium leonardii* Lockley**, [76]

This ichnotaxon (Fig. 3K) is based on a quadruped trackway from the “mid”—Cretaceous of the USA. The pes tracks are tridactyl, with one pad impression in each digit and one in the heel. The heel is not well marked (dashed line in Fig. 3K). The manus track is smaller than the pes track. In terms of the preservation of the type series, *C*. *leonadii* could be considered doubtful. Nevertheless, taking into account that these tracks can be related to other well-preserved tracks referred to *Caririchnium* or *Caririchnium leonardii* (e.g., [16, 42, 77]), we consider that this ichnotaxon is valid and that the diagnosis can be emended on the basis of the type series and referred tracks.

***Caririchnium protohadrosaurichnos*** [78]

This ichnotaxon (Fig. 3L–M) was based on six trackways from the Cenomanian of the USA. *C*. *protohadrosaurichnos* was defined by the position of the manus impression, which was different from that of other *Caririchnium* ichnospecies [78]. Hunt and Lucas [79] and Lim et al. [80] have suggested that the position of the manus track is not a valid ichnotaxonomic character because it is conditioned by the dinosaur’s behaviour. Therefore, we propose that *C*. *protohadrosaurichnos* is a *nomen dubium*.

***Caririchnium lotus* Xing, Wang, Pan and Chen**, [81]

This ichnospecies was defined by Xing et al. [81] from about 200 tracks found in the “mid”-Cretaceous of China. The tracks (Fig. 3N) are tridactyl, with one pad impression in each digit and one in the heel, which is rounded. The tracks are well preserved, and the diagnosis describes the the track shape. Therefore, we consider that this ichnotaxon is valid.

***Caririchnium kyoungsookimi* Lim, Lockley and Kong**, [80]

This ichnotaxon (Fig. 3O) is based on two manus-pes pairs found in a slab of stone from the “mid”-Cretaceous of Korea. The pes tracks are tridactyl, have a bilobed heel and one pad impression in the heel and one in each digit. Lim et al. [80] suggested that the morphology of the manus track is diagnostic. Accordingly, they defined *C*. *kyoungsookimi* mainly on the basis of differences in the manus shape relative to other *Caririchnium* ichnospecies. The tracks are well preserved, and the diagnosis conforms to the track shape. Thus, we consider that this ichnotaxon is valid.

***Gigantoshiraminesauropus matsuoi* Azuma and Takeyama**, [82]

The isolated and poorly-preserved track (Fig. 3P) used by Azuma and Takeyama [82] to define *G*. *matsuoi* was found in the Early Cretaceous of Japan. Matsukawa et al. [83] noted that this ichnotaxon was described without making a comparison with the footprints from other localities. Lockley and Matsukawa [84] considered *Gigantoshiraminesauropus* a *nomen dubium*, and we agree with this conclusion.

***Goseongosauripus kimi* Kim**, [85]

This ichnotaxon is based on a track from the Early Cretaceous of Korea. It was defined in an abstract without a diagnosis [86]. Subsequently, the same author changed the name *Goseongosauripus kimi* to *Koseongosauripus onychion* [87], but still failed to provide any diagnosis. Lockley et al. [86] considered the ichnotaxa proposed by Kim to be invalid. According to Lockley et al. [86], *Goseongosauripus* could be a junior synonym of *Amblydactylus* or *Caririchnium*. Moreover, Kim’s [87] work is unpublished, and the footprint that he figures shows several pad impressions in each digit, so it does not belong to an ornithopod. We agree with Lockley et al. [86] that *Goseongosauripus* is a *nomen nudum*.

***Gypsichnites pacensis* Sternberg**, [8]

This was defined from an isolated track (Fig. 3Q) from the “mid”-Cretaceous of Canada [8]. Some authors have defended the ornithopod affinities of this ichnotaxon [88–89]. Nevertheless, the footprint morphology (longer than wide) and the acuminate distal end of the digits (claw marks) suggest that the trackmaker is a theropod [61, 90–91]. After analysing the figures and descriptions of *G*. *pacensis*, we agree with the second interpretation and regard it as a theropod ichnotaxon.

***Hadrosaurichnoides igeensis* Casanovas, Ezquerra, Fernández, Pérez-Lorente, Santafé and Torcida**, [92]

This ichnotaxon (Fig. 3R) was described on the basis of more than 200 tracks from the Early Cretaceous of Spain [92]. It is primarily characterized by interdigital web impressions. Casanovas et al. [92] suggested that the trackmaker was a transitional form between iguanodontids and hadrosaurids. Lockley et al. [24] interpreted the trackmaker as a theropod and classified *Hadrosaurichnoides* as a *nomen dubium*. Díaz-Martínez et al. [69] considered this ichnotaxon to be one of four morphotypes of *Iguanodon*-like tracks found in the Enciso Group (Cameros Basin). According to Lockley et al. [24], there is no evidence of an interdigital web in the ornithopod foot. As noted by Lockley and Hunt [58], the supposed web may be the result of extramorphological factors. *Hadrosaurichnoides* was impressed in calcareous sediment with a planar lamination produced by algal mats parallel to the stratification [92]. The algal mats are occasionally broken by the weight of the dinosaur, creating a discontinuity in the mat (I.D.-M., pers. obs.). For this reason, *Hadrosaurichnoides* is a taphotaxon, and we regard it as a *nomen dubium*.

***Hadrosaurichnus australis* Alonso**, [93]

This ichnotaxon (Fig. 3S) is based on four trackways from the Maastrichtian of Argentina [93]. Lockley et al. [24] suggested that *H*. *australis* shows theropod features (elongate footprint with relatively slender, tapering digit impressions). According to these authors, the paratype illustrated by Psihoyos and Knoebber [94] corresponds to a theropod. Sarjeant et al. [23] considered that *Kuwajimasauropus*, described as a theropod by Azuma and Takeyama [82], is a junior synonym of *Hadrosaurichnus*. Huh et al. [95] compared some tracks that they considered to be theropod with *Hadrosaurichnus*. Nevertheless, Thulborn [19] has suggested that *H*. *australis* is a hadrosaur ichnotaxon. In sum, we have classified it as a *nomen dubium* due to the ambiguity of the diagnosis and the poor quality of the holotype figure in the original article.

***Hadrosaurichnus titicaensis* Ellenberger**, [96]

Jaillard et al. [96] defined *H*. *titicaensis* from the Campanian-Maastrichtian of Peru as tracks that are longer than wide and without digital pad impressions (Fig. 3T). They related this ichnotaxon with a hadrosaur because the tracks preserve impressions of rigid interdigital tissues (web) and have blunt digits. As occurs with *Amblydactylus* and *Hadrosaurichnoides*, the web impressions can be explained as extramorphological features [58]. The holotype exhibits a similar morphology to that of an ornithopod track. Nevertheless, the ambiguity of the diagnosis and the lack of diagnostic characters in the holotype figure raise doubts regarding the validity of this ichnotaxon. Therefore, we propose that *H*. *titicaensis* be considered a *nomen dubium*.

***Hadrosauripeda hauboldi* Vialov**, [97]

This ichnotaxon was erected from a footprint found in the Late Cretaceous of Canada. No diagnosis is given. Vialov [97] based his proposal on a track (Fig. 3U) figured by Haubold [98] and regarded as a hadrosaur footprint by Langston [9]. We consider *Hadrosauripeda* to be a *nomen nudum*.

***Hadrosauropodus langstoni* Lockley, Nadon and Currie**, [24]

This ichnotaxon (Fig. 3V) was defined to propose a valid name for the hadrosaur tracks found in the Maastrichtian of Canada [24]. The tracks of *H*. *langstoni* are quadrupedal, with large, tridactyl pes tracks that have one pad impression in each digit and one in the heel, which is bilobed. This ichnotaxon is defined on the basis of well-preserved material [18] and an adequate diagnosis. We consider it a valid ichnotaxon.

***Hadrosauropodus nanxiongensis* Xing, Harris, Dong, Lin, Chen, Gou and Ji**, [99]

*H*. *nanxiongensis* is based on footprints (Fig. 3W) from the Maastrichtian of China. According to Xing et al. [99], the tracks differ from those of *H*. *langstoni* in the size of digit II (larger in *H*. *nanxiongensis*) and in the divarication between digits II and IV (higher in *H*. *nanxiongensis*). Xing et al. [99] suggested that *H*. *nanxiongensis* has a notch in the proximal part of digit II that is absent in *H*. *langstoni*. They also stated that the size of the digit II impression is not an extramorphological feature. Nevertheless, Xing et al. [99] assigned to this ichnotaxon the isolated cast of a track that has the digit IV impression narrower than that of the other digits, less divarication than in the holotype and with notches in the proximal part of digits II and IV. The holotype is based on a rather poorly-preserved trackway [18, 100]. The digital and heel pads are not well marked. The isolated cast is more similar to *H*. *langstoni* than to *H*. *nanxiongensis*. Based on the data and figures of Xing et al. [99], all these footprints can be assigned to the ichnogenus *Hadrosauropodus*. However, none of these tracks is sufficiently well preserved to allow the erection of a new ichnotaxon. For this reason, we consider that *H*. *nanxiongensis* is a *nomen dubium*.

***Iguanodonichnus frenkii* Casamiquela**, [70]

This ichnotaxon (Fig. 3X) is based on a long trackway from the Berriasian of Chile. Moreno and Rubilar [71] considered that *Iguanodonichnus* is a *nomen dubium* and assigned it to cf. *Parabrontopodus*. Sarjeant et al. [23] suggested that the footprints are similar to sauropod tracks. Moreno and Benton [73] proposed a new combination and denominated it *Parabrontopodus frenki* (with one i). Casamiquela and Fasola’s [70] descriptions are ambiguous, and the illustrations are oblique photographs, without scale diagrams to illustrate the footprint morphology adequately [23]. As the tracks may belong either to a sauropod or an ornithopod, in this work *I*. *frenkii* is considered to be a *nomen dubium* due to its insufficient diagnosis and the poor quality of the holotype figures.

***Iguanodonipus cuadrupedae* Moratalla**, [68]

This ichnotaxon (Fig. 3Y), based on Early Cretaceous tracks from La Rioja in Spain, was described by Moratalla [68] in his unpublished doctoral thesis. Sarjeant et al. [23] referred part of these footprints to *Iguanodontipus*. Díaz-Martínez et al. [69] considered *I*. *cuadrupedae* to be one of the four morphotypes of *Iguanodon*-like footprints found in the Enciso Group (Cameros Basin). Pascual-Arribas et al. [101] regarded *Iguanodonipus* as not valid. We consider this ichnotaxon to be a *nomen nudum* because it has not yet been described formally.

***Iguanodonopus xingfuensis* Zhen, Li and Hang**, [102]

**Fig 4:**
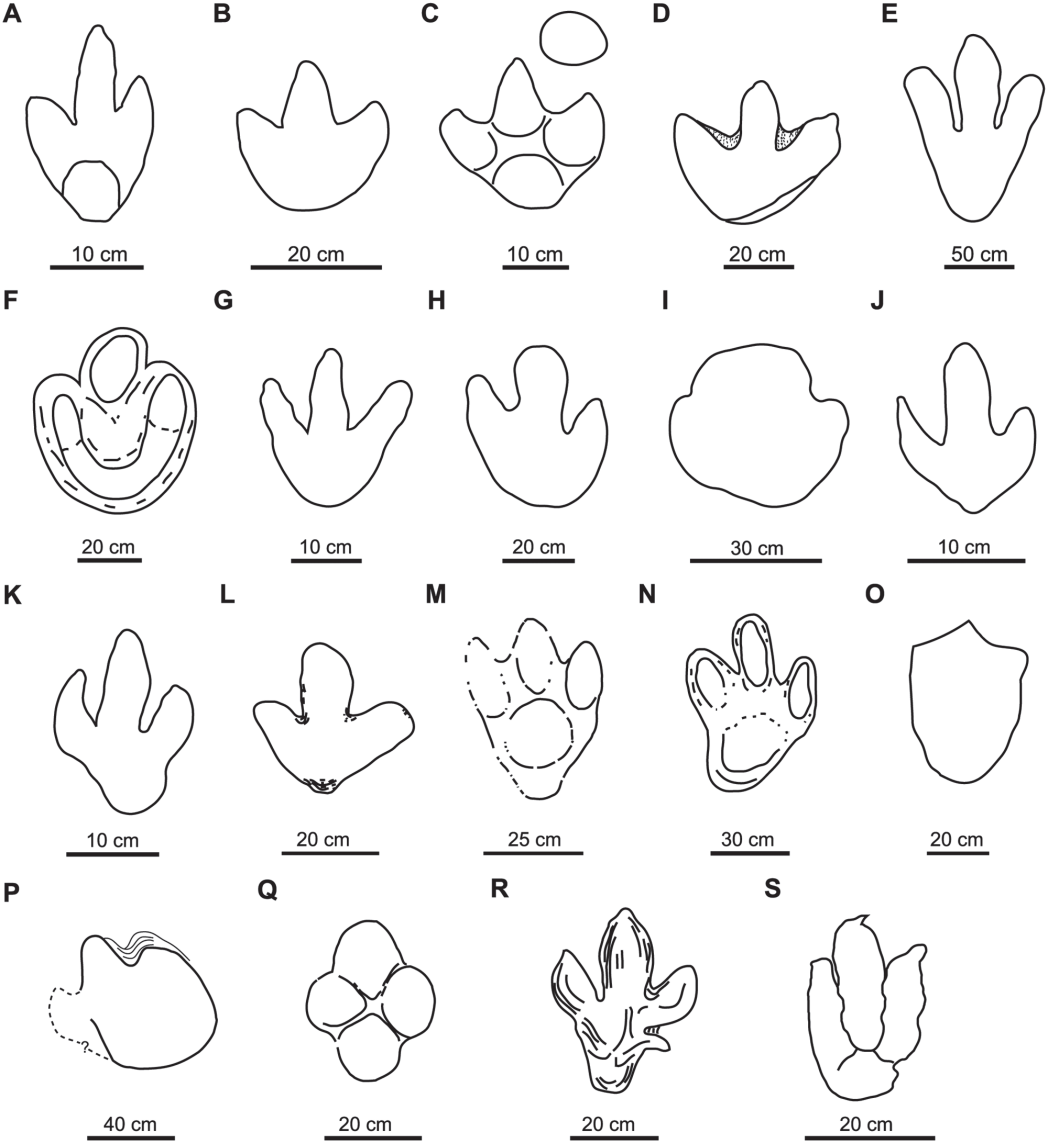
Holotypes of studied large ornithopod ichnotaxa (cont.). A, *Iguanodonopus xingfuensis* (redrawn from [102]); B, *Iguanodontipus burreyi* (redrawn from [23]); C, *Iguanodontipus billsarjeanti* (redrawn from [105]); D, *Jiayinosauropus johnsoni* (redrawn from [107]); E, *Kharkushosauropus kharkushensis* (redrawn from [56]); F, *Limayichnus major* (redrawn from [66]); G, *Orcauichnites garumniensis* (redrawn from [111]); H, *Ornithopodichnites magna* (redrawn from [111]); I, *Ornithopodichnus masanensis* (redrawn from [20]); J, *Shiraminesauropus reini* (redrawn from [82]); K, *Shiraminesauropus hayashidaniensis* (redrawn from [82]); L, *Sinoichnites youngi* (redrawn from [47]); M, *Sousaichnium pricei* (redrawn from [10]); N, *Sousaichnium monettae* (redrawn from [66]); O, *Staurichnium diogenis* (redrawn from [10]); P, *Taponichnus donottoi* (redrawn from [119]); Q, *Telosichnus saltensis* (redrawn from [119]); R, *Wealdenichnites iguanodontoides* (redrawn from [47]); S, *Yangtzepus yipingensis* (redrawn from [7]).

This ichnotaxon was described from two isolated tracks (Fig. 4A) from the Early Cretaceous of China. Sarjeant et al. [23] considered that these footprints are too long in relation to their width to be included among large ornithopod footprints. We agree with Xing et al. [99] that the diagnosis and discussion of *Iguanodonopus* are insufficient, and we therefore consider it a *nomen dubium*.

***Iguanodontipus burreyi* Sarjeant, Delair and Lockley**, [23]

*Iguanodontipus burreyi* (Fig. 4B) was erected in order to group the tracks found in Europe (England, Germany and Spain) that had previously been assigned to *Iguanodon* [23]. The type series of *I*. *burreyi* consists of several natural casts that are not well preserved (see 23, [figs. 13–14]). The diagnosis adequately reflects the morphology of these tracks. Subsequently, other ornithopod tracks found in Europe have been referred to *Iguanodontipus* (e.g., [103–104]), but their shape is not identical to the holotype and they have not corresponded to the diagnosis for *I*. *burreyi*. Lockley et al. [18, 23] noted that the type material for *I*. *burreyi* is moderately well preserved and is properly described, so they considered it a valid ichnotaxa. However, Meyer and Thüring [105] stated that the outline of the footprints is poorly-preserved, and Diedrich [103] suggested that the type trackway of *Iguanodontipus* is similar to that of *Megalosauropus*. Moreover, Pascual-Arribas et al. [101] stated that the diagnosis of *Iguanodontipus* should be revised.

As regards the preservation of the type series, *I*. *burreyi* could be considered a *nomen dubium*. Nevertheless, it should be noted that these tracks can be related to other well-preserved tracks referred to as *Iguanodontipus* or “*Iguanodon* tracks” (e.g., [16, 106]), so we consider that *I*. *burreyi* is valid and propose to amend the diagnosis on the basis of these tracks.

***Iguanodontipus billsarjeanti* Meyer and Thüring**, [105]

This ichnotaxon (Fig. 4C) was defined on the basis of three quadruped trackways from the Early Cretaceous of Switzerland. The tracks are well preserved and exhibit one pad impression in the heel and one in each digit. Meyer and Thüring [105] noted that *I*. *billsarjeanti* differs from *I*. *burreyi* in having a well-defined contour line and a higher divarication. The holotype of *I*. *billsarjeanti* is well preserved, and the diagnosis is accurate. Therefore, we consider it a valid ichnotaxon.

***Jiayinosauropus johnsoni* Dong, Zhou and Wu**, [107]

This ichnotaxon is based on an incomplete footprint (Fig. 4D) found in the Late Cretaceous of China. Xing et al. [99] and Lockley et al. [100] suggested that *Jiayinosauropus* is similar to *Hadrosauropodus*. For these authors the ichnotaxon is valid but it should be revised in the future. Lockley et al. [18] noted that the morphology of *Jiayinosauropus* is insufficiently known. It was defined on the basis of a single, poorly-preserved track and the diagnosis is inadequate. Therefore, we consider it a *nomen dubium*.

***Kharkushosauropus kharkushensis* Dzhalilov and Novikov**, [56]

This ichnotaxon (Fig. 4E) was described from the Tithonian of Tajikistan and included within the ichnofamily Iguanodontopodidae [56]. The tracks have long digit impressions with acuminate distal ends and slight inward rotation. These features are more typical of theropods than ornithopods. We agree with Lockley et al. [108] in considering that *Kharkushosauropus* is a *nomen dubium*.

***Limayichnus major* Calvo**, [66]

This ichnotaxon (Fig. 4F) was defined on the basis of tracks from the “mid”-Cretaceous of Argentina. Meyer [109] stated that *Limayichnus* is a theropod track, and regarded *Bonaparteichnium* and *Sousaichnium* as junior synonyms of *Limayichnus*. Calvo [110] based the ornithopod affinities of *L*. *major* on the absence of claw marks. Finally, Apesteguía and Gallina [65] suggested that *Limayichnus* is a theropod ichnotaxon and related it with carcharodontosaurids. We consider that *L*. *major* is a *nomen dubium* due to the absence of diagnostic features that allow it to be assigned either to theropod or ornithopod ichnotaxa.

***Orcauichnites garumniensis* Llompart, Casanovas and Santafé**, [111]

This ichnotaxon (Fig. 4G) was described from several poorly-preserved tracks found in the Maastrichtian of Spain. Lockley et al. [24] suggested that the tracks are probably theropod footprints because they are longer than wide. These authors considered that *Orcauichnites* is a *nomen dubium*, and we accept this interpretation.

***Ornithopodichnites magna* Llompart, Casanovas and Santafé**, [111]

*Ornithopodichnites* (Fig. 4H) was described in a site close to *Orcauichnites*. According to Lockley and Meyer [112] and Lockley et al. [24], *Ornithopodichnite*s has the same problems as *Orcauichnites*. For these authors, the tracks are poorly preserved, the original description is inaccurate, and the tracks belong to theropods. Therefore, *O*. *magna* is considered to be a *nomen dubium*.

***Ornithopodichnus masanensis* Kim, Lockley, Kim, Lim and Kim**, [20]

This ichnotaxon (Fig. 4I) was described from the Early Cretaceous of Korea on the basis of robust, tridactyl, slightly mesaxonic footprints that are wider than long, with short, U-shaped digit impressions that have a blunt distal end, and with a smoothly rounded heel. Kim et al. [20] argued that the tracks are suitable for defining a new ichnotaxon because they are true footprints (presence of extruded rims) and the general shape is the same in more than 100 footprints. Nevertheless, Lockley et al. [113] assigned to *Ornithopodichnus* footprints with a morphology that differs from that of the type series and does not conform with the original diagnosis. The presence of extruded rims is not an unequivocal character of true tracks (cf. [114]). Lockley et al. [18] noted that the preservation of these trackways is suboptimal, with some tracks still partially filled. Kim et al. [20] affirmed that preservational factors likely play a role in the morphology. The footprints described by Kim et al. [20] and Lockley [115] (including photographs and drawings) have no indisputable diagnostic features. The general morphology is similar to that of other tracks assigned to *Hadrosaurichnoides* that were defined on the basis of extramorphological features. Pending a revision of the validity of *O*. *masanensis*, in this work we consider that this ichnotaxon is a *nomen dubium*.

***Shiraminesauropus reini* Azuma and Takeyama**, [82]

Azuma and Takeyama [82] described the ichnospecies *Shiraminesauropus reini* (Fig. 4J) and *S*. *hayashidaniensis* (Fig. 4K) on the basis of two isolated footprints from the Early Cretaceous of Japan. Matsukawa et al. [83] noted that the material is probably inadequate and undiagnostic, it was not adequately compared with other footprints, and the differences in preservation were not taken into consideration. Lockley and Matsukawa [84] considered that both ichnospecies of *Shiraminesauropus* are *nomina dubia*. We agree with this interpretation.

***Shiraminesauropus hayashidaniensis* Azuma and Takeyama**, [82]

As discussed above, *S*. *hayashidaniensis* is regarded as a *nomen dubium*.

***Sinoichnites youngi* Kuhn**, [46]

This ichnotaxon is based on a single track (Fig. 4L) from the Late Jurassic of China [46], which was originally described by Teilhard and Young [6]. *S*. *youngi* has been assigned to Iguanodontidae by Kuhn [46] and Zhen et al. [116]. The diagnosis by Kuhn [46] is very imprecise. The exact age and origin of the track is unknown [117]. The footprint is currently lost, and only a cast is preserved in the Museum of Natural History of Beijing [99]. For these reasons, we consider that *Sinoichnites youngi* is a *nomen dubium*.

***Sousaichnium pricei* Leonardi**, [10]

Based on tracks from the Early Cretaceous of Brazil, this ichnotaxon (Fig. 4M) has been related to Iguanodontidae [10]. *Sousaichnium* shows elongate heel impressions, mud collapsed inside the track, and it lacks claw impressions. Pérez-Lorente [118] pointed out that the elongate impressions represent metatarsal marks. The tracks that Leonardi [10] assigned to *S*. *pricei* are not well preserved (e.g., metatarsal impressions, mud collapsed inside the track, etc.). Therefore, we consider it a *nomen dubium*.

***Sousaichnium monettae* Calvo**, [66]

This ichnotaxon (Fig. 4N) was defined on the basis of tracks from the “mid”-Cretaceous of Argentina, and related to Iguanodontidae [66]. Meyer [109] suggested that *Sousaichnium monettae* and *Bonaparteichnium tali* were junior synonyms of *Limayichnus major*, and regarded them as theropod tracks. Calvo [66, fig. 3] figured the holotype of *S*. *monettae* as being mainly characterized by a metatarsal impression. This impression can be caused by a special gait, sloping ground, the action of mud, etc. [118]. Irrespective of the affinities of the trackmaker (likely an ornithopod), we consider it a *nomen dubium*.

***Staurichnium diogenis* Leonardi**, [10]

This ichnotaxon (Fig. 4O) was based on tracks from the Early Cretaceous of Brazil. The footprints are only faintly impressed, which is probably due to the fact that the mud was dry when the dinosaur walked on it [10]. Leonardi [10] related *Staurichnium* with ornithopods such as Hadrosauridae. This ichnotaxon is characterized by the elongate heel impression. As the tracks are not well preserved and the ichnotaxon was defined on the basis of the metatarsal impressions, we consider that *Staurichnium* is a *nomen dubium*.

***Taponichnus donottoi* Alonso and Marquillas**, [119]

This ichnotaxon is based on an isolated track (Fig. 4P) from the Late Cretaceous of Argentina. The tracks are twice as long as wide, with short digit impressions and interdigital web marks. Due to the age (Late Cretaceous) and the interdigital web, Alonso and Marquillas [119] related *Taponichnus* with a medium to large-sized hadrosaur. The diagnosis of *T*. *donottoi* is imprecise and the material is a single, poorly-preserved footprint, so we consider it a *nomen dubium*.

***Telosichnus saltensis* Alonso and Marquillas**, [119]

This ichnotaxon from the Late Cretaceous of Argentina was based on a large, rounded track (Fig. 4Q), with no claw impressions, blunt digits II and III and extruded rims [119]. Alonso and Marquillas [119] suggested that *Telosichnus* is an ornithopod because the tracks show the features listed by Thulborn and Wade [120]. Moreover, they related the tracks to Hadrosauridae on the basis of their age and morphology. The figures are incomplete (dashed line in the drawing) and do not provide useful information. As with *Taponichnus*, the diagnosis is ambiguous and the material is poorly preserved and isolated. Therefore, we consider that *Telosichnus* is a *nomen dubium*.

***Wealdenichnites iguanodontoides* Kuhn**, [46]

The footprint (Fig. 4R) is a natural cast found in the Late Jurassic of Germany [46]. It is mainly characterized by having a hallux impression. The diagnosis is imprecise and the holotype is based on a single, isolated specimen. Therefore, we consider *Wealdenichnites* a *nomen dubium*.

***Yangtzepus yipingensis* Young**, [7]

This ichnotaxon (Fig. 4S) is based on tracks from the Early Cretaceous of China. Young [7] related it to an ornithopod. However, Xing et al. [99] interpreted the tracks as theropod footprints, similar to tracks found in the USA [121–122]. On the basis of the morphology of the tracks (longer than wide, with elongate, narrow digit impressions, and claw marks), we have classified them as theropod tracks.

### Morphology of valid large ornithopod ichnotaxa

Only eight of the 44 studied ichnospecies are considered valid: “*Amblydactylus*” *kortmeyeri*, *Caririchnium magnificum*, *Caririchnium leonardii*, *Caririchnium lotus*, *Caririchnium kyoungsookimi*, *Iguanodontipus burreyi*, *Iguanodontipus billsarjeanti* and *Hadrosauropodus langstoni* (Table 2, 3). According to the general shape of the impressions of both the heel and digits, the eight valid ichnospecies can be grouped into three main groups.

**Table 2:** Valuation of the large ornithopod ichnotaxa studied in this paper.

Assessment	Ichnotaxon
**Valid**	Caririchnium magnificum
	Caririchnium kortmeyeri
	Caririchnium billsarjeanti
	Caririchnium lotus
	Iguanodontipus burreyi
	Hadrosauropodus langstoni
	Hadrosauropodus leonardii
	Hadrosauropodus kyoungsookimi
***Nomina nuda***	Brachyguanodonipus prejanensis
	Goseongosauripus kimi
	Hadrosauripeda hauboldi
	Iguanodonipus cuadrupedae
***Nomina dubia***	Amblydactylus gethingi
	Akmechetosauropus makhkamovi
	Babatagosauropus bulini
	Bonaparteichnium tali
	Camptosaurichnus fasolae
	Camptosauropus vialovii
	Caririchnium protohadrosaurichnos
	Gigantoshiraminesauropus matsuoi
	Hadrosaurichnoides igeensis
	Hadrosaurichnus australis
	Hadrosaurichnus titicaensis
	Hadrosauropodus nanxiongensis
	Iguanodonichnus frenkii
	Iguanodonopus xingfuensis
	Jianynosauropus johnsoni
	Kharkushosauropus kharkushensis
	Limayichnus major
	Orcauichnites garumniensis
	Ornithopodichnites magna
	Ornithopodichnus masanensis
	Shiraminesauropus reini
	Shiraminesauropus hayashidaniensis
	Sinoichnites youngi
	Sousaichnium pricei
	Sousaichnium monettae
	Staurichnium diogenis
	Taponichnus donottoi
	Telosichnus saltensis
	Wealdenichnites iguanodontoides
**Non ornithopod**	Apulosauripus federicianus
	Gypsichnites pacensis
	Yangtzepus yipingensis

Valuation of the large ornithopod ichnotaxa studied in this paper: valid ichnotaxa, *nomina nuda*, *nomina dubia* and non ornithopod ichnotaxa. For valid ichnotaxa, see Table 3.

**Table 3:** Large ornithopod ichnotaxa considered valid in this study.

Previous works	In this work	Observations
***Amblydactylus kortmeyeri***	*Caririchnium kortmeyeri*	Comb. nov., emended
***Caririchnium magnificum***	*Caririchnium magnificum*	Emended
***Caririchnium leonardii***	*Hadrosauropodus leonardii*	Comb. nov., emended
***Caririchnium lotus***	*Caririchnium lotus*	Emended
***Caririchnium kyoungsookimi***	*Hadrosauropodus kyoungsookimi*	Comb. nov., emended
***Hadrosauropodus langstoni***	*Hadrosauropodus langsotoni*	Emended
***Iguanodontipus burreyi***	*Iguanodontipus burreyi*	Emended
***Iguanodontipus billsarjeanti***	*Caririchnium billsarjeanti*	Comb. nov., emended

**Fig 5:**
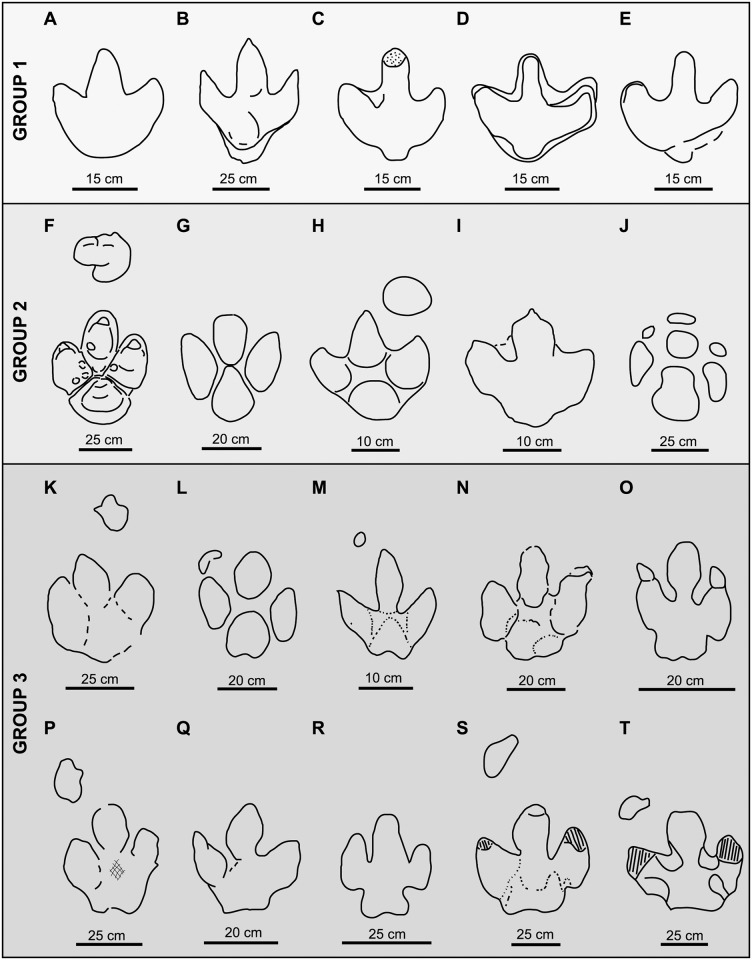
Groups of large ornithopod tracks classified on the basis of heel and digit impressions (see explanation in the text). Group 1: A, holotype of *Iguanodontipus burreyi* (redrawn from [23]); B, *Iguanodontipus burreyi* (redrawn from [3]); C-E, *Iguanodontipus* isp. (redrawn from [101]). Group 2: F, holotype of *Caririchnium magnificum* (redrawn from [11]); G, holotype of *Caririchnium lotus* (redrawn from [81]); H, holotype of *Iguanodontipus billsarjeanti* (redrawn from [105]); I, holotype of *Amblydactylus kortmeyeri* (redrawn from [57]); J, *Caririchnium* isp. (redrawn from [95]). Group 3: K, holotype of *Caririchnium leonardii* (redrawn from [76]); L, holotype of *Caririchnium kyoungsookimi* (redrawn from [80]); M, *Caririchnium leonardii* (redrawn from [100]); N, *Caririchnium* isp. (redrawn from [171]); O, *Caririchnium* isp. (redrawn from [167]); P, *Caririchnium leonardii* (redrawn from [43]); Q, *Caririchnium leonardii* (redrawn from [172]); R, *Caririchnium* isp. (redrawn from [95]); S, holotype of *Hadrosauropodus langstoni* (redrawn from [24]); T, *Hadrosauropodus langstoni* (redrawn from [24]).

Group 1: characterized by a small, rounded heel, with elongate, narrow digits. Only *Iguanodontipus burreyi* is included in this group (Fig. 5A).

Group 2: characterized by a large, rounded heel, with short, wide digits. *Caririchnium magnificum*, *Caririchnium lotus*, “*Amblydactylus*” *kortmeyeri*, *Iguanodontipus billsarjeanti* and some tracks of *Caririchnium leonardii* are included in this group (Fig. 5B).

Group 3: characterized by a large, bilobed heel, and short, wide digits. *Hadrosauropodus langstoni*, *Caririchnium kyoungsookimi* and some tracks of *Caririchnium leonardii* are included in this group (Fig. 5C).

*Caririchnium* has two ichnospecies in Group 2 (rounded heel) and two in Group 3 (bilobed heel). As regards *Iguanodontipus*, *I*. *burreyi* shows a small heel and elongate, narrow digits (Group 1), whereas *I*. *billsarjeanti* has a large heel and short, wide digits (Group 2). Finally, some tracks assigned to the ichnospecies *Caririchnium leonardii* have a rounded heel (Group 2), whereas other tracks show a bilobed heel (Group 3).

The presence of the same ichnospecies in two groups (Fig. 5) could be explained by: a) an inadequate diagnosis that has led to tracks with different characters being classified together; or b) geographical and temporal conventions (see below).

In order to simplify the ichnotaxonomy of large ornithopod tracks and to group the valid ichnospecies in ichnogenera with a stable morphology, the following systematic considerations have been made:

1. The diagnoses of the valid ichnogenera and ichnospecies have been amended;
2. Some ichnospecies have been assigned to other ichnogenera, and new combinations are here proposed.

### Systematic Ichnology

Ichnofamily **Iguanodontipodidae Vialov, [97] sensu Lockley, Xing, Lockwood and Pond, [18]**.

**Emended diagnosis**

Mesaxonic, tridactyl, subsymmetrical pes tracks that are as wide as or wider than long; one pad impression in each digit and one in the heel; digit pads longer than wide; well-developed notches in the proximal part of the digit II and IV impressions; manus tracks occasionally present and much smaller than the pes tracks.

**Type ichnogenus**

*Iguanodontipus* Sarjeant, Delair and Lockley, [23].

**Assigned ichnogenera**

*Caririchnium* Leonardi, [11]; *Hadrosauropodus* Lockley, Nadon and Currie, [24].

**Distribution**

Cretaceous, Berriasian to Maastrichtian. Europe, Asia, North America and South America (for details see the distribution of included ichnotaxa and referred material).

**Comments**

As recently suggested by Lockley et al. [18], we consider it necessary to propose a suprageneric ichnotaxon to include the valid large ornithopod ichnotaxa, as well as the tracks that share the same main features but are not well enough preserved to be assigned to a particular ichnogenus or ichnospecies. Vialov [97] proposed the ichnofamilies Iguanodontipodidae and Hadrosauripodidae. However, he defined the ichnofamilies without providing a diagnosis and without presenting the differences between them. Vialov included the ichnogenus *Hadrosauripeda* within Hadrosauripodidae, but did not include any ichnotaxa within Iguanodontipodidae. Subsequently, Gabunia and Kurbatov [74] and Dzhalilov and Novikov [56] respectively assigned *Camptosauropus* and *Kharkushosauropus* to Iguanodontopodidae instead of Iguanodontipodidae (probably a typographic mistake), and Dzhalilov and Novikov [55] assigned *Akmechetosauropus* and *Babatagosauropus* to Hadrosauripodidae. These proposals have not been used by other researchers in subsequent papers. Hadrosauripodidae is composed of ornithopod (*Hadrosauripeda* sensu [97]) and theropod (*Babatagosauropus* sensu [107]) morphotypes, and a morphotype of uncertain affinity (*Akmechetosauropus*). The ichnotaxa included in Iguanodontipodidae have theropod (*Kharkushosauropus* sensu [108]) and uncertain (*Camptosauropus*) affinities. Moreover, all these ichnogenera are considered in the present paper to be *nomina dubia* or *nomina nuda*. On the other hand, Lockley et al. [18] proposed a new ichnofamily using the nomenclature of Vialov [96] and Dzhalilov and Novikov [55]. In the text, they used the ichnofamily name Iguanodontipodidae, but in the ichnotaxonomical proposal, just before the diagnosis, used Iguanodontopodidae (probably a typographic mistake). According to the International Code of Zoological Nomenclature (art. 64) “the choice of type genus determines the stem of the name of the nominal family-group taxon”. In this case, the type ichnogenus is *Iguanodontipus*, and therefore the correct name of the ichnofamily is Iguanodontipodidae. We emend the diagnosis of Iguanodontipodidae on the basis of the shared morphology of the ichnotaxa here considered valid.

Lockley et al. [18] proposed *Amblydactylus*, *Caririchnium*, and *Iguanodontipus* as the unique ichnotaxa in the ichnofamily Iguanodontipodidae. In accordance with our study about the validity of large ornithopod ichnotaxa presented above, the type ichnospecies of *Amblydactylus*, *A*. *gethingi*, is a *nomen dubium*. Only the ichnogenera *Iguanodontipus*, *Caririchnium* and *Hadrosauropodus* are included within this ichnofamily, as are the tracks of some of the ichnotaxa that are here considered non-valid but that nonetheless present diagnostic features that allow them to be classified within Iguanodontipodidae (*Brachyguanodonipus*, *Gigantoshiraminesauropus*, *Hadrosaurichnoides*, *Hadrosauripeda*, *Hadrosauropodus nanxiongensis*, *Iguanodonipus*, *Limayichnus*, *Shiraminesauropus*, *Ornithopodichnus*, *Sousaichnium*, *Staurichnium* and *Wealdenichnites*).

Ichnogenus ***Iguanodontipus*** Sarjeant, Delair and Lockley, [23]

**Emended diagnosis**

Tracks belonging to Iguanodontipodidae with a small heel impression that is rounded, centred and narrow (as wide as the width of the proximal part of the digit III impression); long, narrow digit impressions with sharp distal ends.

**Type ichnospecies**

*Iguanodontipus burreyi* Sarjeant, Delair and Lockley, [23].

**Description**

All the information on the type series is in Sarjeant et al. [23].

**Distribution**

Lower Durlston Beds, Berriasian, England [23]; Bückeburg Formation, Berriasian, Germany [123]; Oncala Group, Berriasian-Valanginian, Spain (*sensu* [124]).

**Comments**

**Fig 6:**
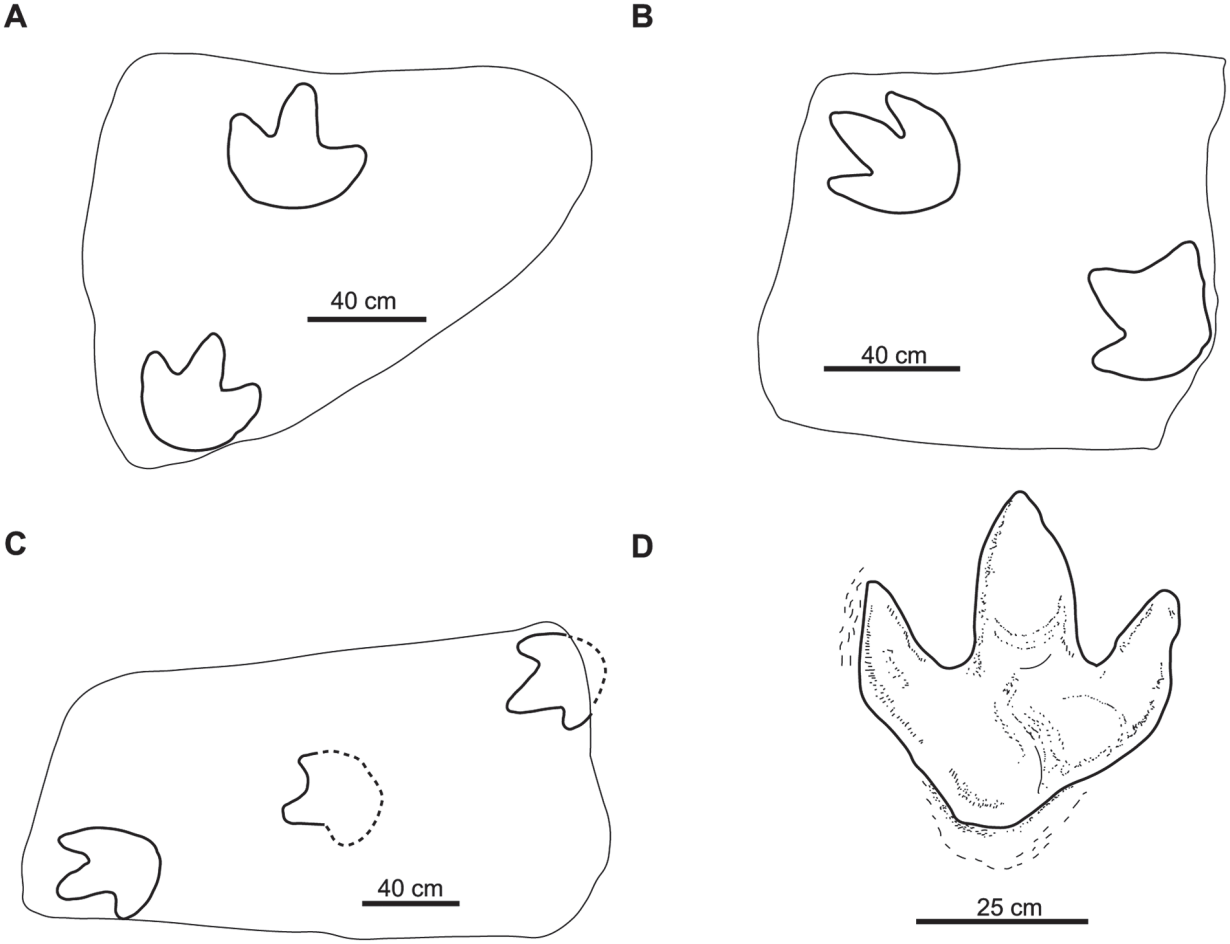
Tracks of Iguanodontipus. A-C, type series of *Iguanodontipus burreyi* (redrawn from [23]); D, referred track of *Iguanodontipus burreyi* (redrawn from [3]).

*Iguanodontipus* was described by Sarjeant et al. [23] in order to include within a formal group the tracks that had previously been assigned to *Iguanodon*. The type series comprises seven casts made from tracks found in the Berriasian of England (Fig. 6A-C). The tracks have a large, rounded heel and three short, wide digits; pad and claw impressions are lacking. Meyer and Thüring [105] noted that the contour line of the tracks from the type series is poorly preserved. The diagnosis proposed by Sarjeant et al. [23] reflects the shape of the tracks of the type series, but these authors assigned to *Iguanodontipus* other footprints with a more complex morphology that did not correspond to these diagnostic features. In this context, researchers such as Gierlinski et al. [60] and Lucas et al. [16] have illustrated *Iguanodontipus* using a footprint of *Ornithoidichnites* that was studied originally by Beckles [3] (Fig. 6D) and was subsequently referred to *Iguanodontipus* by Sarjeant et al. [23] (fig. 3). This footprint is not deformed and, in contrast to those of the type series, has a narrow, rounded heel, and long and independent digit impressions with acuminate distal ends. The wide, rounded heel impression shown by the holotype of *I*. *burreyi* is due to the loss of the notch of the proximal part of digits II and IV. The triangular shape of the digits is likely the result of the distal displacement of the hypex in poorly-preserved footprints. Tracks with these features from the Berriasian of Britain [23], Germany [125] and Spain [101, 126] have been assigned to *Iguanodontipus*. Other footprints found in the Berriasian of Spain [67, 127–128] that exhibit the same features have been classified as *Therangospodus oncalensis*. Recently, Castanera et al. [129] referred them as *Iguanodontipus*? *oncalensis* and discussed their relation with the ichnogenus *Iguanodontipus*. These footprints show diagnostic features of *Iguanodontipus* and probably belong to this ichnogenus [126].

On the other hand, several tracks from the Late Jurassic-Early Cretaceous transition of Europe that lack the diagnostic features of *Iguanodontipus* have been assigned to this ichnogenus [104, 130–132], probably because of geographical and temporal conventions.

On the basis of tracks from the Aptian of Switzerland, Meyer and Thüring [105] described a second ichnospecies of *Iguanodontipus*, *I*. *billsarjeanti*. However, in the present paper we consider that the morphology of the heel and digits of these tracks is consistent with the diagnostic features of *Caririchnium* (see above).

Diedrich [103] proposed as paratypes of *Iguanodontipus burreyi* two quadruped trackways from Spain and Germany, and claimed that the ichnotaxon could be quadruped. Nevertheless, he did not emend the diagnosis of *I*. *burreyi*, and the proposed paratypes come from outside the type locality; therefore, this proposal has no ichnotaxonomic validity.

Lockley [59] suggested that *Iguanodontipus* is a junior synonym of *Amblydactylus* and *Caririchnium*. According to Lucas et al. [16], *Iguanodontipus* is a junior synonym of *Amblydactylus* and differs from *Caririchnium* in having graceful digits directed laterally and a narrower heel. Finally, Lockley et al. [18] noted that *Amblydactylus* and *Iguanodontipus* are not synonyms. As discussed above, we consider that the shape of the heel and digits allow the ichnogenera of Iguanodontipodidae to be differentiated from one another. *Iguanodontipus* is characterized by a narrow, rounded heel and long, narrow digit impressions. In contrast, *Caririchnium* has a wide, rounded heel and short, wide digit impressions, and *Hadrosauropodus* shows a wide, bilobed heel and short, wide digit impressions.

A few quadruped tracksites could be assigned to *Iguanodontipus*. Lockley et al. [133] studied some tracksites from the Berriasian of Germany that present manus tracks smaller than the pes tracks. The manus tracks are wider than long and are situated in front of the pes track close to digit IV.

In sum, we consider that *I*. *burreyi* is the only ichnospecies that belongs to *Iguanodontipus*; *I*. *billsarjeanti* is assigned to *Caririchnium*.

***Iguanodontipus burreyi*** Sarjeant, Delair and Lockley, [23]

**Diagnosis**

As for ichnogenus.

**Holotype**

BNSS 33793b [23] p. 194, fig. 12. Paratypes: BNSS 33793a and c [23] p. 194, fig. 12 (Fig. 6A-C).

**Type horizon**

Lower Durlston Beds (Middle Purbeck Beds), Early Cretaceous, Berriasian [23].

**Type locality**

Norman’s Quarry, Queensground, Langton Matravers, Dorset, England [23].

**Distribution**

As for ichnogenus.

**Synonymy**

1852 *Ornithoidichnites* [2], figs. 1–2.

1854 *Ornithoidichnites* [3], p. XIX.

1905 Empreintes d’*Iguanodon* [35], figs. 1–4.

1914 *Iguanodon* [33], fig. 1–2.

1958 *Iguanodon mantelli* [46], figs. 1, 18.

1971 *Iguanodon* [98], figs. 1–3, 5.

1980 *Iguanodon* footprint [134], photographs 8, 9.

1983 *Iguanodon* footprint [135], photograph 5.

1983 *Megalosaurus* footprint [135], photograph 7.

**Fig 7:**
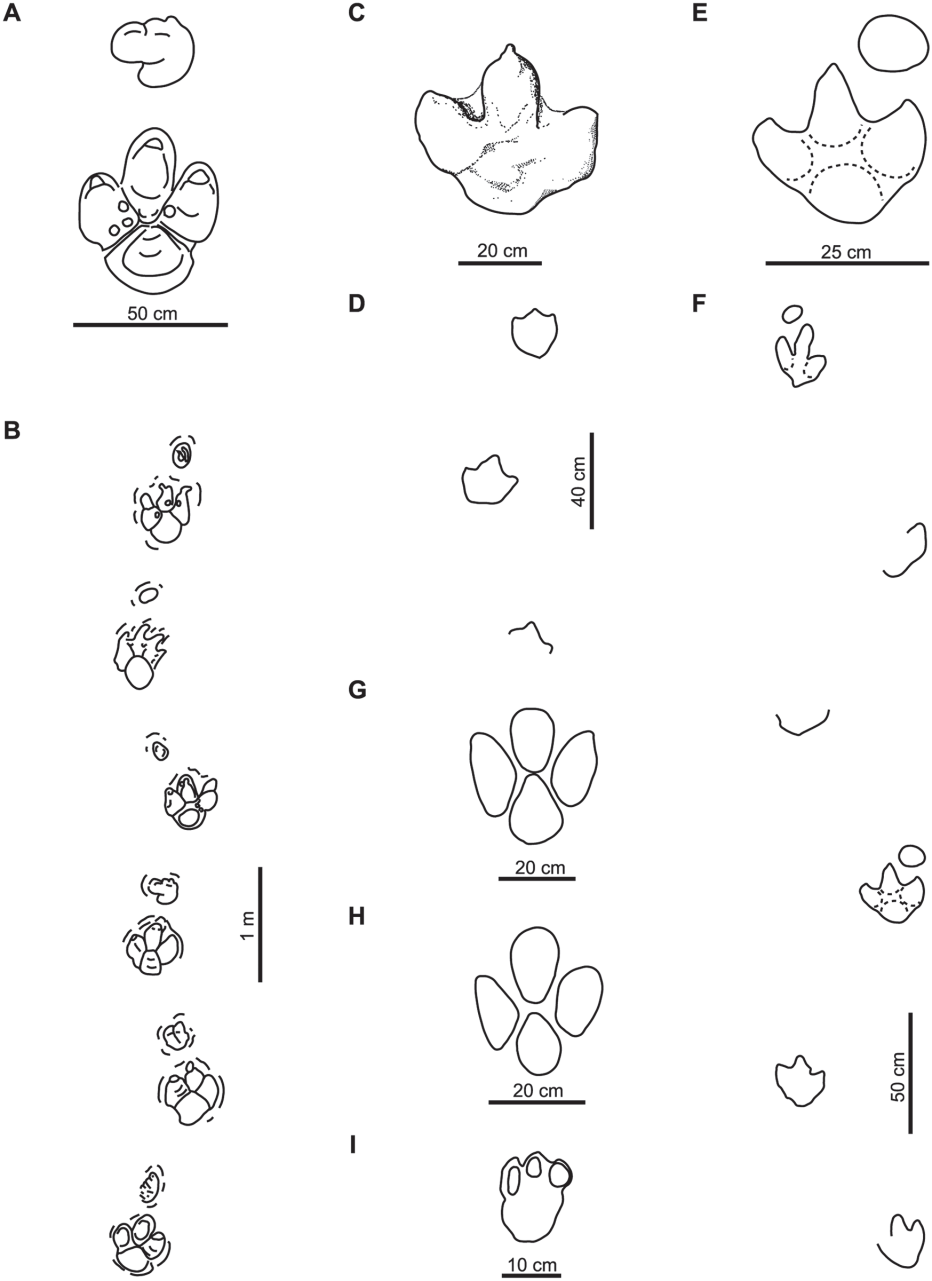
Tracks of Caririchnium. A-B, type series of *Caririchnium magnificum* (redrawn from [11]); C-D, type series of *Caririchnium kortmeyeri* (redrawn from [57]); E-F, type series of *Caririchnium billsarjeanti* (redrawn from [105]); G-I, type series of *Caririchnium lotus* (redrawn from [81]).

**Fig 8:**
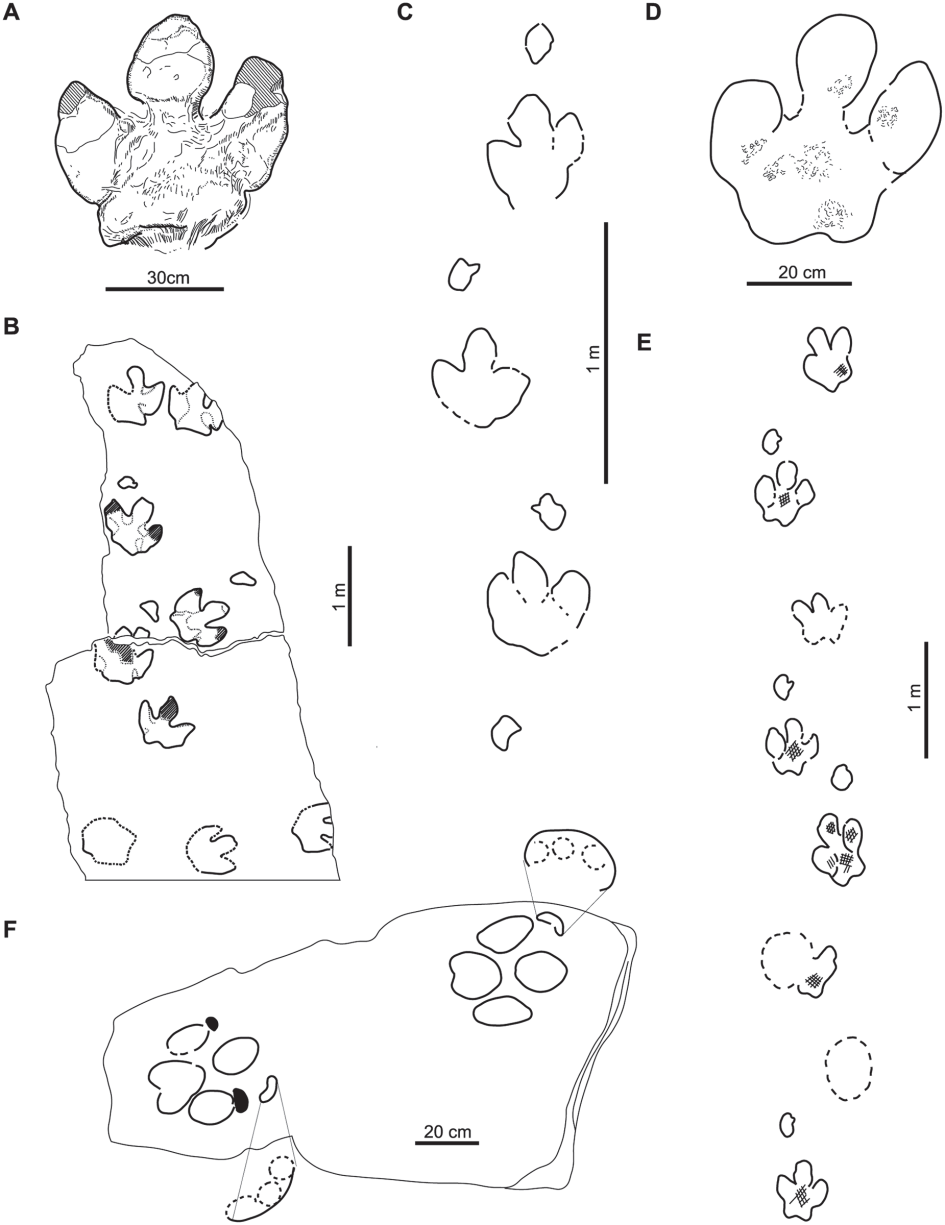
Tracks of *Hadrosauropodus*. A, holotype of *Hadrosauropodus langstoni* (redrawn from [24]); B, type series of *Hadrosauropodus langstoni* (redrawn from [24]); C, type series of *Hadrosauropodus leonardii* (redrawn from [76]); D, referred track of *Hadrosauropodus leonardii* (redrawn from [43]); E, referred trackway of *Hadrosauropodus leonardii* (redrawn from [43]); F, type series of *Hadrosauropodus kyoungsookimi* (redrawn from [80]).

**Fig 9:**
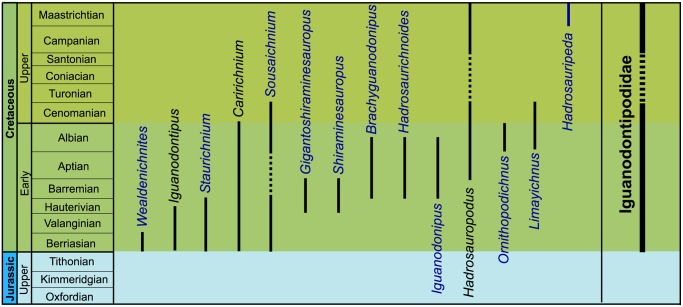
Temporal distribution of ichnogenera included in Iguanodontipodidae. The ichnogenera in black are considered systematically valid. The ichnogenera in blue are considered systematically non-valid but the tracks belong to Iguanodontipodidae (see explanation in the text). Discontinuous line, there are no data.

**Fig 10:**
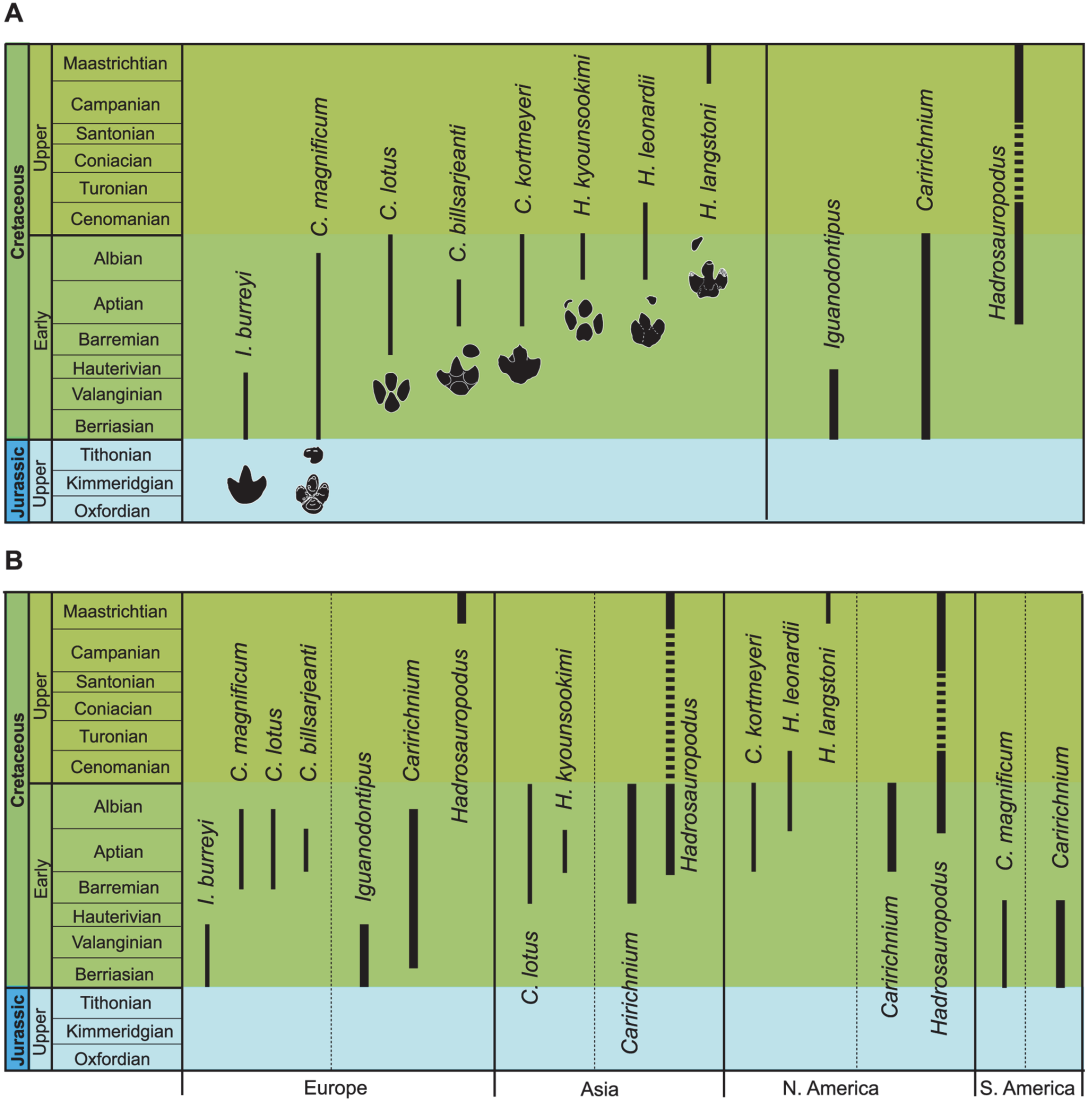
Distribution of valid ichnogenera and ichnospecies of Iguanodontipodidae. A, temporal distribution. Outline drawings of holotypic tracks (see references in Fig. 1); B, temporal distribution by continents. Discontinuous line, there are no data.

1985 *Iguanodon* footprints [136], figs. 6–10.

1989 *Iguanodon* [137], fig. 31.2 pro parte.

1990 *Iguanodon* footprints [19], figs. 6.32a, 6.33f.

1991 *Iguanodon* footprints [138], fig. 5.3 pro parte.

1993 *Therangospodus oncalensis* [68] ichnosp. nov. p. 106, 189–202, figs.8.5.1, 8.5.2, 8.6.1 (B), 8.6.2 (A).

1995 Ornithopod footprints [139], fig. 17, pro parte.

1998 *Iguanodontipus burreyi* [22], ichnogen. and ichnosp. nov. figs. 3, 12–14.

2000 *Therangospodus oncalensis* [127], p. 147–148, figs. 1b-c, 7.

2000 Theropod footprint [140], figs. 15, 20.

2001 Iguanodontid trackway [21], fig. 29.1b.

2002 Iguanodontid trackway [141], fig. 8.

2004 *Iguanodontipus* isp. [133], fig. 7.

2005 *Iguanodontipus* isp. [142], fig. 5E.

2005 *Therangospodus oncalensis* [143], fig. 9.

2005 Ornithopod footprint [143], fig. 15, 17.

2006 *Therangospodus oncalensis* [143], figs. 2–3, 4–5, 6.

2008 *Therangospodus oncalensis* [144], fig. 3.

2009 *Iguanodontipus* isp. [101], figs. 2, 4.

2011 *Iguanodontipus* [16], fig. 5 pro parte.

2012 *Iguanodontipus burreyi* [25], fig. 1A.

2012 Large ornithopod trackway [123], figs. 1, 9D-E.

2013 *Iguanodontipus*? *oncalensis* [129], figs. 3–11.

2013 *Iguanodontipus burreyi* [129], fig. 13D.

2013 *Iguanodontipus* [129] figs. 13I-J, M.

2014 *Iguanodontipus* [18], fig. 4A.

2014 *Iguanodon* footprint casts [18], fig. 5.

2014 *Iguanodontipus burreyi* [145], fig. 12D.

**Referred material**

Trackways from England, Germany and Spain. Material includes tracks from the Weald of England ([2] p. 396–398, figs. 1–2; [3] p. 458, pi. XIX; [23] p. 194–195, figs. 12–14; [142] p. 671, fig. 5E); the Bückeburg Formation of Germany ([33] 1914, p. 48–49, fig. 1–2; [21] p. 430, fig. 29.1b; [124] p. 269, fig. 7); and the Oncala Group of Spain ([139] p. 28–29, fig. 17. 3ST1–3, 6); [127] p. 344, fig. 1b-c, p. 347. fig. 7; [128] p. 239, figs. 2–3, p. 240, figs. 4–5, p. 243, fig.6.; [101] p. 109, fig. 2, p. 111, fig. 4; [129] p.7–14, figs. 4–11).

Ichnogenus ***Caririchnium*** Leonardi, [11]

**Emended diagnosis**

Pes tracks belonging to Iguanodontipodidae, with a large heel impression that is rounded, centred and wide (wider than the width of the proximal part of the digit III impression); short, wide digit impressions.

**Type ichnospecies**

*Caririchnium magnificum* Leonardi, [11].

**Assigned ichnospecies**

*Caririchnium kortmeyeri* (Currie and Sarjeant, [57]) comb. nov.; *Caririchnium billsarjeanti* (Meyer and Thüring, [105]) comb. nov.; *Caririchnium lotus* Xing, Wang, Pan and Chen, [81].

**Distribution (ichnospecies and referred material)**

Antenor Navarro Formation, Berriasian-Hauterivian, Brazil (*sensu* [146]); Salema Formation, Hauterivian-Barremian, Portugal [130]; Urbión Group (Cameros Basin), basal Valanginian-lower Aptian, Spain (*sensu* [124]); Wessex Formation, Barremian, England [145]; Enciso Group (Cameros Basin), basal Barremian-mid Albian, Spain (*sensu* [124]); Camarillas Formation, lower Barremian, Spain [131]; Abejar Formation, upper Barremian-Aptian, Spain [147]; Schrattenkalk Formation, Aptian, Switzerland [105]; Gething Formation, Aptian-Albian, Canada [43]; Jiaguan Formation, Barremian-Albian, China (*sensu* [148]).

**Comments**

When Leonardi [11] defined *Caririchnium* he proposed two separate diagnoses, one for the ichnogenus and the other for the ichnospecies. Leonardi [11] characterized the pes tracks of *Caririchnium* as large, tridactyl, with a pad impression in the heel, and short, wide digits. Moreover, the manus tracks are small and elliptical. Subsequently, Lockley [76] emended the ichnogeneric diagnosis, although basically he translated into English the original diagnosis in Italian by Leonardi. Lee [78] also emended the diagnosis of *Caririchnium*. He proposed different features for bipedal and quadrupedal trackways. Lee’s diagnosis is inaccurate and scarcely takes into account the morphology of the tracks. The three diagnoses of *Caririchnium* mostly used ichnotaxobases that depend on the values obtained from trackway analysis. It should be noted that trackway data depend mainly on dinosaur behaviour and are of little value for ichnotaxonomy [53]. The important features are those obtained from the shape of the pes and manus sole impressions.

Leonardi [11] and Lockley [76] claimed that *Caririchnium* is mainly characterized by quadruped trackways with the pes tracks much larger than the manus tracks, the pes tracks having one pad impression in each digit and one in the heel, and the pes digits being short and wide. Nevertheless, these authors do not consider the heel shape. In the present paper, two kinds of heel impression have been identified: rounded and bilobed (see [126]). *Caririchnium* and *Iguanodontipus* show rounded heel impressions whereas in *Hadrosauropodus* the heel is bilobed. *Caririchnium* has a large heel that is wider than the maximum width of the proximal part of digit III. On the other hand, *Iguanodontipus* has a small heel that is no wider than the maximum width of the proximal part of digit III. Moreover, the digit impressions of *Iguanodontipus* are elongate and narrow whereas *Caririchnium* and *Hadrosauropodus* have short, wide digit impressions. The differences in the impressions of both the heel and digits allow us to differentiate between the three ichnogenera of large ornithopod footprints.

Currie [43] suggested that *Caririchnium*, *Hadrosaurichnus* and *Ornithopodichnites* were junior synonyms of *Amblydactylus*. Nevertheless, in this work *Hadrosaurichnus*, *Ornithopodichnites* and *Amblydactylus* are regarded as *nomina dubia* (see discussion above). On the other hand, Lucas et al. [16] noted that the diagnoses of *Caririchnium* and *Hadrosauropodus* were similar and that there are no features that differentiate between them. Consequently, they proposed that *Caririchnium* is a senior synonym of *Hadrosauropodus*. Several authors (e.g., [79–80]) have considered that *Caririchnium* shows a bilobed heel impression. Nevertheless, as discussed above, *Caririchnium* has a rounded heel whereas the heel impression of *Hadrosauropodus* is bilobed. We support Lockley et al. [18] and do not accept the synonymy proposed by Lucas et al. [16].

Several trackways of *Caririchnium* are quadrupedal. Lockley [76] considered that the shape and position of the manus tracks are diagnostic features. Subsequently, Lim et al. [80] accepted only the manus track shape as diagnostic, because the position of the manus impressions in all quadrupedal ornithopod trackways is variable [21]. This proposal is considered valid here, but further studies on the variability of the manus track shape in relation to the trackmaker and the action of the mud are needed. Assignment to an ichnospecies should be possible even without manus tracks, so it is here postulated that pes tracks should be given greater ichnotaxonomic importance.

In the present study, we propose that only two of the ichnospecies currently assigned to *Caririchnium* can in fact be so assigned: *C*. *magnificum* and *C*. *lotus*. The ichnotaxa *C*. *leonardii* and *C*. *kyoungsookimi* are referred to *Hadrosauropodus*, whereas *C*. *protohadrosaurichnos* is regarded as a *nomen dubium*. “*Ambydactylus*” *kortmeyeri* and *Iguanodontipus billsarjeanti* are here assigned to *Caririchnium* on the basis of the digit and heel shape.

Other large ornithopod tracks share the diagnostic characters of *Caririchnium*, but they cannot be related accurately with a specific ichnospecies. This is the case for many tracks classified as iguanodontid footprints, ornithopod footprints, *Hadrosaurichnoides*, *Brachyguanodonipus* and *Iguanodonipus* from the lowermost part of the Early Cretaceous of Spain, especially from the Urbión [147] and Enciso Groups (basal Valanginian-middle Albian, Spain) (see [126], p.126, table 9.4). Moreover, tracks found in the Areniscas de Camarillas Formation from the Barremian of Spain [131], the Salema Formation from the Hauterivian-Barremian of Portugal [130], and the Wessex Formation from the Barremian of England [145] can also be assigned to *Caririchnium*. The presence of *Caririchnium* in Europe was first cited recently by Díaz-Martínez [126] and has been subsequently supported by Lockwood et al. [145]. Several large ornithopod ichnotaxa considered not valid in the present study display a similar morphology to *Caririchnium*, but they need to be revised: *Sousaichnium*, *Staurichnium*, *Limayichnus* and *Ornithopodichnus*.

***Caririchnium magnificum*** Leonardi, [11]

**Emended diagnosis**

Pes tracks belonging to *Caririchnium* with very large heel pad impressions, approximately as wide as or wider than long; blunt distal end of digit impressions; subtriangular distal part of the heel pad; manus tracks elliptic and wider than long.

**Holotype**

Trackway about 25 m long. Cast of the first manus and pes impressions in the Museu Câmara Cascudo Federal University of Rio Grande do Norte, Natal, Brazil [11] p. 177, fig. 8(Fig. 7A-B).

**Type horizon**

Rio do Peixe Group, Antenor Navarro Formation. Lower Cretaceous [11], Berriasian-Hauterivian, Brazil [146].

**Type locality**

Serrote do Pimenta village, near Sousa, Brazil [11].

**Distribution**

Antenor Navarro Formation, Berriasian-Hauterivian, Brazil (*sensu* [141]); Enciso Group, basal Barremian-middle Albian, Spain (*sensu* [124]).

**Synonymy**

1971 *Iguanodon* footprints, [39], fig.6.

1984 *Caririchnium magnificum*, [11], ichnogen and ichnosp. nov., fig. 8.

1989 *Caririchnium* [137], fig. 31.2 pro parte.

1993 Ornithopod footprints, [149], fig. 67 pro parte.

1993 *Brachyguanodonipus prejanensis* nov. ichnosp. [68], fig.8.12.1–2 pro parte.

1994 *Caririchnium magnificum* [150], plate XXVII, figs. 2, 7.

2001 *Caririchnium magnificum*, [21], fig. 29.3A.

2003 Ornithopod tracks, [151], fig. 20.

2006 Ornithopod footprints, [152], fig. 2.

2009 *Caririchnium magnificum* [146], fig. 7.15.

2013 *Caririchnium* [153], fig. 8.

2013 *Caririchnium* [22], fig. 11H.

2014 *Caririchnium magnificum* [18], fig. 2C.

2014 *Caririchnium magnificum* [145], fig. 12C.

**Referred material**

Several trackways from Brazil and Spain. Material includes a quadruped trackway from the Antenor Navarro Formation in Brazil [11] p.177, fig. 8, and four biped trackways from the Enciso Group in Spain: Cuesta de Andorra tracksite [151], p.184, fig. 20; Malvaciervo tracksite [149] fig. 67, Rastrillada 151; La Magdalena tracksite [68] p. 263, fig. 8.12.1; and Totico 1 tracksite [152] p. 119, fig. 2 1TT3.

**Description**

All the information on the type series is in Leonardi [11].

**Comments**

Leonardi [11] defined *C*. *magnificum* on the basis of a quadruped trackway from the Early Cretaceous of Brazil. He figured as holotypes a pair of manus and pes impressions (Fig. 7A). The pes track belongs to the first pair and the manus track to the third pair. The author proposed in the ichnospecific diagnosis that the heel contour is rounded. Moreover, the pes tracks present a wide heel impression. Therefore, according to the emended diagnosis its presence in *Caririchnium* is justified. The pes tracks of the type trackway have a variable morphology, but most of them show heel pad impressions that are rounded and wider than long (Fig. 7B). A wider than long heel is also present in *C*. *kortmeyeri* and *C*. *billsarjeanti*, though not in *C*. *lotus*, which has longer than wide heel pad impressions. The presence of blunted claw marks is also shared with *C*. *billsarjeanti* and *C*. *lotus*, whereas *C*. *kortmeyeri* presents pointed claw marks. The distal part of the heel pad impression is subtriangular in *C*. *magnificum*, *C*. *kortmeyeri* and *C*. *lotus*, but rounded in *C*. *billsarjeanti*. The manus track shape of *C*. *magnificum* is variable, but generally it is elliptic with the lateromedial axis larger than the posteromedial one.

The ichnospecies *C*. *magnificum* was originally described in the Antenor Navarro Formation of Brazil [11], which is Berriasian-Barremian [154] or Berriasian-Hauterivian in age [146]. In the present work, we have assigned to this ichnospecies four trackways (including part of the material classified as *Brachyguanodonipus* by [68]) from the Enciso Group of La Rioja in Spain (see discussion in [126]). The age of these tracks is basal Berriasian to middle Albian (*sensu* [124]).

***Caririchnium kortmeyeri*** (Currie and Sarjeant, [57]) comb. nov.

**Emended diagnosis**

Pes tracks belonging to *Caririchnium* with very large heel pad impressions, approximately as wide as or wider than long; sharp distal end of digit impressions; subtriangular distal part of the heel pad.

**Holotype**

PMA P76.11.11, natural cast of a track (Currie and Sarjeant, [57], p. 106, fig. 2) (Fig. 7C). Paratypes: BC719, BC720 natural cast, PMA P77.17.6 natural casts [57] p. 107, fig. 3; p. 108, fig. 4; p. 110, fig. 5; p 109, fig. 6 (Fig. 7D).

**Type horizon**

Gething Formation, Bullhead Member; Lower Cretaceous [57], Aptian-Albian, Canada (*sensu* [91]).

**Type locality**

Peace River Canyon, British Columbia, Canada [57].

**Distribution**

Gething Formation, Aptian-Albian, Canada (*sensu* [91]).

**Synonymy**

1979 *Amblydactylus kortmeyeri* [57], ichnosp. nov., figs. 2–6.

1989 *Amblydactylus* [137], fig. 31.2 pro parte.

1990 *Amblydactylus kortmeyeri* [19], fig. 6.37l.

2008 *Amblydactylus kortmeyeri* [60], fig. 5C.

2011 *Amblydactylus kortmeyeri* [155], fig. 12.3H.

2014 *Amblydactylus kortmeyeri* [18], fig. 2B.

2014 *Amblydactylus kortmeyeri* [145], fig. 12B.

**Referred material**

Several tracks from the Gething Formation in Canada [57] p. 106, fig. 2, PMA P76.11.11; p. 107, fig. 3, BC719; p. 108, fig. 4, PMA P76.17.6a.

**Description**

All the information on the type series is in Currie and Sarjeant [57].

**Comments**

Currie and Sarjeant [57] defined this ichnotaxon on the basis of a well-preserved natural cast (holotype) and several tracks and trackways (paratypes) found in the Aptian-Albian of western Canada (Fig. 7C-D). They suggested that the main differences between *A*. *gethingi* and *A*. *kortmeyeri* are the length/width ratio of the track (greater in *A*. *gethingi*), its contour shape, and the distal end of the digits (more tapered in *A*. *gethingi*). Currie and Sarjeant [57] suggested that these differences may depend on the circumstances of track formation. Currie [41] noted that it was possible to distinguish the ichnospecies in well-preserved tracks, but this identification was not possible for the majority of the *Amblydactylus* tracks. Currie [41, 43] considered that *A*. *gethingi* and *A*. *kortmeyeri* seem to represent the same general type of animal, and discussed both ichnospecies together. Gangloff et al. [61] suggested that *A*. *gethingi* could be a senior synonym of *A*. *kortmeyeri*. In the present work, the type ichnospecies of *Amblydactylus*, *A*. *gethingi*, is provisionally considered a *nomen dubium* (see above). Therefore, *A*. *kortmeyeri* could be assigned to another ichnogenus, used to define a new ichnogenus, or become the type ichnospecies of *Amblydactylus* after a formal request to the ICZN. This ichnospecies is mainly characterized by a wide and rounded heel impression, as is typical in *Caririchnium*. Consequently, we propose to assign *A*. *kortmeyeri* to *Caririchnium*.

There are no manus tracks in the type series of *C*. *kortmeyeri*. Currie and Sarjeant [57] assigned some trackways as paratypes, but the track drawings are somewhat different in form from the holotype. The holotype specimen has as its only autapomorphy the sharp claw impressions that are different from other ichnospecies of *Caririchnium*. Further studies should discuss whether this character is of ichnotaxonomic significance or is due to preservational biases. *C*. *kortmeyeri* also differs from *C*. *lotus* in the width of the heel pad impression, from *C*. *billsarjeanti* in the shape of the distal part of the heel pad, and from *C*. *magnificum* only in the shape of the claw impressions.

***Caririchnium billsarjeanti*** (Meyer and Thüring, [105]) comb. nov.

**Emended diagnosis**

Pes tracks belonging to *Caririchnium* with very large heel pad impressions, approximately as wide as or wider than long; blunt distal end of digit impressions; rounded distal part of the heel pad; manus tracks elliptic and wider than long.

**Holotype**

NMB K.S. 374, cast of T1 trackway segment [105] p. 225, fig. 6 (Fig. 7 E–F).

**Type horizon**

Upper part of Schrattenkalk Formation, Lower Cretaceous, lower-middle Aptian to middle-upper Aptian [105].

**Type locality**

Risleten quarry, Switzerland [105].

**Distribution**

Schrattenkalk Formation, Aptian [105], Switzerland.

**Synonymy**

2003 *Iguanodontipus billsarjeanti* [105], ichnosp. nov. figs. 4–6, 8-9.

2012 *Iguanodontipus billsarjeanti* [25], fig. 1B.

2013 *Iguanodontipus* [22], fig. 11F.

2013 *Iguanodontipus billsarjeanti* [129], fig. 13E.

**Referred material**

Three trackways (TR1, TR2 and TR3) from the Schrattenkalk Formation in Switzerland [105] p. 223, figs. 4, 5; p. 225, fig. 6; p. 226, figs. 8, 9.

**Description**

All the information on the type series is in Meyer and Thüring [105].

**Comments**

This ichnotaxon was defined on the basis of a well-preserved quadruped trackway from the Aptian of Switzerland (Fig. 7F). The pes tracks have one pad impression in each digit and one in the heel, and the manus tracks are small and elliptic [105] (Fig. 7E). The ichnospecies, which was originally referred to *Iguanodontipus*, is regarded as valid (see above). However, the observed features do not correspond with those of *Iguanodontipus* but with *Caririchnium*. Therefore, the ichnospecies *billsarjeanti* is placed in *Caririchnium* as *C*. *billsarjeanti* nov. comb.

The general shape of the holotype is very similar to that of *C*. *magnificum* and *C*. *kortmeyeri*. Moreover, the manus track is also similar to that of *C*. *magnificum*. Nevertheless, *C*. *billsarjeanti* differs from other ichnospecies of *Caririchnium* in that the distal part of the heel pad is rounded (instead of subtriangular). Future studies might resolve whether this character is part of the variability in the preservation of *C*. *magnificum* or *C*. *kortmeyeri*.

***Caririchnium lotus*** Xing, Wang, Pan and Chen, [81]

**Emended diagnosis**

Pes tracks belonging to *Caririchnium* with very large heel pad impressions that are longer than wide; blunt distal end of digit impressions; subtriangular distal part of heel pad; manus tracks rectangular, smoothly concave in the proximal part and wider than long.

**Holotype**

QJGM-T37–3 [81] p. 1597, fig. 5 (Fig. 7G).

**Type horizon**

Jiaguan Formation; Lower Cretaceous, Barremian-Albian (*sensu* [148]).

**Type locality**

Qijiang, Chongqing, China [81].

**Distribution**

Jiaguan Formation, Barremian-Albian, China (*sensu* [148]); Enciso Group, basal Barremian-middle Albian, Spain (*sensu* [124]).

**Synonymy**

1984 *Iguanodon* footprints [156], photographs 9–13.

1989 Iguanodontid footprints [157], fig. 12 pro parte.

1993 *Iguanodonipus cuadrupedae* [68], p. 394.

1995 Iguanodontid footprints [158], p. 58.

1997 Ornithopod footprints [159], fig. 2.

2003 Ornithopod tracks [151], fig. 19.

2006–07 Ornithopod footprints [160], fig. 2.

2007 *Caririchnium lotus* [81], ichnosp. nov., figs. 3, 5.

2012 *Caririchnium lotus* [161], figs. 3–4.

2014 *Caririchnium lotus* [18], fig. 2E.

**Referred material**

Several trackways from China and Spain. Material includes tracks from the Jiaguan Formation of China: QJGM-T37–3 [79] p. 1597, fig. 5, QJGM-T100–1 [161] p. 306, fig. 3A; p. 307, fig. 4; and trackways from the Enciso Group of Spain: La Canal tracksite [151] p.183, fig. 19, Barranco de Valdecevillo tracksite [157] fig. 12, Barranco de Valdegutiérrez tracksite [152] fig. 2, 1BVG1, 1BVG4, and Era del Peladillo 5 [158], fig. 2.

**Description**

All the information on the type series is in Xing et al. [81] and Xing et al. [161].

**Comments**

*C*. *lotus* was defined by Xing et al. [81] on the basis of biped and quadruped trackways from the “mid”-Cretaceous of China. The material consists of about 200 well-preserved tracks of different sizes. Xing et al. [81] classified the pes tracks in three groups according to their length: 37–40 cm (adults); 25–30 cm (subadults); and 19–23 cm (juveniles). This ichnotaxon has large, rounded heel impressions [81, 161] (Fig. 7G-H), so its assignment to *Caririchnium* is justified. *C*. *lotus* is mainly characterized by having a heel pad impression that is longer than wide, in contrast to other ichnospecies of *Caririchnium*, which are wider than long. Xing et al. [81] suggested that the manus tracks had digit impressions in their distal part (Fig. 7I). Subsequently, Xing et al. [161] stated that the manus track is rectangular in shape, with rounded edges and a slightly concave proximal surface. This feature of *C*. *lotus* is also different from the other ichnospecies of *Caririchnium*.

The ichnospecies *C*. *lotus* was originally described in the Jiaguan Formation in China which is Barremian-Albian in age, (*sensu* [148]). In the present work, we have assigned to this ichnospecies tracks of four tracksites (including part of the material classified as *Iguanodonipus* by [68]) from the Enciso Group of La Rioja in Spain (see discussion in [126]). The age of these tracks is basal Barremiean to middle Albian (*sensu* [124]).

Ichnogenus ***Hadrosauropodus*** Lockley, Nadon and Currie, [24]

**Emended diagnosis**

Tracks belonging to Iguanodontipodidae with a large heel impression that is bilobed, centred and wide (wider than the width of the proximal part of the digit III impression); pad of digit III shorter than those of digits II and IV; short, wide digit impressions with blunt distal ends.

**Type ichnospecies**

*Hadrosauropodus langstoni* Lockley, Nadon and Currie, [24]

**Other ichnospecies**

*Hadrosauropodus leonardii* (Lockley, [76]); *Hadrosauropodus kyoungsookimi* (Lim, Lockley and Kong, [80]).

**Distribution (ichnospecies and referred material)**

Gyeongsang Group, Aptian-Albian, Korea [162]; Gething Formation, Aptian-Albian, Canada [81]; Jindong Formation, upper Aptian, Korea [80]; Pajarito Formation, upper Albian, USA [79]; Dakota Group, Albian-Cenomanian, USA [163]; Mojado Formation, Albian-Cenomanian, USA and Mexico [164]; Menefee Formation, Campanian, USA [26]; Mesa Verde Group, Campanian, USA [165]; Cantwell Formation, late Campanian or early Maastrichtian, USA [166]; Wapiti Formation, late Campanian-early Maastrichtian, Canada [167]; Lance Formation, Maastrichtian, USA [24]; St. Mary River Formation, Maastrichtian, Canada [24]; Zhutian Formation, Maastrichtian, China [99]; Tremp Formation, Maastrichtian, Spain [168].

**Comments**

*Hadrosauropodus* was defined by Lockley et al. [24] on the basis of tracks from the Maastrichtian of Canada previously regarded as “hadrosaur footprints” [42]. These footprints are mainly characterized by a bilobed heel impression and short, wide digits. As noted above, these features allow *Hadrosauropodus* to be distinguished from *Caririchnium* and *Iguanodontipus*.

To date, there are only a few citations of *Hadrosauropodus*. In the original paper, Lockley et al. [24] proposed as the type series several footprints from the St. Mary River Formation (Maastrichtian, Canada), and assigned to *Hadrosauropodus* isp. footprints from the Lance Formation (Maastrichtian, USA). In a preliminary study, Suñer et al. [169] assigned some casts from the Maastrichtian of Spain to *Hadrosauropodus langstoni*. Xing et al. [99] described *Hadrosauropodus nanxiongensis* from several tracks found in the Zhutian Formation (Maastrichtian, China), and assigned to *Hadrosauropodus* isp. additional tracks from the same formation. As discussed above, *H*. *nanxiongensis* is considered to be a *nomen dubium*, but the presence of a bilobed heel impression suggests that the tracks belong to *Hadrosauropodus*. Recently, Vila et al. [168] classified as *Hadrosauropodus* isp. several footprints found in the late Maastrichtian of the southern Pyrenees in Spain. All these data suggest that the ichnogenus *Hadrosauropodus* could present a wide geographical distribution (North America, Asia and Europe), but a very limited temporal distribution (Maastrichtian). Nevertheless, *Hadrosauropodus* is not the first ichnotaxon to which footprints with bilobed heel impressions have been assigned. Lockley [77] classified one footprint with skin and a bilobed heel impression from the Dakota Formation (Albian-Cenomanian) as *Caririchnium*. Since then, all the footprints with this kind of heel impression have been assigned to *Caririchnium*, except the Maastrichtian ones related to *Hadrosauropodus*. Examples include the ichnospecies *C*. *leonardii* and *C*. *kyoungsookimi* from the mid-Cretaceous of the USA and Korea, respectively (see [21, 80, 170]). Based on this, Lucas et al. [16] suggested that *Hadrosauropodus* is a junior synonym of *Caririchnium*. In the present paper, we consider that *Caririchnium* has a rounded and not bilobed heel impression, and is thus different from *Hadrosauropodus*. Consequently, *C*. *leonardii* and *C*. *kyoungsookimi* are referred to *Hadrosauropodus*.

Currie [43] assigned to *Amblydactylus* isp. several quadrupedal tracks from the Aptian-Albian of Canada with bilobed heel impressions. Moreover, Carpenter [165] described bilobed tracks from the Campanian of the Mesa Verde Group (USA) and interpreted them as hadrosaur footprints. Pending revision, these tracks are here provisionally regarded as belonging to *Hadrosauropodus*.

***Hadrosauropodus langstoni*** Lockley, Nadon and Currie, [24]

**Emended diagnosis**

Pes tracks belonging to *Hadrosauropodus* with a heel impression much wider than the width of the proximal part of the digit III impression; proximal part of the pads of digits II and IV situated close to the proximal part of the heel pad; manus tracks are obtuse-isosceles-triangle-shaped.

**Holotype**

TMP 87.76.7 [24] 240, fig. 12A (Fig. 8A).

**Type horizon**

St. Mary River Formation, Upper Cretaceous, Maastrichtian [24].

**Type locality**

St. Mary River Valley, about 20 km S-SW of Lethbridge, Alberta, Canada [24].

**Distribution**

St. Mary River Formation, Maastrichtian, Canada [24].

**Synonymy**

1991 Hadrosaur tracks [42], figs. 5–6.

1991 Hadrosaur tracks [138] fig. 5.3.

2001 Hadrosaur tracks [21], fig. 29.4D.

2001 Dinosaur track [171] fig. 27.4.

2003b *Hadrosauropodus langstoni* [24], ichnogen. and ichnosp. nov., figs. 11–12.

2008 *Hadrosauropodus langstoni* [172], fig. 7D.

2008 *Hadrosauropodus langstoni* [60], fig. 5D.

2011 *Hadrosauropodus langstoni* [16], fig. 5, pro parte.

2013 *Hadrosauropodus langstoni* [168], fig. 7Q.

2014 *Hadrosauropodus langstoni* [18], fig. 4D.

**Referred material**

Tracks from the St. Mary River Formation, Canada ([24] p. 243–244, figs. 11 right, 12).

**Description**

All the information on the type series is in Lockley et al. [24].

**Comments**

Lockley et al. [24] defined this ichnotaxon on the basis of several tracks from the Maastrichtian of Canada (Fig. 8A-B). *H*. *langstoni* is mainly characterized by having pes tracks that are much larger than the manus tracks, one pad impression in each digit and one in the heel, which is bilobed. Before the study by Lockley et al. [24], these tracks had been assigned to hadrosaurs (e.g., [21, 43, 138]). The tracks are very well preserved and have manus prints, skin impressions and tail marks. This ichnospecies is characterized above all by a very wide heel impression. Moreover, the notches of digits II and IV are positioned far back, close to the proximal part of the heel. Other ichnospecies of *Hadrosauropodus* show a narrower heel and the notches of digits II and IV are close to the proximal part of the digit III pad impression. On the other hand, the manus tracks of *H*. *langstoni* are different (triangular) from those of *H*. *leonardii* (rectangular) and *H*. *kyoungsookimi* (crescent-shaped).

***Hadrosauropodus leonardii*** (Lockley, [76]) comb. nov.

**Emended diagnosis**

Pes tracks belonging to *Hadrosauropodus* with a heel impression as wide as or slightly wider than the width of the proximal part of the digit III impression; proximal part of the pads of digits II and IV situated in the medial-distal part of the heel pad; manus tracks are ovoid to rectangular, with the digit I impression in the proximal part and directed medially.

**Holotype**

Trackway A [76] p. 108, fig. 2; p. 111, fig. 4e; p. 114 (Fig. 8C).

**Type horizon**

South Platte Formation, Dakota Group, Albian-Cenomanian [76].

**Type locality**

Dinosaur Ridge (Alameda Parkway), Jefferson Country, Colorado, USA.

**Distribution**

Dakota Group, “mid”-Cretaceous, Albian-Cenomanian, USA (*sensu* [163]).

**Synonymy**

1987 *Caririchnium leonardii* [76], ichnogen. and ichnosp. nov., fig. 5A

1988 *Caririchnium* [77], fig. 4a.

1988 *Caririchnium* [77], fig. 6.

1989 *Caririchnium* [106], fig. 3D.

1989 *Caririchnium* [137], fig. 31.2 pro parte.

1990 *Caririchnium leonardii* [19], fig. 6.32d.

1991 *Caririchnium leonardii* [42], fig. 4.

2001 *Caririchnium leonardii* [21], fig. 29.4c.

2011 *Caririchnium* [16], fig. 5 pro parte.

2014 *Caririchnium leonardii* [18], fig. 2D.

**Referred material**

Two trackways from the Dakota Group of Colorado, USA ([76] p. 114, fig. 5A; [42], p. 109, fig. 4).

**Description**

All the information on the type series is in Lockley [76].

**Comments**

Lockley [76] defined the ichnospecies *C*. *leonardii* on the basis of a trackway from the “mid”-Cretaceous of the USA. He noted that the main difference relative to *C*. *magnificum* is the shape of the manus tracks. Several researchers (e.g., [80, 173–174]) have assigned footprints with a bilobed heel to *Caririchnium leonardii*. In the original paper, Lockley [76] described a trackway in which the footprints have a poorly-preserved heel impression (shown by a dashed line or unclosed contour line) (Fig. 8C) and the heel shape is not mentioned. Lockley [77] (fig. 6) assigned to *Caririchnium* a bilobed footprint. This footprint was figured by Lucas et al. [16] as having a *Caririchnium*-like morphology.

The bilobed heel has been used as a diagnostic feature of *Caririchnium* (e.g., [95, 170, 173) and *Caririchnium leonardii* (e.g., [42, 175]). In the present work, we have emended the diagnosis of *Hadrosauropodus*, and we consider it to be the only ichnogenus with a bilobed heel impression. Therefore, *C*. *leonardii* is assigned to *Hadrosauropodus* and not to *Caririchnium*.

As occurs with *Iguanodontipus*, several authors (e.g., [16, 21]) have used the referred footprints of *H*. *leonardii* as a model for comparison instead of those that form part of the type series (Fig. 8B-C). One example is a well-preserved quadrupedal trackway from the Dakota Group of Colorado described by Lockley [77] (fig. 6) and subsequently by Currie et al. [42]. This trackway consists of eight pes tracks with skin impressions and several well-preserved manus tracks. Taking into consideration that the outline of the footprints from the type series of *C*. *leonardii* is unreliable (see [76–77, 106, 176]), in the present paper the diagnosis has been completed with data from the second trackway (Fig. 8D-E).

The footprints of *H*. *leonardii* are characterized primarily by having a narrower heel than *H*. *langstoni*, with the notches of digits II and IV placed more distally. Nevertheless, there are no clear differences with respect to the pes tracks of *H*. *kyoungsookimi*. The rectangular manus tracks of *H*. *leonardii* are different from those of *H*. *langstoni* and *H*. *kyoungsookimi* (see above).

***Hadrosauropodus kyoungsookimi*** (Lim, Lockley and Kong, [80]) comb. nov.

**Emended diagnosis**

Pes tracks belonging to *Hadrosauropodus* with a heel impression as wide as or slightly wider than the width of the proximal part of the digit III impression; proximal part of the pads of digits II and IV situated in the medial-distal part of heel pad; manus tracks are crescent in outline, with three circular digit impressions of about the same size.

**Holotype**

NHCG 10194, partial trackway with two successive manus-pes sets [80] p. 111, figs. 2–3 (Fig. 8F).

**Type horizon**

Jindong Formation, Lower Cretaceous, upper Aptian [80].

**Type locality**

Duhori area, Goseong County, Korea [80].

**Distribution**

Jindong Formation, Aptian, Korea [80].

**Synonymy**

*Caririchnium kyoungsookimi* Lim, Lockley and Kong, [80]

**Referred material**

Tracks from the Jindong Formation of Korea [80] p. 103, figs. 2–3, NHCG 10194.

**Description**

All the information on the type series is in Lim et al. [80].

**Comments**

Lim et al. [80] defined this ichnospecies on the basis of two pairs of manus-pes tracks preserved in a block of stone from the Aptian of Korea (Fig. 8F). They compared *C*. *kyoungsookimi* with ichnospecies of *Caririchnium*, *Iguanodontipus* and *Amblydactylus*, and suggested differences in the shape of the manus tracks. However, Lim et al. [80] did not compare the material with *Hadrosauropodus*, with which it shares a bilobed heel impression. In the present work, we propose to assign *C*. *kyoungsookimi* to *Hadrosauropodus*.

The bilobed heel impression of *H*. *kyoungsookimi* is similar to that of *H*. *leonardii*, and narrower than that of *H*. *langstoni*. Bearing in mind the view propounded by Lockley [76] and Lim et al. [80] that the manus track shape is a diagnostic feature, *H*. *kyoungsookimi* is different from the other ichnospecies. Nevertheless, further studies should analyse the variability in the manus track impressions observed in the quadruped trackways of *H*. *leonardii* in order to discuss possible synonyms.

### Geographical and temporal distribution

The ichnofamily Iguanodontipodidae is mainly related to tracks found in the Cretaceous—from the Berriasian to the Maastrichtian—of Europe, Asia, North America and South America (Fig. 9). This distribution could yet be extended in time, since large ornithopod tracks have been described from the Late Jurassic (e.g., [177–180]), or geographically, if we take into consideration some tracks found in Australia [12] and Africa (Cameroon [181]; Morocco [13–14]). We consider that these tracks could have ornithopod affinities but do not show diagnostic characters of the ichnofamily Iguanodontipodidae.

Iguanodontipodid tracks are present in all the Cretaceous stages, but there is a stratigraphic hiatus spanning from the Turonian to the Coniacian (Fig. 9), which may be correlated with transgressive sea-level phases [182]. This hiatus appears to be more marked in Europe than in North America and South America, with the apparent absence of tracks and trackways in the Cenomanian and even in the Santonian if *Apulosauripus* is considered to be a non-ornithopod ichnotaxon (see above).

The geographical and temporal distribution of *Iguanodontipus* is very limited. All the tracks referred to *I*. *burreyi* come from the earliest Cretaceous (Berriasian-Valanginian) of Europe, including England, Germany and Spain (Fig. 10).

The ichnogenus *Caririchnium* has a wider distribution, both temporally and geographically, than *Iguanodontipus*. *Caririchnium* has been identified in the Early Cretaceous of South America, North America, Asia and Europe (Fig. 10). The type ichnospecies *C*. *magnificum* is known from the Berriasian-Hauterivian of Brazil to the Barremian-Albian of Spain (Fig. 10B). *C*. *lotus* also has an intercontinental distribution: this ichnospecies is known in the Barremian-Albian of China and Spain (Fig. 10B). As regards *C*. *kortmeyeri* (previously referred to *Amblydactylus*), this has only been cited in the Aptian-Albian of Canada (Fig. 10B). Finally, *C*. *billsarjeanti* (originally assigned to *Iguanodontipus*) is known exclusively from the Aptian-Albian of Switzerland (Fig. 10B).

Tracks of *Hadrosauropodus* have been identified in Asia, North America and Europe (Fig. 10). The oldest records (previously referred to *Caririchnium*) are those of *H*. *kyoungsookimi* from the Aptian-Albian of South Korea and *H*. *leonardii* from the Albian-Cenomanian of the United States. *H*. *langstoni* has only been cited in its type locality from the Maastrichtian of Canada, but *Hadrosauropodus* tracks have been identified in latest Cretaceous formations in Europe and Asia (Fig. 10B).

The data presented here show the temporal and geographical trends of the ichnotaxa in question. This analysis is based on the large ornithopod tracks previously assigned to a particular ichnogenus or ichnospecies (S1 Table). Tracks classified informally as “large ornithopod footprints” or referred to indeterminate iguanodonts and hadrosaurs will be studied in detail in a further paper with the aim of ascertaining the precise temporal and geographical distribution of Iguanodontipodidae.

### Possible identity of trackmakers

Ornithopoda is a taxon defined as all ornithischians more closely related to *Edmontosaurus* than to *Triceratops* [183]. Ornithopoda (“bird feet”) was the name used by Marsh [184] to designate bipedal, unarmoured herbivorous dinosaurs. It has been used for a long time as a taxonomic wastebasket, into which almost all bipedal ornithischians have been placed ([185–186], and references). Recent phylogenetic studies have restricted Ornithopoda to a clade that includes a paraphyletic assemblage of “hypsilophodontids” and iguanodontians comprising tenontosaurs, rhabdodontids, dryosaurids, “camptosaurids”, “iguanodontids” and hadrosauroids. Ornithopods are known in the fossil skeletal record from the Middle Jurassic (e.g., the earliest dryosaurid *Callovosaurus*; see [187]) to the end of the Cretaceous.

Large ornithopods consist mostly of iguanodontian forms, with the exception of dryosaurids (2–4 m in length) and rhabdodontids (up to 5 m length). Well-preserved skeletal material of iguanodontians has been recorded from the Late Jurassic to the Late Cretaceous in fossiliferous sites in all continents [188]. By the Early Cretaceous, large iguanodontians were widely distributed, being present in Europe, North America, Asia, Africa and Australia. Hadrosauroids reached a near-cosmopolitan distribution during the Late Cretaceous, with records in all landmasses except Africa, Australia and India.

Based on their size and morphology, the tracks assigned to Iguanodontipodidae would correspond to iguanodontian ichnites. The apparent absence of iguanodontipodid tracks in the Late Jurassic could be an artefact due to taphonomic or ecological biases. Moreover, it cannot be ruled out that Late Jurassic iguanodontians produced different tracks from the Cretaceous ones [189].

The ichnotaxon *Iguanodontipus burreyi* was proposed by Sarjeant et al. [23] to accommodate “*Iguanodon* footprints” and those of typical “iguanodontids” (commonly regarded as a paraphyletic assemblage; [190] and references; but see [191] for a different interpretation). Tracks of *Iguanodontipus burreyi* are limited to the basal Cretaceous (Berriasian-Valanginian) of Europe (Fig. 10B). Consequently, the trackmaker cannot be *Iguanodon*, because *I*. *bernissartensis*—the only currently recognized species of the genus—is known from the Barremian-early Aptian of England and Belgium (see [192] for a revised taxonomy of Wealden iguanodontians and references). *Iguanodontipus* tracks might be associated with basal members of Ankylopollexia or Styracosterna from the Berriasian-Valanginian of Europe (e.g., *Barilium dawsoni* and *Hypselospinus fittoni*, both from the Valanginian Lower Wealden Group of England, see [193]; or *Owenodon hoggii* from the Berriasian Purbeck Beds of England; see [194–195]), but these ichnites cannot be reliably assigned to particular taxa in the absence of detailed knowledge of the foot anatomy of these iguanodontian ornithopods [196].

*Caririchnium*, and particularly the type ichnospecies *C*. *magnificum*, spans all the Early Cretaceous: the oldest records correspond to the basal Cretaceous of South America; other ichnospecies of *Caririchnium* are known in the Barremian-Aptian of Europe, North America and Asia (Fig. 10). The *C*. *magnificum* trackway in Brazil could have been made by a basal iguanodontian, perhaps a basal ankylopollexian or styracosternan (although there is no skeletal record of large representatives of these clades in the Early Cretaceous of South America; [146, 197]). The same interpretation can be applied to the other ichnospecies of *Caririchnium*, namely *C*. *billsarjeanti*, *C*. *kortmeyeri* and *C*. *lotus*. Large-sized representatives of basal styracosternans and “iguanodontids” are known in the late Early Cretaceous of North America (*Hippodraco*, *Iguanacolossus*, *Theiophytalia*; see [190, 198], and references), Europe (*Iguanodon*, *Mantellisaurus*; see [192, 199]) and, tentatively, Asia (*Lanzhousaurus*; [200]). Iguanodontids (*sensu* [191]) are also known in the late Early Cretaceous of Africa (*Ouranosaurus*), though there is still no evidence of *Caririchnium* or large ornithopod tracks in this part of Gondwanaland.

The temporal distribution of the ichnogenus *Hadrosauropodus* (Aptian-Maastrichtian) is quite coherent with the skeletal fossil record of Hadrosauroidea. Hadrosauroidea consists of all taxa more closely related to *Edmontosaurus* than to *Iguanodon* [186], and includes the hadrosaurids, which became the most diverse and abundant large vertebrates of Laurasia during the second half of the Late Cretaceous [201]. The earliest hadrosauroids are known from the late Early Cretaceous, and were particularly diversified in Asia [202] and to a lesser extent in North America ([186], and references). On stratigraphical grounds, the tracks of *Hadrosauropodus kyoungsookimi* from the Barremian-Albian of Asia and *H*. *leonardii* from the Albian-Cenomanian of North America could have been made by non-hadrosaurid hadrosauroids (such as *Altirhinus*, *Bolong*, *Equijubus*, *Jinzhousaurus*, *Probactrosaurus* and *Xuwulong* from the Barremian-Albian of Asia, and *Eolambia* and *Protohadros* from the Cenomanian of North America), whereas those of *H*. *langstoni* from the Maastrichtian of North America and *Hadrosauropodus* isp. from the Maastrichtian of Europe could have been made by derived hadrosaurids. However, the data currently available do not permit confident identification of the trackmakers on the basis of morphological features.

## Conclusions

The ichnotaxonomy of large ornithopod tracks has been revised: of 44 ichnospecies described in the literature only eight are here considered to be valid. These ichnospecies are grouped into three ichnogenera: *Iguanodontipus*, *Caririchnium* and *Hadrosauropodus*, mainly on the basis of the size and shape of the heel and digit pad impressions. The manus track shape is of ichnotaxonomic value, but the diagnosis of large ornithopod ichnotaxa should be primarily based on the features of the pes tracks. The monospecific ichnogenus *Iguanodontipus* is mainly characterized by a narrow, rounded heel, and long, thin digit impressions. The distribution of *I*. *burreyi* is limited to the basal Cretaceous (Berriasian-Valanginian) of Europe. *Caririchnium* is characterized by a large, rounded heel, and short digit impressions. Four ichnospecies of *Caririchnium* have been recognized in this work: *C*. *magnificum* (type ichnospecies), *C*. *lotus*, *C*. *kortmeyeri* (formerly referred to *Amblydactylus*) and *C*. *billsarjeanti* (previously referred to *Iguanodontipus*). *Caririchnium* is known in the Early Cretaceous (Berriasian-Albian) of South America, North America, Asia and Europe. *Hadrosauropodus* is characterized by a wide, bilobed heel, and short, wide digit impressions. *Hadrosauropodus* consists of the type ichnospecies *H*. *langstoni* and two ichnospecies that were formerly assigned to *Caririchnium*: *H*. *leonardii* and *H*. *kyoungsookimi*. *Hadrosauropodus* ranges from the Aptian to the Maastrichtian, and is known in North America, Asia and Europe.

*Iguanodontipus*, *Caririchnium* and *Hadrosauropodus* are included within the ichnofamily Iguanodontipodidae. This ichnofamily is characterized mainly by mesaxonic, tridactyl, subsymmetrical pes tracks that are as wide as (or wider than) long and have one pad impression in each digit and one in the heel. Tracks belonging to Iguanodontipodidae can be assigned to iguanodontian ornithopods: those of *Iguanodontipus* and *Caririchnium* could have been made by basal representatives of Ankylopollexia or Styracosterna, whereas those of *Hadrosauropodus* could have been made by hadrosauroids. Iguanodontipodid tracks can confidently be said to be distributed in the Cretaceous of Europe, Asia, North America and South America.

## Supporting Information

S1 Table Citations of large ornithopod ichnogenera studied in this work. * paper in which the ichnotaxon was described.
(DOCX)

S1 Text Data on large ornithopod ichnotaxa (in alphabetical order): diagnosis, holotype, type horizon and type locality. The diagnoses are in the original language.
(DOCX)
