# Supplementary material for: Ichnotaxonomic Review of Large Ornithopod Dinosaur Tracks: Temporal and Geographic Implications
Source: PLoS One. 2015 Feb 12;10(2):e0115477. doi: 10.1371/journal.pone.0115477 (PMC4326173; doi:10.1371/journal.pone.0115477)
Supplement: S1 Table — * paper in which the ichnotaxon was described. (DOCX) [file pone.0115477.s001.docx]

**Table S1: Citations of large ornithopod ichnogenera studied in this work.** * paper in which the ichnotaxon was described.

| **Icnotaxon** | **Reference** | **Country** | **Age** |
| --- | --- | --- | --- |
| *Akmechetosauropus* | [1] * | Tajikistan | Albian |
| *Amblydactylus* | [2] * | Canada | Aptian-Albian [3] |
|  | [4] * | Canada | Aptian-Albian [3] |
|  | [5] | Canada | Aptian-Albian [3] |
|  | [6] | USA | Aptian |
|  | [7] | Canada | Aptian-Albian [3] |
|  | [8] | USA | Aptian |
|  | [9] | USA | Cenomanian |
|  | [10] | USA | Albian |
|  | [11] | USA | Campanian-Maastrichtian |
|  | [12] | Canada | Middle Albian-Cenomanian |
|  | [13] | Mongolia | Cenomanian-Maastrichtian |
|  | [14] | Australia | Upper Albian-Cenomanian |
|  | [15] | England | Barremian |
| *Apulosauripus* | [16] * | Italy | Santonian |
| *Babatagosauropus* | [1] * | Tajikistan | Albian |
| *Bonaparteichnium* | [17] * | Argentina | Albian-Cenomanian? |
| *Brachyguanodonipus* | [18] * | Spain | Basal Barremian-Middle Albian [19] |
|  | [20] | Spain | Basal Barremian-Middle Albian [19] |
| *Camptosaurichnus* | [21] * | Chile | Thitonian [22] |
| *Camptosauropus* | [23] * | Tajikistan | Upper Jurassic [24] |
| *Caririchnium* | [25] * | Brazil | Berriasian-Hauterivian [26] |
|  | [27] * | USA | Albian-Cenomanian |
|  | [28] | USA | Albian-Cenomanian |
|  | [29] | USA | Albian-Cenomanian |
|  | [30] | Korea | Aptian-Albian |
|  | [31] * | USA | Cenomanian |
|  | [9] | USA | Cenomanian |
|  | [8] | USA | Aptian |
|  | [3] | USA | Albian |
|  | [32] | USA | Cenomanian |
|  | [10] | USA | Upper Albian |
|  | [33] | USA | Albian |
|  | [34] | Korea | Santonian-Campanian |
|  | [35] | Brazil | Berriasian-Hauterivian [26] |
|  | [36] | Japan | Valanginian-Albian |
|  | [36] | Korea | Valanginian-Barremian |
|  | [37] | USA | Upper Albian-Cenomanian |
|  | [38] | USA | Santonian-Campanian |
|  | [39] | Korea | Campanian-Maastrichtian |
|  | [40] * | China | “Mid” Cretaceous |
|  | [13] | Mongolia | Cenomanian-Maastrichtian |
|  | [41] | Thailand | Aptian-Albian |
|  | [42] | USA | Albian-Cenomanian |
|  | [43] | USA, Mexico | Upper Albian sup-Lower Cenomanian |
|  | [44] | USA | Albian |
|  | [45] * | Korea | Upper Albian |
|  | [46] | China | Aptian-Santonian |
|  | [47] | Spain | Basal Hauterivian-Middle Albian [19] |
|  | [15] | England | Barremian |
| *Gigantoshiraminesauropus* | [48] * | Japan | Hauterivian-Barremian [30] |
| *Goseongosauripus* | [49] * | Korea | Aptian-Albian [50] |
| *Gypsichnites* | [2] * | Canada | Aptian-Albian [3] |
|  | [3] | Canada | Middle Albian-Lower Cenomanian |
|  | [12] | Canada | Aptian-Albian |
| *Hadrosaurichnoides* | [51] * | Spain | Basal Barremian-Middle Albian [19] |
|  | [52] | Spain | Basal Barremian-Middle Albian [19] |
|  | [53] | Spain | Basal Barremian-Middle Albian [19] |
| *Hadrosaurichnus* | [54] * | Argentina | Maastrichtian |
|  | [55] * | Peru | Campanian-Maastrichtian |
| *Hadrosauripeda* | [56] | Canada | Maastrichtian |
| *Hadrosauropodus* | [57] * | Canada, USA | Maastrichtian |
|  | [58] | Spain | Maastrichtian |
|  | [59] * | China | Maastrichtian |
|  | [60] | Canada | Lower Campanian-Upper Maastrichtian |
|  | [61] | Spain | Maastrichtian |
|  | [62] | USA | Late Campanian-Early Maastrichtian |
|  | [63] | Canada | Late Campanian-Early Maastrichtian |
| *Iguanodonichnus* | [21] * | Chile | Thitonian [22] |
|  | [21] | Chile | Oxfordian |
| *Iguanodonipus* | [18] * | Spain | Basal Hauterivian-Middle Albian [19] |
| *Iguanodonopus* | [64] * | China | Aptian-Albian [65] |
| *Iguanodontipus* | [66] * | England | Berriasian |
|  | [67] | Spain | Kimmeridgian |
|  | [68] * | Switzerland | Lower Aptian-Upper Aptian |
|  | [69] | Germany | Berriasian |
|  | [70] | Germany | Berriasian |
|  | [71] | England | Valanginian-Lower Aptian |
|  | [72] | Germany | Berriasian |
|  | [73] | Spain | Basal Hauterivian-Middle Albian [19] |
|  | [74] | Spain | Berriasian |
|  | [75] | Germany | Kimmeridgiense |
|  | [76] | Spain | Lower Barremian |
|  | [77] | Portugal | Barremian |
|  | [47] | Spain | Berriasian-Valanginian [19] |
| *Jianyinosauropus* | [78] * | China | Albian-Cenomanian [79] |
| *Kharkushosauropus* | [1] * | Tajikistan | Thitonian |
| *Limayichnus* | [17] * | Argentina | Albian-Cenomanian? |
| *Orcauichnites* | [80] * | Spain | Maastrichtian |
| *Ornithopodichnites* | [80] * | Spain | Maastrichtian |
| *Ornithopodichnus* | [81] * | Korea | Albian |
|  | [82] | Korea | Albian |
| *Shiraminesauropus* | [48] * | Japan | Hauterivian-Barremian [30] |
|  | [48] * | Japan | Hauterivian-Barremian [30 |
| *Sinoichnites* | [83] * | China | Lower Upper Jurassic [84] |
| *Sousaichnium* | [85] * | Brazil | Berriasian-Hauterivian [26] |
|  | [17] * | Argentina | Albian-Cenomanian? |
| *Staurichnium* | [86] * | Brazil | Berriasian-Hauterivian [26] |
|  | [35] | Brazil | Berriasian-Hauterivian [26] |
| *Taponichnus* | [86] * | Argentina | Maastrichtian |
| *Telosichnus* | [86] * | Argentina | Maastrichtian |
| *Wealdeichnites* | [83] * | Germany | Berriasian [69] |
| *Yangtzepus* | [87] * | China | Upper Lower Cretaceous [59] |

**References for Table S1**

1 Dzhalilov MR, Novikov VP (1993) Fossil dinosaur tracks in the territory of Tadzhikistan. In: Trace fossils and dynamics of extinct organism, Moscow. pp. 47-64.

2 Sternberg CM (1932) Dinosaur tracks from Peace River, British Columbia. Annual Report of the National Museum of Canada 1930: 59-85.

3 McCrea RT (2000) Vertebrate Palaeoichnology of the Lower Cretaceous (Lower Albian) Gates Formation of Alberta. M.Sc. Thesis, University of Saskatchewan, Saskatchewan , Canada. 204 p.

4 Currie PJ, Sarjeant WAS (1979) Lower cretaceous dinosaur footprints from the peace River Canyon, British Columbia, Canada. Palaeogeography, Palaeoclimatology, Palaeoecology 28: 103-115.

5 Currie PJ (1983) Hadrosaur trackways from the Lower Cretaceous of Canada. Acta Palaeontologica Polonica 28: 63-73.

6 Lucas SG, Hunt AP, Kietze KK (1989) Stratigraphy and age of Cretaceous dinosaur footprints in northeastern New Mexico and northwestern Oklahoma. In: Gillette DD, Lockley MG, editors. Dinosaur tracks and traces. Bloomington: Cambridge University Press. pp. 217-221.

7 Currie PJ (1995) Ornithopod trackways from the Lower Cretaceous of Canada. In: Sarjeant WAS, editor . Vertebrate Fossils and the Evolution of Scientific Concepts. Amsterdam: Gordon and Breach. pp. 431-443.

8 Hunt AP, Lucas SG (1998) Tetrapod ichnofaunas from the Lower Cretaceous of Northeastern New Mexico, USA. New Mexico Museum of Natural History and Science Bulletin 14: 163-168.

9 Heckert AB, Lucas SG (1998) A new dinosaur track locality from the Dakota Sandstone (Upper Cretaceous: Cenomanian) in West-Central New Mexico. New Mexico Museum of Natural History and Science Bulletin 14: 169-172.

10 Kappus EJ, Cornell WC (2003) A new Cretaceous dinosaur tracksite in Southern New Mexico. Paleontologia Electronica 6: 1-6.

11 Tanke DH (2004) Mosquitoes and mud: the 2003 Royal Tyrell Museum of Palaeontology Expedition to the Grande Prairie Region (Northwestern Alberta, Canada). Alberta Palaeontological Society Bulletin 19: 3-31.

12 Gangloff RA, May KC, Storer JE (2004) An early Late Cretaceous dinosaur tracksite in central Yukon Territory, Canada. Ichnos 11: 299-209.

13 Ishigaki S, Watabe M, Tsogtbaatar K, Saneyoshi M (2009) Dinosaur footprints from the Upper Cretaceous of Mongolia. Geological Quarterly 53: 449-460.

14 Romilio A, Salisbury SW (2011) A reassessment of large theropod dinosaur tracks from the mid-Cretaceous (late Albian–Cenomanian) Winton Formation of Lark Quarry, central-western Queensland, Australia: a case for mistaken identity. Cretaceous Research 32: 135-142.

15 Lockwood JAF, Lockley MG, Pond S (2014) A review of footprints from the Wessex Formation (Wealden Group, Lower Cretaceous) at Hanover Point, the Isle of Wight, southern England. Biological Journal of the Linnean Society Volume 113 707-720.

16 Nicosia U, Marino M, Mariotti N, Muraro C, Panigutti S, Petti FM, Sacchi E (1999) The Late Cretaceous dinosaur tracksite near Altamura (Bari, southern Italy). II. *Apulosauripus* *federicianus* new ichnogen., and new ichnosp. Geologica Romana 35: 237-247.

17 Calvo JO (1991) Huellas de dinosaurios en la Formación Rio Limay (Albiano-Cenomaniano?), Picun Leufu, Provincia de Neuquén, República Argentina. (Ornithischia-Saurischia: Sauropoda- Theropoda). Ameghiniana 28: 241-258.

18 Moratalla García JJ (1993) Restos indirectos de dinosaurios del registro español: Paleoicnología de la Cuenca de Cameros (Jurásico superior-Cretácico inferior) y Paleoología del Cretácico superior. Tesis Doctoral. PhD dissertation, Universidad Complutense de Madrid, Spain. 727 p.

19 Doublet S (2004) Contrôles tectonique et climatique de l’enregistrement stratigraphique dans un bassin continental de rift: le bassin de Cameros. PhD dissertation, Université de Bourgogne, Bourgogne, France. 512 p.

20 Melero Rubio M, Pérez-Lorente F (2011) Huellas en las obras. Reconocimiento y estudio de huellas fósiles de dinosaurio en las obras de la presa de Enciso (La Rioja. España). Zubía 29: 29-58.

21 Casamiquela RM, Fasola A (1968) Sobre pisadas de dinosaurios del Cretácico Inferior de Colchagua (Chile). Universidad de Chile, Departamento de Geología 30: 1-24.

22 Moreno K, Rubilar D (1997) Presencia de nuevas pistas de dinosaurio (Theropoda - Ornithopoda) en la Formación Baños del Flaco, Provincia de Colchagua, VI Región Chile. VIII Congreso Iberoamericano de Biodiversidad y Zoología de Vertebrados. Universidad de Concepción, 64.

23 Gabunia LK, Kurbatov V (1988) Jurassic dinosaur tracks in the south of central Asia. In: Fossils traces of vital activity and dynamics of the environment in ancient biotopes. Trans. XXX Session Union Paleontol. Soc. and VII session Ukranian paleontol. Soc. 202 p.

24 Rozhdestvensky AK (1964) New data about dinosaur localities in the territory of Kazakhstan and Middle Asia. Proceedings of the Tashkent Government University Tashkent Issue: 227-241.

25 Leonardi G (1984) Le impronte fossili dei dinosauri. In: Editrice E, editor. Sulle Orme dei deinosauri,Venezia. 161-186.

26 Novas FE (2009) The Age of Dinosaurs in South America. Bloomington: Indiana University Press, 452 p.

27 Lockley MG (1987) Dinosaur footprints from the Dakota Group of Eastern Colorado. The Mountain Geologist 24: 107-122.

28 Lockley MG (1988) Dinosaurs near Denver. In: Holden GH, editor. Field Trip guidebook, centennial meeting, Colorado School of Mines, Proffesional Contribution, 12: 288-289.

29 Currie PJ, Nadon GC, Lockley MG (1991) Dinosaur footprints with skin impressions from the Cretaceous of Alberta and Colorado. Canadian Journal of Earth Sciences 28: 102-115.

30 Matsukawa M, Futakami M, Lockley MG, Chen P, Chen J, Cao Z, Bolotsky U (1995) Dinosaur footprints from the Lower Cretaceous of eastern Manchuria, northeastern China; implications for the recognition of an ornithopod ichnofacies in East Asia. Palaios 10: 3-15

31 Lee Y-N (1997) Bird and dinosaur footprints in the Woodbine Formation (Cenomanian), Texas. Cretaceous Research 18: 849-864.

32 Schumacher BA (2003) An addition to the dinosaur freeway megatracksite, Dakota Group (Upper Cretaceous), Bent County, Colorado. Ichnos 10: 241-254.

33 Kappus EJ, Lucas SG, Hunt AP, Heckert B, Lockley MG (2003) Dinosaur footprints from the Lower Cretaceous Sarten member of the Mojado Formation at Cerro de Cristo Rey, Doña Aña County, New Mexico. Ichnos 10: 263-267.

34 Huh M, Hwang KG, Paik IS, Chung CH., Kim BS (2003) Dinosaur tracks from the Cretaceous of South Korea: Distribution, occurrences and paleobiological significance.The Island Arc 12: 132-144.

35 Leonardi G, dos Santos MCF (2004) Nes dinosaur tracksites from the Sousa Lower Cretaceous basin (Paraíba, Brasil). Studi Trent. Sci. Nat., Acta Geol. 81: 5-21.

36 Matsukawa M, Shibata K, Kukihara R, Koarai K, Lockley MG (2005) Review of Japanese Dinosaur Track Localities: Implications for Ichnotaxonomy, Paleogeography and Stratigraphic Correlation. Ichnos 12: 201-222.

37 Lockley MG, Matsukawa M, Witt D (2006). Giant theropod tracks from the Cretaceous Dakota group of Northeastern New Mexico. New Mexico Museum of Natural History and Science Bulletin 35: 83-87.

38 Lucas SG, Hunt AP (2006) Dinosaur tracks from the Upper Cretaceous Menefee Formation West Central New Mexico. New Mexico Museum of Natural History and Science Bulletin 35: 79-81.

39 Paik IS, Huh M, Park KH, Hwang KG, Kim KS, Kim HJ (2006) Yeosu dinosaur track sites of Korea: the youngest dinosaur track records in Asia. Journal of Asian Earth Sciences 28: 457-468.

40 Xing LD, Wang F, Pan S, Chen W. (2007) The discovery of dinosaur footprints from the Middle Cretaceous Jiaguan Formation of Qijiang County, Chonqing City. [en chino] Acta Geologica Sinica 81: 1591-1602.

41 Le Loeuff J, Saenyamoon T, Souillat C, Suteethorn V, Buffetaut E (2009) Mesozoic vertebrates footprints of Thailand and Laos. In: Buffetaut E, Cuny G, Le Loeuff J, Suteethorns V, editors. Late Palaeozoic and Mesozoic Ecosystems in SE Asia. London: The Geological Society 315: 245-254.

42 Kukihara R, Lockley M, Houck K (2010) Crocodile footprints from the Dakota Group (Cretaceous), John Martin Reservoir, Bent County, Colorado. New Mexico Museum of Natural History and Science Bulletin 51: 121-136.

43 Kappus EJ, Lucas SG, Langford R (2011) The Cerro de Cristo Rey Cretaceous dinosaur tracksites, Sunland Park, New Mexico, USA, and Chihuahua, Mexico. New Mexico Museum of Natural History and Science 53: 272-288.

44 Kukihara R, Lockley MG (2012) Fossil footprints from the Dakota Group (Cretaceous) John Martin Reservoir, Bent County, Colorado: new insights into the paleoecology of the dinosaur freeway. Cretaceous Research 33: 165-182.

45 Lim J-D, Lockley MG, Kong D-Y (2012) The trackway of a quadrupedal ornithopod from the Jindong Formation (Cretaceous) of Korea. Ichnos 19: 101-104.

46 Xing LD, Bell PR, Harris JD, Currie PJ (2012) An unusual, three-dimensionally preserved, large hadrosauriform pes track from "Mid"-Cretaceous Jiaguan Formation of Chongqing, China. Acta Geologica Sinica 86: 304-312.

47 Díaz-Martínez I (2013) Icnitas de dinosaurios bípedos de La Rioja (Cuenca de Cameros, Cretácico Inferior): icnotaxonomía y aplicación paleobiológica. PhD dissertation, Universidad de La Rioja, Logroño, Spain. 650 p.

48 Azuma Y, Takeyama K (1991) Dinosaur footprints from the Tetori Group, central Japan - Research of dinosaurs from the Tetori Group (4). Bulletin Fukui Prefectural Museum 4: 33-51

49 Kim HM (1986) New Early Cretaceous dinosaur tracks from Republic of Korea. In: Gillette DD, editor. First International Symposium Dinosaur Tracks Traces. Albuquerque, New Mexico. pp. 17.

50 Lockley M, Houck K, Yang S-Y, Matsukawa M, Lim S-K (2006) Dinosaur-dominated footprint assemblages from the Cretaceous Jindong Formation, Hallyo Haesang National Park area, Goseong County, South Korea: Evidence and implications. Cretaceous Research 27: 70-101.

51 Casanovas ML, Ezquerra R, Fernández A, Pérez-Lorente F, Santaté JV, Torcida F (1993) Tracks a herd of webbed Ornithopoda and other footprint found in the same site (Igea, La Rioja, Spain). Revue de Paléobiologie 7: 29-36.

52 Casanovas M, Ezquerra R, Fernández A, Pérez-Lorente F, Santafé Llopis JV, Torcida F (1995) Dos nuevos yacimientos de icnitas de dinosaurios en La Rioja y en la provincia de Soria (España). Coloquios de Paleontología 47: 9-23.

53 Pérez-Lorente F, Jiménez-Vela A (2006-07) Barranco de Valdegutiérrez: un nuevo gran yacimiento de huellas de dinosaurio en La Rioja. Zubia 18-19: 9-20.

54 Alonso RN (1980) Icnitas de dinosaurios (Ornithopoda, Hadrosauridae) en el Cretácico Superior de norte de Argentina. Acta Geologica Lilloana: 15, 55-63.

55 Jaillard E, Cappetta J, Ellenberg P, Feist M, Grambast-Fessard N, Lefranc JP, Sigé B (1993) Sedimentology, paleontology, biostratigraphy and correlation of the Late Cretaceous Vilquechico Group of Southern Peru. Cretaceous Research 14: 623-661.

56 Vialov OS (1988) On the classification of dinosaurian traces. Ezhegodnik Vsesoyuznogo Paleontologicheskogo Obshchestva 31: 322-325.

57 Lockley MG, Nadon G, Currie PJ (2003) A diverse dinosaur-bird footprint assemblages from the Lance Formation, Upper Cretaceous, Eastern Wyoming: implications for ichnotaxonomy. Ichnos 11: 229-249.

58 Suñer M, Poza B, Vila B, Santos-Cubedo A (2008) Síntesis del registro fósil de dinosaurios en el Este de la Península Ibérica. Palaeontologica Nova 8: 397-420.

59 Xing LD, Harris JD, Dong ZM, Lin YL, Wei C, Guo SB, Ji Q (2009) Ornithopod (Dinosauria: Ornithischia) tracks from the Upper Cretaceous Zhutian Formation in the Nanxiong basin, Guangdong, China and general observations of large Chinese ornithopod footprints. Geological Bulletin of China 28: 829-843.

60 Fanti F, Bell PR, Sissons RL (2013) A diverse, high-latitude ichnofauna from the Late Cretaceous Wapiti Formation, Alberta, Canada. Cretaceous Research 41: 256-269.

61 Vila B, Oms O, Fondevilla V, Gaete R, Galobart À, Riera V, Canudo JI (2013) The latest succession of dinosaur tracksites in Europe: hadrosaur ichnology, track production and palaeoenvironments. PloS One 8(9): e72579.

62 Fiorillo AR, Hasiotis ST, Kobayashi Y (2014) Herd structure in Late Cretaceous polar dinosaurs: A remarkable new dinosaur tracksite, Denali National Park, Alaska, USA. Geology 42: 719-722.

63 McCrea RT, Buckley LG, Plint AG, Currie PJ, Haggart JW, Charles W, Pemberton SG (2014) A review of vertebrate track-bearing formations from the Mesozoic and earliest Cenozoic of western Canada with a description of a new theropod ichnospecies and reassignment of an avian ichnogenus. New Mexico Museum of Natural History and Sciences Bulletin 52: 5-93.

64 Zhen S, Li J, Han Z (1996) The study of dinosaur footprints in China. Sichuan Scientific and technological Publishing House, 110 p.

65 Matsukawa M, Obata I (1994) Dinosaurs and sedimentary environments in the Japanese Cretaceous: a contribution to dinosaur facies in Asia based on molluscan palaeontology and stratigraphy. Cretaceous Research 15: 101-125.

66 Sarjeant WAS, Delair JB, Lockley MG (1998) The footprints of *Iguanodon*: a history and taxonomic study. Ichnos 6: 183-202.

67 Piñuela, L. 2000. Icnitas de Dinosaurios bípedos del Jurásico de Asturias. Morfometría, Morfología e Interpretación. M.Sc. Thesis, Universidad de Oviedo, Oviedo, Spain. 63 p.

68 Meyer CA, Thüring B (2003) The first iguanodontid dinosaur tracks from the Swiss Alps (Schrattenkalk Formation, Aptian). Ichnos 10: 221-228.

69 Diedrich C (2004) New important iguanodontid and theropod trackways of the tracksite Obernkirchen in the Berriasian of NW Germany and Megatracksite concept of Central Europe. Ichnos 11: 215-228.

70 Lockley MG, Wright JL, Thies D (2004) Some observations on the dinosaur tracks at Münchenhagen (Lower Cretaceous), Germany. Ichnos 11: 262-274.

71 Goldring R, Pollard JE, Radley JD (2005) Trace fossils and pseudossils from the Wealden strata (non-marine Lower Cretaceous) of southern England. Cretaceous Research 26: 665-685.

72 Wings O, Broschinski A Knötschke N (2005) New tridactyl dinosaur trackways from the Berriasian of Lower Saxony/Germany. 53rd symposium of Vertebrate palaeontology and comparative anatomy. The Natural History Museum, London 2005, 24.

73 Moratalla JJ, Hernán J (2008) Los S y D: dos afloramientos con icnitas de saurópodos, terópodos y ornitópodos en el Cretácico Inferior del área de Los Cayos (Cornago, La Rioja, España). Estudios geológicos 64: 161-173.

74 Pascual-Arribas C, Hernández-Medrano N, Latorre-Macarrón P, Sanz-Pérez E (2009) El icnogénero *Iguanodontipus* en el yacimiento de "Las Cuestas I" (Santa Cruz de Yanguas, Soria, España). Studia Geologica Salmanticensia 45: 105-128.

75 Diedrich C (2010) Upper Jurassic tidal flat megatracksites of Germany- coastal dinosaur migration highways between European islands, and a review of the dinosaur footprints. Palaeobiodiversity and Palaeoenvironments 91: 129-155.

76 Cobos A, Gascó F (2012) Presencia del icnogénero *Iguanodontipus* en el Cretácico Inferior de la provincia de Teruel (España). Geogaceta 52: 185-188.

77 Santos dos VF, Callapez PM, Rodrigues NPC (2013) Dinosaur footprints from the Lower Cretaceous of the Algarve Basin (Portugal): New data on the ornithopod palaeoecology and palaeobiogeography of the Iberian Peninsula. Cretaceous Research 40: 158-169.

78 Dong Z-M, Zhou Z-L, Wu S-Y (2003) Note on a hadrosaur footprint from Heilongjiang River Area of China. Vertebrata PalAsiatica 10: 324-326.

79 Reyment RAA (2004) quantitative procedure for chemostratigraphy. Stratigraphy 1: 103-104.

80 Llompart C, Casanovas ML, Santafé JV (1984) Un nuevo yacimiento de icnitas de dinosaurios en las facies garumnienses de la Conca de Tremp (Lleida, España). Acta Geológica Hispánica 19: 143-147.

81 Kim JY, Lockley MG, Kim HM, Lim J-D, Kim KS (2009) New dinosaur tracks from Korea, *Ornithopodichnus* *masanensis* ichnogen. et ichnosp. nov (Jindong Formation, Lower Cretaceous): implications for polarities in ornithopod foot morphology. Cretaceous Research 30: 1387-1397.

82 Lockley MG, Huh M, Kim KS (2012) *Ornithopodichnus* and pes-only sauropod trackways from the Hwasum tracksite, Cretaceous of Korea. Ichnos 19: 93-100.

83 Kuhn O (1958) Die fährten der vorzeitlichen Amphibien und reptilien. Verlagshaus Meisenbach. 64 p.

84 Chen P-J, Li J, Matsukawa M, Zhang H, Wang Q, Lockley MG (2006) Geological ages of dinosaur-track-bearing formations in china. Cretaceous Research 27: 22-32

85 Leonardi G (1979) Nota preliminar sobre seis pistas de dinosaurios Ornithischia da Bacia do Rio do Peixe, em Souse, Paraiba, Brasil. Anais da Academia Brasileira de Ciências 51: 501-516.

86 Alonso RN, Marquillas RA (1986) Nueva localidad con huellas de dinosaurios y primer hallazgo de huellas de aves en la Formación Yacoraite (Maastrichtiense) del Norte Argentino. Actas 4º Congreso Argentino de Paleontología y Bioestratigrafía 2: 33-41.

87 Young CC (1960) Fossil footprints in China. Vertebrata PalAsiatica 4: 53-66.
