# Supplementary material for: Ichnotaxonomic Review of Large Ornithopod Dinosaur Tracks: Temporal and Geographic Implications
Source: PLoS One. 2015 Feb 12;10(2):e0115477. doi: 10.1371/journal.pone.0115477 (PMC4326173; doi:10.1371/journal.pone.0115477)
Supplement: S1 Text — The diagnoses are in the original language. (DOCX) [file pone.0115477.s002.docx]

**Text S1. Data of large ornithopod ichnotaxa (in alphabetical order): diagnosis, holotype, type horizon and type locality.** The diagnoses are in the original language.

***Akmechetosauropus* Dzhalilov and Novikov, 1993 [1]**

Diagnosis. “Некрупные трехпалые следы (30x20 cm) с ясно видимым

отпечатком выдвинутого вперед среднего пальца (длина 5 cm, ширина в основании 12 cm). Притуплённые боковые фаланги недоразвиты и имеют длину не более 2,5 cm. Следы незначительно перекрывают линию хода. Длина шага животного - 0,6 m. Сохранность отпечатков плохая.” ([1]. p. 60).

***Akmechetosauropus makhkamovi* Dzhalilov and Novikov, 1993 [1]**

Diagnosis. As for ichnogenus.

Holotype. Fig. 4.3 (shading track).

Type horizon. Babatag-2 basin. Albian (Lowert Cretaceous).

Type locality. Luchakskaya, Tajikistan.

***Amblydactylus* Sternberg, 1932 [2]**

Diagnosis. “Very large; bípedal; tridactyle; foot very broad; toes broad and short, with proximal end enclosed in web or pad and terminating in pointed hoofs or blunt claws.” ([2]. p. 72).

Emended diagnosis after Currie and Sarjeant 1979 [3]. “Bipedal, with three functional pedal digits. The outer contours of digits II and IV diverge at low angles from the longitudinal axis of the ichnite. A distinct posterior impression is produced by a metatarsalphalangeal pad. Interdigital webs link the proximal portions of the fleshy digital pads; the digits end in blunt c1aws or pointed hooves. The ichnite is almost as wide as, or wider than, it is long.” ([3]. p. 105).

***Amblydactylus gethingi* Sternberg, 1932 [2]**

Diagnosis. As for ichnogenus.

Holotype. A paster cast, Cat- No- 8555, Geol. Surv., Canada.

Type horizon. Gething Member, Bullhead Mountain Formation. Middle Lower Cretaceous.

Type locality. Peace River Canyon, Gething, Canada.

***Amblydactylus kortmeyeri* Currie and Sarjeant, 1979 [3]**

Diagnosis. “Tridactyl pedal impressions, broader than long, with large, distinct phalangeal pads. Outline of ichnite is more strongly concave posteromedially than it is posterolaterally. The total divarication of digits II and IV is 70--80°; the anterior outline of digit IV is broad and rounded.” ([3]. p. 107).

Holotype. Provincial Museum of Alberta. Natural cast in fine-grained sandstone of a single footprint PMA P76.11.11. Paratypes BC719, BC720, PMA P77.17.6

Type horizon. Gething Formation, Bullhead Member. Lower Cretaceous.

Type locality. Peace River Canyon, Bristish Columbia, Canada.

***Apulosauripus* Nicosia, Marino, Mariotti, Muraro, Panigutti, Petti and Sacchi, 1999 [4]**

Diagnosis. “Trackway of a quadruped dinosaur, with very low pace angulation. Pedes impression slightly divergent with respect to the midline (positive rotation) while manus are forward directed and parallel to the midline. Pes and manus both (functionally) tridactyl. Manus less than half the size of the pes. The manus impressions are placed forward and medial to the pedes. No tail mark. Trackway relatively broad with a space between the interior margins of the footprints nearly 1/3 of the maximum width (wide gauge). Pace angulation very low both for pedes and manus. Stride/foot length 4:1.” ([4]. p. 238).

***Apulosauripus federicianus* Nicosia, Marino, Mariotti, Muraro, Panigutti, Petti and Sacchi, 1999 [4]**

Diagnosis. As for ichnogenus.

Holotype. Trackway ACDL 99/3. Rubber casts of three consecutive sets (ACDL 99/3-5-6-7 y ACDL 99/33-10), Museo Arqueologico di Altamura.

Type horizon. Altamura Limestone Formation. Santonian.

Type locality. De Lucia quarry, Masseria Pontrelli, Altamura (Bari), Italy.

***Babatagosauropus* Dzhalilov and Novikov 1993 [1]**

Diagnosis. “Трехпалые следы с узкой клиновидной "пяткой", короткими и разведенными в стороны боковыми фалангами (угол 50-55°). Наиболее выразительным является отпечаток среднего пальцадлина 13-15 cm, ширина близ основания - 6-8 cm. Окончания всех фа ланг закруглены, тыльная сторона следа всегда чуть длиннее наружной, чем и обусловлена его асимметрия. Общие размеры отпечатков. длина 37-43 cm, ширина 26-30 cm. Глубина следов 5-6 cm. Отпечатки касаются линии хода, но не заступают за нее.” ([1]. p. 59).

***Babatagosauropus bulini* Dzhalilov and Novikov 1993 [1]**

Diagnosis. As for ichnogenus.

Holotype. Fig. 4.4 (shading).

Type horizon. Babatag-1 Basin, Lower Cretaceous, middle-upper Albian, Luchakskaya Formation.

Type locality. Luchakskaya, Tajikistan.

***Bonaparteichnium* Calvo, 1991 [5]**

Diagnosis. “Pista de ornitópodo bípedo, tridáctilo, de tamaño grande con ángulo de paso de 170º. Posee tres dedos gruesos y cortos, de forma oval. El dedo III está más desarrollado que el II y el IV. Huellas algo asimétricas con una prolongación posterior en forma de talón que correspondería a una almohadilla plantar de tejidos conectivos y musculares.” ([5]. p. 248).

***Bonaparteichnium tali* Calvo, 1991[5]**

Diagnosis. As for ichnogenus.

Holotype. Trackway with five footprints *in situ*.

Type horizon. Coarse sandstone Candeleros Member, Río Limay Formation, Neuquén group. Albian-Cenomanian?

Type locality. Picún Leufú, Balneario Villa el Chocón, Neuquén, Argentina.

***Brachyguanodonipus* Moratalla, 1993 [6]**

Diagnosis. “Icnita de gran tamaño, entre 50 y 70 cm de longitud. Dedos anchos, robustos y muy cortos. Dedos II y IV con un desarrollo similar. Hipes simétricos. Superficie plantar muy amplia con un contorno proximal del talón muy redondeado y semicircular. No existen escotaduras talonares. Contorno general de la icnita tendente a inscribirse en un círculo. L/A entre 0,9 a 1,12. Índice BL3/z muy bajo (entre 0,35 y 0,45). Rastro bípedo, y relativamente ancho. Zancada en general corta y ocasionalmente fuerte rotación interna de las improntas.” ([6]. p. 108).

***Brachyguanodonipus prejanensis* Moratalla, 1993 [6]**

Diagnosis. As for ichnogenus.

Holotype. LMG-R1/2 tracks, concave epirrelief of La Magdalena tracksite (Préjano).

Type horizon. Enciso Group. Lower Cretaceous.

Type locality. La Magdalena tracksite, Préjano, La Rioja, Spain.

***Camptosaurichnus* Casamiquela, 1968 [7]**

Diagnosis. “Impresiones correspondientes a un dinosaurio de talla media, aparentemente en posesión de dos tipos de desplazamiento, bípedo y cuadrúpedo, y funcionalmente tridáctilo. Pasos medianos a largos y ángulo de paso de unos 160º. Impresiones de la manos muy pequeñas con relación a las del pie, ubicadas por fuera de la línea de éstas, y paralelas o anticipadas a ellas. Huellas de cola ausentes.” ([7]. p. 19).

***Camptosaurichnus fasolae* Casamiquela, 1968 [7]**

Diagnosis. As for ichnogenus.

Holotype. Rastrillada compuesta por unas seis icnitas, muy largas y angostas, en hueco, en sentido ascendente. Corresponde a la segunda del grupo de estudio según se avanza de izquierda a derecha, en sentido ascendente.

Type horizon. Basal part of Barros Formation. Berriasian? Lower Cretaceous.

Type locality. Baños de Flaco, Colchagua province, Chile.

***Camptosauropus* Gabunia and Kurbatov, 1988 [8]**

Diagnosis sensu Dzhalilov and Novikov [1]. “Трехпалые следы с широкой, массивной пяткой, имеющие близкие значения длины (28-44 cm) и ширины (26-40 cm). Отпечатки пальцев широкие, притуплённые, ширина у основания 6-12 cm; средний и правый пальцы достигают длины 18-20 cm, левый обычно равен 9-II II cm; углы между пальцами: a= 32-38°, b= 38-42°. Глубина отпечатков относительно равномерная, достигает 5-7 см; имеется неясно выраженный внешний валик.” ([1]. p. 58).

***Camptosauropus vialovii* Gabunia and Kurbatov, 1988 [8]**

Diagnosis. As for ichnogenus.

Holotype.

Type horizon. Middle-Upper Jurassic.

Type locality. Tajikistan

***Caririchnium* Leonardi, 1984 [9]**

Diagnosis. “Pista quadrupede, caratterizzata dalla notevole differenza tra le grandi orme posteriori e le piccole anteriori. Angolo del passo elevato, pista notevolmente stretta, larghezza interna con valori negativi. Orme anteriori molto piccole, di contorno ellittico con asse maggiore quasi anteroposteriore. Orme posteriori grandi, massiccie, tridattile; presenza di un cuscinetto plantare, separato dalle dita da pieghe della pelle, Dita tozze e grosse con unghie piccole e arrotondate.” ([9]. p. 177).

Emended diagnosis after Lockley, 1987 [10]*.* “Quadrupedal trackway, characterized by the remarkable difference between the large tridactyl hind footprints and the small, subeliptical hooflike fore footprints. High pace angulation, trackway quite narrow, inner width with negative value. Small eliptical foreprint with long axis directed antero-posteriorly or slightly anteromedianly to postero-laterally. Hind footprints large, tridactyl with a plantar pad separated from the toes by skin wrinkles. Toes thick and stumpy with feet showing negative inward rotation.” ([10]. p. 113).

Emended diagnosis after Lee, 1997 [11]. “Quadrupedal trackway with small elongate manus and large tridactyl plantigrade pes impressions. Bipedal trackway with large tridactyl footprints and wide trackway width. Pace angulation of quadrupedal trackway higher than bipedal. Negative rotation in both manus and pes impressions.” ([11]. p. 857).

***Caririchnium magnificum* Leonardi 1984 [9]**

Diagnosis. “Pista quadrupede di grandi dimensioni, con rilevante disparitá tra le grandi orme posteriori e le piccole anteriori. Angolo del passo devato (mano: M 172º; piede: M 145º); indice passo doppio/lunghezza dell'orma elevato (mano: M 12,1; piede: M 4.8); larghezza esterna della pista, bassa per un quadrupede (indice larghezza estena/larghezza del piede: M 1,8); larghezza interna della pista con valori negativi (indice larghezza del piede/larghezza interna: M -4,63); piede anteriore di piccole dimensioni, generalmente di forma ellittica con asse maggiore pressoché anteroposteriore, ma di forma variabile: piede posteriore grande, massiccio, tridattilo, mesassonico, con le dita costituite da una grande callositá subtriangolare nelle dita II e IV, spesso spatolate nel dilo III; con cuscinetti poco visibili, ma che indicano tre falangi nel dito II; divergenza interdigitale II-IV bassa (35º-50º); le dita presentano unghie, probabilmente più larghe che lunghe, arrotondate: cuscino plantare in forma di settore circolare di circa 100º, rotondo posteriormente; le dita sono separate dal cuscino plantare, pieghe della pelle molto visibili; presenza di alcune placche ossee rotonde. concave, nella superficie inferiore del piede; indice lunghezza piede/larghezza piede molto basso (M 1,17); asse del piede posteriore parallelo all'asse della pista o leggermente rivolto all'indietro.” ([9]. p. 177-178).

Holotype. Trackway of 25 m. Cast of the first pair manu/pes in Museu Câmara Cascudo dell'Universitá Federale di Natal.

Type horizon. Rio do Peixe Group, Antenor Navarro Formation. Basal Lower Cretaceous.

Type locality. Serrote do Pimenta village, near Sousa, Brazil.

***Caririchnium leonardii* Lockley, 1987 [10]**

Diagnosis. “As for ichnogenus except in detail of the manus and manus pace angulation. Manus shows medially directed impression of a digit which is shallower than the remainder of the forefoot impression. Manus impressions situated anteriorly and slightly lateral to the pes impression resulting in pace angulation values of about 145º which are less than those obtained for the pes.” ([10]. p. 113).

Holotype. La descripción está basada en la rastrillada A de la Figura 2 (ver Fig. 4 y 5 para detalle.

Type horizon. Dakota Group, Albian-Cenomanian.

Type locality. Alameda, Colorado, USA.

***Caririchnium protohadrosaurichnos* Lee, 1997 [11]**

Diagnosis. “In quadrupedal tracks, small elongate manus impression located anteriorly between digits III and IV of the pes impression. Long axis of manus print directed slightly anteromedially. Outer margins of digit II and IV impressions within an angle of approximately 10º. Foot length greater than width. Divarication of digits II and IV is 56º. In bipedal tracks, digits II and IV claw impressions pointed, unlike blunt digit III. Foot width is greater than length. Divarication of digits II and IV is 64º. Negative rotation in bipedal trackway stronger than in quadrupedal.” ([11]. p. 857).

Holotype. Artificial casts of left footprint, number 4 in trackway 3 (SMU 74652),

and right manus and pes prints, number 4 of trackway 4 (SMU 74653).

Type horizon. Woodbine Formation, Cenomanian.

Type locality. On the north shore of Grapevine Lake, Denton County, Texas, USA.

***Caririchnium lotus* Xing, Wang, Pan and Chen, 2007 [12]**

Diagnosis. **“**大型鸟脚类恐龙足迹, 后足迹的宽度与长度比率为 0,75; 第Ⅱ和第Ⅲ趾之间的夹角约为 25º; 第Ⅲ和第Ⅳ 趾之间的夹角约为 25º; 足迹后部有丘 状的跖骨印痕; 复步角为 161º; 足长与复步的比为 1: 5,65; 前足迹位于后足迹第Ⅲ-Ⅳ趾间之前；宽度 与长度比率为 0,53; 第Ⅱ和第Ⅲ指之间的夹角约为 13º; 第Ⅲ和第Ⅳ指之间的夹角约为 9º; 足迹后部有 椭圆状的掌骨印痕; 复步角为 141º; 足长与复步的 比为 1:12; 与相邻的后足迹面积比约为 1:7,3; 亚 成年个体的后足迹除第Ⅲ趾略长于第Ⅱ-Ⅳ趾外, 该 后足迹与成年个体后足迹完全一致.” ([12]. p. 1596).

Holotype. QJGM-T37-3

Type horizon. Jiaguan Formation, “mid” Cretaceous.

Type locality. Sanjiao, Condado de Qijiang, China.

***Caririchnium kyoungsookimi* Lim, Lockley and Kong, 2012 [13]**

Diagnosis. “Trackway of a typical large ornithopod with strong heteropody; pes large, tridactyl but manus small, crescentic in outline, enclosing three equidimensional, circular digit traces.” ([13]. p. 102).

Holotype. NHCG 10194, partial trackway with two successive manus-pes sets.

Type horizon. Jindong Formation. Upper Aptian.

Type locality. Duhori area, Goseong County, Korea.

***Gigantoshiraminesauropus* Azuma and Takeyama, 1991 [14]**

Diagnosis. “A new *Gigantoshiraminesauropus* is monotypic at present, with tridactyl imprint, lacking digits I and V, mesaxonic foot structure. Bipedal. Tridactyl big pes with digits II-IV impressed. The imprint of digit III is longest. The imprint of digit II is longer than digit IV. The angle between digits IV and II is larger than the one between digits III and II. Phalangeal pads of digits II, III and IV are big and high, but posterior parts of pads are not defined and rise of pads continue to sole-callus.” ([14]. p. 40).

***Gigantoshiraminesauropus matsuoi* Azuma and Takeyama, 1991 [14]**

Diagnosis. As for ichnogenus.

Holotype. ISBV002. A single natural cast, collected by Ichio Yamaguchi in 1985.

Type horizon. Kuwajima Member, upper part of the Itoshiro subgroup. Berriasian-Barremian (Lower Cretaceous).

Type locality. Shiramine Village, Ishikawa Prefecture, Japan.

***Goseongosauripus*  Kim, 1986 [15]**

Diagnosis. Not given.

***Goseongosauripus kimi* Kim, 1986 [15]**

Diagnosis. Not given.

Holotype.

Type horizon. Jindong Formation. Upper Aptian [13].

Type locality. Goseong-gun, Korea [16].

***Gypsichnites* Sternberg, 1932 [2]**

Diagnosis. “Bipedal; semidigitigrade; tridactyle; heel broadly rounded and complete; foot short and broad; toes broad, partly enclosed in pad or web and terminating in bluntly pointed hoofs.” ([2]. p. 68).

Emended diagnosis after McCrea, 2000 [17]. The footprints of a tridactyl, semi-digitigrade biped. The footprint is longer than wide. Some observed specimens display lateral digits that are detached from the rest of the footprint. Digits are broad with distal portions often displaying distinct swelling, especially digit III. Distinct terminal claws are evident on some specimens of this ichnogenus which cannot be classified as "bluntly pointed hoofs".

***Gypsichnites pacensis* Sternberg, 1932 [2]**

Diagnosis. As for ichnogenus.

Holotype. Cat. No. 8553. Geol. Surv., Canada.

Type horizon. Gething Member, Bullhead Mountain Formation. Middle Lower Cretaceous.

Type locality. Peace River Canyon, Gething. Canada.

***Hadrosaurichnoides* Casanovas, Ezquerra, Fernández, Pérez-Lorente, Santafé and Torcida, 1993 [18]**

Diagnosis. “Trackway of bipedal animal, tridactyl. Toes with just one diamond shaped pad. The heel is formed by another wide and oval pad, whose large axis is perpendicular to the third toe axis. This pad is separated from them by a little elevation in the bottom of the mark. Between toes, there is a interdigital web, showing wrinkle marks, like radius from the hipex to the outer part, extending between the adjacent toes. The foot is a bit longer than wide: l= 38,5 cm, a= 35 cm. Relation (l-a)/a= 0,1. The trackway is narrow. The interdigital angle, measured with the toe axis, is low. There are no very sharply defined hooves. The II-III-IV toes length is 12-19-12 cm.” ([18]. p. 33).

***Hadrosaurichnoides igeensis* Casanovas, Ezquerra, Fernández, Pérez-Lorente, Santafé and Torcida, 1993 [18]**

Diagnosis. As for ichnogenus.

Holotype. Footprint number 3 in number 11 trackway. Material in loco.

Type horizon. Footprints are exposed in a limestone bed, black in a fresh cutting, with a N145E direction and a 15NE dip. The bed is in "Alternancias samitico-peliticas con calizas y margas intercaladas" bedding at the top of Enciso Group. Lower Cretaceous, probably Aptian.

Type locality. Igea, La Rioja, España.

***Hadrosaurichnus* Alonso, 1980 [19]**

Diagnosis. “Icnitas en hueco pertenecientes a un animal bípedo, tridáctilo, de talla mediana a grande, correspondiente a un andar caminando y ágil. Valor de paso grande lo que evita que las pisadas se sobrepongan. Luz de rastrillada pequeña a negativa. Presencia de pequeñas pezuñas y aparente membrana interdigital cubriendo los dedos. Sin arrastre de cola.” ([19]. p. 56).

***Hadrosaurichnus australis* Alonso, 1980 [19]**

Diagnosis. As for ichnogenus.

Holotype. Rastro de tres icnitas en hueco. Conservación buena y accesible a medición directa. Moldes en yeso de dos impresiones correspondientes al holotipo (CNS:10020) en la Cátedra de Paleontología, Departamento de Ciencias Naturales, Universidad Nacional de Salta, Argentina.

Type horizon. Upper part of Yacoraite Formation, Balbuena Subgroup, Salta Group. Maastrichtian (Upper Cretaceous).

Type locality. Quebrada de la Escalada, San Carlos, Salta, Argentina.

***Hadrosaurichnus titicaensis* Ellenberger, 1993 [20]**

Diagnosis. “For generic diagnosis see [19]; specific diagnosis: smaller than *H*. *australis*, with median digit III comparatively shorter, lateral digits II and IV even more divergent, gate a bit shorter, width of track a bit less.” ([20]. p. 638).

Holotype. Material in situ, Vilquechico, southern Peru (basal level of UVF) with three successive steps in hyporelief.

Type horizon. Upper part of Vilquechico Formation. Campanian- Maastrichtian. Upper Cretaceous

Type locality. Near Tititaca lake and Vilquechico, Peru.

***Hadrosauripeda* Vialov, 1988 [21]**

Diagnosis: Not given.

***Hadrosauripeda hauboldi* Vialov, 1988 [21]**

Diagnosis: Not given.

Holotype: Vialov [21] proposed the fig. 54.9 p. 87 of Haubold [22] as holotype, that correspond with the cast N.M.C. No. 9487 deposited National Museum of Canada of Langston [23] fig.1-2.

Type horizont: St. Mary River Formation [23].

Type locality: Alberta ,Canada [23].

***Hadrosauropodus* Lockley, Nadon and Currie, 2003 [24]**

Diagnosis original. “Trackway of a large three-toed biped. Tracks as wide or wider than long. Each toe impression consists of an oval pad with long axis parallel to track axis. Track axis rotated inward relative to trackway mid line. Step short, about 2 × foot length. Heel rounded transverse or posteriorly concave with bilobed posterior margin. May be associated with small manus tracks.” ([24]. p. 240).

***Hadrosauropodus langstoni* Lockley, Nadon and Currie, 2003 [24]**

Diagnosis. As for ichnogenus.

Holotype TMP 87.76.7, a pes track found with associated manus and topotype/paratype trackway segments on the same surface.

Type horizon. St. Mary River Formation. Late Cretaceous (Maastrichtian).

Type locality. St.Mary River valley, about 20 km SSW of Lethbridge, Alberta Canada.

***Hadrosauropodus nanxiongensis* Xing, Harris, Dong, Lin, Chen, Gou and Ji, 2009 [25]**

Diagnosis. “Mid-sized *Hadrosauropodus*. Trydactyl tracks that are longer than wide and that lack manus and tail traces; angles between digits II and IV range from 51º to 95º; round convexity on the caudolateral margin of digit II; maximum width of digit IV is roughly twice that of digit II.” ([25]. p. 837).

Holotype. All specimens occur in three trackways (YMK.A-YMK.C) of natural pes print casts; YMK.A consists of three tracks, YMK.B of five tracks, and YNK.C of four tracks. Of these, the best preserved (YMK.A2, of which NDM.F1 is a cast) is designated as the holotype.

Type horizon. Zhutian Formation. Maastritchian, Upper Cretaceous.

Type locality. Yangmeikeng tracksite, Shaoguan, Guangdong, China.

***Iguanodonichnus* Casamiquela, 1968 [7]**

Diagnosis. “Impresiones correspondientes a un dinosaurio de gran talla, bípedo y con tres dedos funcionales por lo menos. Pasos variablemente espaciados pero por lo general tan cortos como para que las impresiones no alcancen a sobrepasarse netamente. Luz de rastrillada pequeña en relación con el gran tamaño de las pisadas. Huellas de cola ausentes.” ([7]. p. 14).

***Iguanodonichnus frenkii* Casamiquela, 1968 [7]**

Diagnosis. As for ichnogenus.

Holotype. Nº 3 of a trackway of 12 elongate tracks.

Type horizon. Basal part of Barros Formation. Berriasiense? Cretácico Inferior.

Type locality. Baños de Flaco, Colchagua Province, Chile.

***Iguanodonipus* Moratalla, 1993 [6]**

Diagnosis original. “Icnita de media y gran talla. Talla más normal entre 40 y 60 cm de longitud, aunque puede ser mayor. Dígitos anchos, cortos y robustos con la terminación distal redondeada. Desarrollo similar de los dígitos II y IV. Hipes simétricos. Superficie plantar amplia con el contorno proximal del talón redondeado. Dos escotaduras medial y lateral, ocasionalmente muy marcadas, en talón. Índice BL3/z superior a *Brachyguanodonipus*, entre 0.6 y 1.3. Rastro bípedo y relativamente ancho; icnitas con rotación interna. Ocasionalmente rastros cuadrúpedos, con las improntas delanteras transversalmente alargadas y bilobuladas, con orientación externa. Disposición de las manos delante de las huellas traseras, algo lateralizadas, frente al hipes lateral o incluso más externamente. Alto índice de heteropodia.” ([6]. p. 109).

***Iguanodonipus cuadrupedae* Moratalla, 1993 [6]**

Diagnosis. As for ichnogenus.

Holotype. El Frontal tracksite. RGML. R1/3.

Type horizon. Urbión Group. Lower Cretaceous.

Type locality. Regumiel de la Sierra, Burgos, Spain.

***Iguanodonopus* Zhen, Li and Hang, 1996 [26]**

Diagnosis. “特征两足行走，三趾型，半趾行式，趾末端钝, 跟部连接皿、IV趾基部，II趾短 各 趾较宽, 足迹一般较大(长在 30-70cm 左右)，足长与复步之比为 1:3, III趾稍向内偏转, 无前足及尾部印迹。.” ([26]. p. 73).

***Iguanodonopus xingfuensis* Zhen, Li and Hang, 1996 [26]**

Diagnosis. As for ichnogenus.

Holotype. CFEC-E-1 a y b, footprint and natural cast.

Type horizon. Lower Cretaceous?

Type locality. Mindanao River County, Sichuan Province, China.

***Iguanodontipus* Sarjeant, Delair and Lockley, 1998 [27]**

Diagnosis. “Tridactyl pedal imprints of a dinosaur, semidigitigrade, all three digits being of similar length. Central digit (III) directed forward and approximating to an equilateral triangle in shape. Digits II and IV directed almost laterally; they are somewhat less broad and have the form of isosceles triangles with rounded to sub-acute distal ends. Posterior of sole smoothly curved or very slightly flattened. Claws not defined. Trackway narrow: stride long.” ([27]. p. 195).

***Iguanodontipus burreyi* Sarjeant, Delair and Lockley, 1998 [27]**

Diagnosis. As for ichnogenus.

Holotype. Type series: seven natural casts preserved on three slabs, the holotype being specimen no. BNSS 33793b, the slab bearing casts C and D, a right and a left pes (Fig. 13, lower, and Fig. 14, lower); the paratypes being specimen nos. BNSS 33793a and c, the slabs bearing casts A and B and E to G (Fig. 13, upper and Fig. 14, upper). Lodged in the Geological Museum of Bournemouth Natural Science Society (BNSS), 39 Christchurch Road, Boscombe, Bournemouth, Hampshire, England.

Type horizon. Lower Durlston Beds (Middle Purbeck Beds), Early Cretaceous (Berriasian).

Type locality. Norman's Quarry, Queensground, Langton Matravers, Dorset, England.

***Iguanodontipus billsarjeanti* Meyer and Thüring, 2003 [28]**

Diagnosis. “Tridactyl pes wider than long, resulting in a footprint ratio between 0.92 and 1.06. Individual digits elongate, slightly rounded with interdigital angles that range between 30° and 35°. Digit III directed forward ending in a slightly rounded equilateral triangle, but slightly rounded. Interdigital angles in all trackways consistently between 30° and 35°. Pace angulation between 150° and 175°. In trackway I (RP 2; Fig. 6), pad impressions can be observed. Pes length varies from 28 to 35 cm. The heel is triangular to smoothly rounded, claws are not defined. The manus is oval to almost round and lies close to the anterior of the apices of digits III and IV. All the trackways show slight inward rotation of the axes of pes of the third pedal digits. Pace measures between 62.4 and 71.7 cm.” ([28]. p. 224).

Holotype. A cast of trackway segment T1 (NMB K.S. 374) is kept in the Natural History Museum Basel (Figs. 8, 9).

Type horizon. Upper Schrattenkalk Formation, age. Middle Lower Aptian to Middle Upper Aptian.

Type locality. Risleten Quarry, Swiss Coord. 682 649/ 202 413, 428 m.a.sl. (quarry base).

***Jianyinosauropus* Dong, Zhou and Wu, 2003 [29]**

Diagnosis. “**记述和讨论**　正型标本是一左脚印迹,保存在一 厚层钙质胶结的粗砂岩板上,岩板长128cm, 宽50cm, 厚约7cm. 岩板脱层从悬崖滚落在江边。保存下来的足印呈凸形,应是上模。足印呈三趾 型,宽大于长(足迹保存长 40cm, 宽 45cm). 三个趾都 比较粗壮,趾尖均具扁爪(蹄状) 印痕,中趾( III) 呈U 型,长 18cm. 趾II 较短,趾长 11cm ,有趾垫,趾IV 较趾 II 长,其长为 15cm. 趾II 与趾III 的夹角 (interdigital angle) 约42°, III、IV 趾之夹角 37°. 趾间有蹼的印迹。足印的后缘圆滑.” ([29]. p. 325).

***Jianyinosauropus johnsoni* Dong, Zhou and Wu, 2003 [29]**

Diagnosis. As for ichnogenus.

Holotype. J F1(=JDGP V.o1), National Geologic Park of Dinosaurs Jiayin). A cast in a slab.

Type horizon. Yongancun Formation, Upper Cretaceous.

Type locality. Jiayin County, Heilongjiang, China.

***Kharkushosauropus* Dzhalilov and Novikov, 1993 [1]**

Diagnosis. “Гигантские трехпалые следы с относительно узкой пяткой и широко поставленными массивными фалангами. Расстояние между боковыми фалангами (максимальная ширина следа) 55-60 cm, угол между ними - 50°. Ширина у пяточной части около 20 cm, полная длина следа до 72 cm. Длина пальцев составляет 27-35 cm, ширина от 10 до 14 cm. Судя по форме и рельефу отдельных отпечатков, можно предполагать, что фаланги животного были снабжены острыми когтями и обладали подвижными суставами. Характерна значительная глубина отпечатков в передней части стопы (до 6-7 cm), наличие здесь ярко выраженных валиков отжатого грунта и одновременно - слабая "прорисовка" зафалан- гового отдела.” ([1]. p. 59).

***Kharkushosauropus kharkushensis* Dzhalilov and Novikov, 1993 [1]**

Diagnosis. As for ichnogenus.

Holotype.

Type horizon. Karabilskaya Formation, Titonian, Upper Jurassic.

Type locality. Hissar, Harkush, Tajikistan.

***Limayichnus* Calvo, 1991 [5]**

Diagnosis. “Rastro de un dinosaurio bípedo con icnitas tridáctilas grandes. El ángulo de paso es mayor de 150º y menor de 170º. Las huellas son simétricas, sin impresión de talón y uñas. Los dedos son cortos y tienen terminación distal redondeada. El contorno posterior es redondeado. Los dedos II y IV divergen ligeramente del dedo III, con un ángulo de 25º o menor. El dedo III tiene una almohadilla ovalada, que está separada de la almohadilla plantar. Los dedos II y IV tienen una única almohadilla con forma de U. Los rastros preservados muestran cambios de dirección cada tres huellas sucesivas.” ([5]. p. 246).

***Limayichnus major* Calvo, 1991 [5]**

Diagnosis. As for ichnogenus.

Holotype. MUCPv-65 cast. Trackways MUCPv-66, -70 y –73.

Type horizon. Candeleros Formation, Neuquen Group. Albian-Cenomanian?

Type locality. Nueva peninsule and Cerrito del Bosque Island, Picún Leufú, Balneario Villa el Chocón, Neuquén, Argentina.

***Orcauichnites* Llompart, Casanovas and Santafé, 1984 [30]**

Diagnosis. “Huella perteneciente probablemente a un animal bípedo, de talla pequeña. Pie tridáctilo, con la anchura máxima casi igual a la longitud total. Dedos ligeramente estilizados, con el central algo más largo que los laterales; sin terminación en garras. Longitud total del pie entre 23 y 25 cm. Talón redondeado, y relativamente grande y ancho. La longitud de la zancada y el ángulo de paso se desconocen.” ([30]. p. 146).

***Orcauichnites garumniensis* Llompart, Casanovas and Santafé, 1984 [30]**

Diagnosis. As for ichnogenus.

Holotype. A track belonging to level III of the calcareous limestone level with bioturbations Maastrichtian in age, located on the east of the town of Orcau (Tremp Basin) on the left side of the road to Bastús Orcau.

Type horizon. Grey micritic limestones. Maastrichtian.

Type locality. Orcau, Tremp, Lleida, Spain.

***Ornithopodichnites* Llompart, Casanovas and Santafé, 1984 [30]**

Diagnosis. “Huellas pertenecientes probablemente a un animal bípedo de talla grande. Pie tridáctilo, más largo que ancho; dígitos cortos y anchos con el central bastante más largo que los laterales; sin terminación en garras. Longitud total de la Icnita entre 43 y 67 cm. Talón relativamente grande y ancho. La longitud de la zancada y el ángulo de paso se desconocen.” ([30]. p. 147).

***Ornithopodichnites magna* Llompart, Casanovas and Santafé, 1984 [30]**

Diagnosis. As for ichnogenus.

Holotype. A track belonging to level III of the calcareous limestone level with bioturbations Maastrichtian in age, located on the east of the town of Orcau (Tremp Basin) on the left side of the road to Bastús Orcau.

Type horizon. Grey micritic limestones. Maastrichtian.

Type locality. Orcau, Tremp, Lleida, Spain.

***Ornithopodichnus* Kim, Lockley, Kim, Lim and Kim, 2009 [31]**

Diagnosis. “Narrow trackway of a large facultative biped. Weakly mesaxonic tridactyl pes tracks with very thick, broad U-shaped digit impressions shallowly separated only in the distal part and with smoothly rounded hind margin. Total divarication of digits II-IV about 65–70", interdigital angle of digits II-III smaller than those digits III-IV. Pes pace angulation about 170", tracks rotated positively (inwards). Ratios of pes stride to track length about 4.1–4.6. External trackway width narrow up to 69 cm, about 1.6 times track width. Manus traces may be present in some trackways. .” ([31]. p. 1390).

***Ornithopodichnus masanensis* Kim, Lockley, Kim, Lim and Kim, 2009 [31]**

Diagnosis. As for ichnogenus.

Holotype. NHC1001 (Trackway 5, represented by A in figs. 4–6): paratypes-NHC 1002w1005 (trackways 1–4, see figs. 4–6) displayed in a covered exhibit at the Natural Heritage Center (NHC) of the National Research Institute of Cultural Heritage,

Daejeon, Korea.

Type horizon. Fine-grained sandstone bed, lower part of the Jindong Formation (Lower Cretaceous, Hayang Group).

Type locality. Road cut between Daepyeongri and Jisanri villages, Jinbuk-myeon, Masan City, 128º40´E, 35º10´N. About 40 km west from Busan City and about 40 km northeast of the Goseong dinosaur tracksite.

***Shiraminesauropus* Azuma and Takeyama, 1991 [14]**

Diagnosis. “A new ichnogenus *Shiraminesauropus* is characterized by tridactyl imprint lacking digits I and V, mesaxonic foot structure. Bipedal. Tridactyl small pes with digits II-IV impressed. The imprint of digit III is longest and widest. The imprint of digit II is shorter than digit IV. The angle between digits IV and III is larger than the one between digits III and II. Phalangeal pads of digits II, III and IV made an oblong impression each. The sole-callus is shown clearly.” ([14]. p. 37).

***Shiraminesauropus reini* Azuma and Takeyama, 1991 [14]**

Diagnosis. “Type specimen of *Shiraminesauropus* *reini* is a single natural cast. The specimen is a left pes imprint; small in size with distinct digital pads and sole-callus; tridactyl in shape; lacks digits I and V; mesaxonic foot structure. The sole length is short, less than half of FL. Footprint length is 16 cm, length of digit II about 6,0 cm free length of digit III about 9,5 cm and length of digit IV about 7,9 cm with U-shaped outline. Footprint width is 12 cm. The anterior part of digit IV is curved to be parallel to the longitudinal axis. Interdigital angles II-II and III-IV are about 25º and approximately 29º, respectively. The total divarication of digits II-IV is some 54º. Posterior half of the footprint is U-shaped in outline.” ([14]. p. 38).

Holotype. FPMN 850321, a single natural cast, collected by Ken-ichi Takeyama, Yoichi Azuma and Tetsuji Araki in 1985.

Type horizon. From the Kuwajima member, the upper part of the Itoshiro sungroup; Early Cretaceous (Berriasian-Barremian).

Type locality. Shiramine village, Ishikawa prefecture, Japan.

***Shiraminesauropus hayashidaniensis* Azuma and Takeyama, 1991 [14]**

Diagnosis. “The type specimen of *Shiraminesauropus hayashidaniensis* is a single natural cast. The specimen is left imprint; small in size; a foot with the distinct clear digital pads and sole-callus; tridactyl in shape lacking digits I and V; mesaxonic foot structure. The sole length is long, more than half of FL. Footprint length is 21 cm, length of digit II about 6,9 cm free length of digit III about 9,0 cm and length of digit IV about 7,6 cm with U-shaped outline. Footprint width is 12,5 cm. The anterior part of digit IV is curved to be parallel to the longitudinal axis. Interdigital angles II-III and III-IV are about 14º and about 29º, respectively. The total divarication of digits II-IV is approximately 43º.” ([14]. p. 39).

Holotype. FPMN 900881, a single natural cast, collected by Kotohiro Jochi in 1989.

Type horizon. From the Itsuki shale member, the upper part of the Itoshiro subgroup; Early Cretaceous (Berriasian-Barremian).

Type locality. Izumi village, Fukui prefecture, Japan.

***Sinoichnites* Kuhn, 1958 [31]**

Diagnosis. “Tridactyl, biped, Zehen groß, breit, vorne gerundet und ohne Krallen. Whol herbivore. Ornithischier, ähnlidh Jeholosauripus, aber viel plumper. Länge 30 cm, Breite 33 cm.” ([31]. p. 24).

***Sinoichnites youngi* Kuhn, 1958 [31]**

Diagnosis. As for ichnogenus.

Holotype.

Type horizon. Cretaceous?

Type locality. China.

***Sousaichnium* Leonardi, 1979 [32]**

Diagnosis original. “Pista de dinossauro bípede de grande porte, com àngulo do passo de valor alto a muito alto; pegadas bem distanciudas, nào se alcaçando uma com a outra, e portando alevado valor da razào passo duplo; comprimento pè luz la pista com valor negativo; linha mediana da pista característicamente serpentina, com o resultado que as pegadas se mudam em grupos de très da direita para a esquerda e vice-versa. Eixo das pegadas paralelo da pista ou pouco inclinado para dentro ou para fora. Pegadas tridáctilas, quase smétricas, digitigradas ou mais provavalmente semidigitigradas, com prevalencia funcional na almofada, enquanto entre os dedos prevalece, funcionalmente o III ou o IV. Dedos cortos e grossos, de forma oval, sem marcas distintas de garras ou una bem como de almofadas, divergencias entre od dedos, total e parciais, baixas o comprimento os dedos aumenta do II ao IV, ficando porém o III em posiçáo mais avançada que os demaís: impressóes de dobras da pele bem evidentes entre os dedos e entre estes e a almofada, esta redonda e bem ampla com relaçáo a superficie ocupada pelos dedos margem trazeira em forma de “talon”.” ([32]. p. 505).

***Sousaichnium pricei* Leonardi, 1979 [32]**

Diagnosis. As for ichnogenus.

Holotype. Trackway of 32 footprints (SOPPa).

Type horizon. Rio do Peixe Group, Sousa Formation. Lower Cretaceous or Upper Jurassic.

Type locality. Pasagem das Pedras, near Sousa, Paraiba, Brazil.

***Sousaichnium monettae* Calvo, 1991 [5]**

Diagnosis. “Icnita muy grande, tridáctila, con el dedo central un poco más largo que en *Sousaichnium pricei*. El dedo IV un poco más grande que el II y este último con una mayor divergencia con respecto al dedo III. Dedo III y talón algo desplazados internamente, a diferencia de *S. pricei*, que está en posición central. Dedo III de bordes paralelos y no oblicuos como en la forma brasileña. Hipex más agudo que en *S. pricei*.” ([5]. p. 245).

Holotype. A left track *in situ*.

Type horizon. Fine sandstone of Candeleros Member, Rio Limay Formation, Neuquén Group. Albiense-Cenomaniense?

Type locality. Picún Leufú, Balneario Villa el Chocón, Neuquén, Argentina.

***Staurichnium* Leonardi, 1979 [32]**

Diagnosis. “Pista de dinosauro bipede de dimensòes médias, àngulo do passo elevado, com valores ao redor de 160º na marcha normal; eixo do pé ligeiramente e variadamente inclinado com relaçào ao eixo da pista. Pegada tridáctila, simétrica, formada pelas impressoes redondas ou ovais de très dedos em forma de cascos, separadas por cristas que representam a impressào de dobras da pele; e pelo vestigio de uma almofada de forma variavel. O casco central (dedo III) é maior dos outros; o lateral ligeiramente maior do que o medial. A impressào da almofada é por vezes redonda, por vezes oval, com eixo maior no sentido quer ànteroposterior, quer transversal. No conjunto a pegada apresenta aspecto mui característico de trevo ou de cruz de braços arredondados.” ([32]. p. 512).

***Staurichnium diogenis* Leonardi, 1979 [32]**

Diagnosis. As for ichnogenus.

Holotype. Trackway SOPPf.

Type horizon. Rio do Peixe Group, Sousa Formation. Lower Cretaceous or Upper Jurassic.

Type locality. Pasagem das Pedras, near Sousa, Paraiba, Brazil.

***Taponichnus* Alonso and Marquillas, 1986 [33]**

Diagnosis original. “Icnita excesivamente más larga que ancha, con impresión poco marcada de los dígitos, tridáctila, ausencia de uñas, con forma de escudo o pata de pato.” ([33]. p. 38).

***Taponichnus donottoi* Alonso and Marquillas, 1986 [33]**

Diagnosis. As for ichnogenus.

Holotype. Material “in loco”. An isolated track, well preserved, oriented W-E.

Type horizon. Yacoraite Formation, Maastrichtian (Upper Cretaceous).

Type locality. Valle del Tonco, Salta, Argentina.

***Telosichnus* Alonso and Marquillas, 1986 [33]**

Diagnosis. “Icnita grande redondeada, con dígitos III y IV romos y con marcados surcos y rebabas en el sedimento. Ausencia de uñas.” ([33]. p. 38).

***Telosichnus saltensis* Alonso and Marquillas, 1986 [33]**

Diagnosis. As for ichnogenus.

Holotype. Material “in loco”. An isolated track, well preserved but incomplete, oriented SE-NW.

Type horizon. Yacoraite Formation, Maastrichtian (Upper Cretaceous).

Type locality. Valle del Tonco, Salta, Argentina.

***Wealdenichnites* Kuhn, 1958 [31]**

Diagnosis. “Hinterfubabdruck erinnert stark an *Iguanodon* and *Gypsichnites* aus der UnterKreide. Aber der Abdruck ist hinten länger und im ganzen schlanker, dazu kommt ein kleiner Hallux. Vermutlich ein primitive Iguanodontide.” ([31]. p. 27).

***Wealdenichnites iguanodontoides* Kuhn, 1958 [31]**

Diagnosis. As for ichnogenus.

Holotype.

Type horizon. Od the Obernkirchen sandstone.

Type locality. Obernkirchen, Alemania.

***Yangtzepus* Young, 1960 [34]**

Diagnosis. “Tridactyle footprints with the three digits closely connected. The lateral digits of the hand divergent distinctly. Lateral digits of the foot rather long and sub-equal in length, III well separated from the heel. The pad number of the foot clearly shown, 2 in II ; 3 in both III and IV. Certainly plantigrade. Skin impressions coarsely granulated.” ([34]. p. 62).

Emended diagnosis after Xing et al. 2009 [25]. “Medium-sized, tridactyl theropod (originally attibuted to an ornithopod) tracks that lack manus and tail traces. The divarication angle between digits II and III is wider than that between digits III and IV. Digit III is directed forward, and digit IV is similar to digit II in length. The long axis os each digit is parallel to the track axis. A metatarsophalangeal pad is oval in shape.” ([25]. p. 832).

***Yangtzepus yipingensis* Young, 1960 [34]**

Diagnosis. As for ichnogenus.

Holotype. Three footprints one of manus and two of pes. The best preserved and

large one is selected as the type and the other two as co-types, field number Ao88, No.

42. Cat. number of the Institute, V.2473.

Type horizon. Upper Jurassic of the lower part of the Chiating Series from

Jiading Grup, Chiating Serie.

Type locality. Kuanyinchon, Kaichinhsiang, Kuanyin district, Yiping, de Sichuan, China.

**References for Text S1**

1 Dzhalilov MR, Novikov VP (1993) Fossil dinosaur tracks in the territory of Tadzhikistan. In: Trace fossils and dynamics of extinct organism, Moscow. pp. 47-64.

2 Sternberg CM (1932) Dinosaur tracks from Peace River, British Columbia. Annual Report of the National Museum of Canada 1930: 59-85.

3 Currie PJ, Sarjeant WAS (1979) Lower cretaceous dinosaur footprints from the peace River Canyon, British Columbia, Canada. Palaeogeography, Palaeoclimatology, Palaeoecology 28: 103-115.

4 Nicosia U, Marino M, Mariotti N, Muraro C, Panigutti S, Petti FM, Sacchi E (1999) The Late Cretaceous dinosaur tracksite near Altamura (Bari, southern Italy). II. *Apulosauripus* *federicianus* new ichnogen., and new ichnosp. Geologica Romana 35: 237-247.

5 Calvo JO (1991) Huellas de dinosaurios en la Formación Rio Limay (Albiano-Cenomaniano?), Picun Leufu, Provincia de Neuquén, República Argentina. (Ornithischia-Saurischia: Sauropoda- Theropoda). Ameghiniana 28: 241-258.

6 Moratalla García JJ (1993) Restos indirectos de dinosaurios del registro español: Paleoicnología de la Cuenca de Cameros (Jurásico superior-Cretácico inferior) y Paleoología del Cretácico superior. Tesis Doctoral. PhD dissertation, Universidad Complutense de Madrid, Spain. 727 p.

7 Casamiquela RM, Fasola A (1968) Sobre pisadas de dinosaurios del Cretácico Inferior de Colchagua (Chile). Universidad de Chile, Departamento de Geología 30: 1-24.

8 Gabunia LK, Kurbatov V (1988) Jurassic dinosaur tracks in the south of central Asia. In: Fossils traces of vital activity and dynamics of the environment in ancient biotopes. Trans. XXX Session Union Paleontol. Soc. and VII session Ukranian paleontol. Soc. 202 p.

9 Leonardi G (1984) Le impronte fossili dei dinosauri. In: Editrice E, editor. Sulle Orme dei deinosauri,Venezia. 161-186.

10 Lockley MG (1987) Dinosaur footprints from the Dakota Group of Eastern Colorado. The Mountain Geologist 24: 107-122.

11 Lee Y-N (1997) Bird and dinosaur footprints in the Woodbine Formation (Cenomanian), Texas. Cretaceous Research 18: 849-864.

12 Xing LD, Wang F, Pan S, Chen W. (2007) The discovery of dinosaur footprints from the Middle Cretaceous Jiaguan Formation of Qijiang County, Chonqing City. [en chino] Acta Geologica Sinica 81: 1591-1602.

13 Lim J-D, Lockley MG, Kong D-Y (2012) The trackway of a quadrupedal ornithopod from the Jindong Formation (Cretaceous) of Korea. Ichnos 19: 101-104.

14 Azuma Y, Takeyama K (1991) Dinosaur footprints from the Tetori Group, central Japan - Research of dinosaurs from the Tetori Group (4). Bulletin Fukui Prefectural Museum 4: 33-51

15 Kim HM (1986) New Early Cretaceous dinosaur tracks from Republic of Korea. In: Gillette DD, editor. First International Symposium Dinosaur Tracks Traces. Albuquerque, New Mexico. pp. 17.

16 Lockley M, Houck K, Yang S-Y, Matsukawa M, Lim S-K (2006) Dinosaur-dominated footprint assemblages from the Cretaceous Jindong Formation, Hallyo Haesang National Park area, Goseong County, South Korea: Evidence and implications. Cretaceous Research 27: 70-101.

17 McCrea RT (2000) Vertebrate Palaeoichnology of the Lower Cretaceous (Lower Albian) Gates Formation of Alberta. M.Sc. Thesis, University of Saskatchewan, Saskatchewan , Canada. 204 p.

18 Casanovas ML, Ezquerra R, Fernández A, Pérez-Lorente F, Santaté JV, Torcida F (1993) Tracks a herd of webbed Ornithopoda and other footprint found in the same site (Igea, La Rioja, Spain). Revue de Paléobiologie 7: 29-36.

19 Alonso RN (1980) Icnitas de dinosaurios (Ornithopoda, Hadrosauridae) en el Cretácico Superior de norte de Argentina. Acta Geologica Lilloana: 15, 55-63.

20 Jaillard E, Cappetta J, Ellenberg P, Feist M, Grambast-Fessard N, Lefranc JP, Sigé B (1993) Sedimentology, paleontology, biostratigraphy and correlation of the Late Cretaceous Vilquechico Group of Southern Peru. Cretaceous Research 14: 623-661.

21 Vialov OS (1988) On the classification of dinosaurian traces. Ezhegodnik Vsesoyuznogo Paleontologicheskogo Obshchestva 31: 322-325.

22 Haubold H (1971) Ichnia Amphibiorum et Reptiliorum fossilium. Suttgart: Handbuch der Paläoherpetologie. 121 p.

23 Langston W (1960) A hadrosaurian ichnite. National Museum Canadian Natural History Paper 4: 1-9.

24 Lockley MG, Nadon G, Currie PJ (2003) A diverse dinosaur-bird footprint assemblages from the Lance Formation, Upper Cretaceous, Eastern Wyoming: implications for ichnotaxonomy. Ichnos 11: 229-249.

25 Xing LD, Harris JD, Dong ZM, Lin YL, Wei C, Guo SB, Ji Q (2009) Ornithopod (Dinosauria: Ornithischia) tracks from the Upper Cretaceous Zhutian Formation in the Nanxiong basin, Guangdong, China and general observations of large Chinese ornithopod footprints. Geological Bulletin of China 28: 829-843.

26 Zhen S, Li J, Han Z (1996) The study of dinosaur footprints in China. Sichuan Scientific and technological Publishing House, 110 p.

27 Sarjeant WAS, Delair JB, Lockley MG (1998) The footprints of *Iguanodon*: a history and taxonomic study. Ichnos 6: 183-202.

28 Meyer CA, Thüring B (2003) The first iguanodontid dinosaur tracks from the Swiss Alps (Schrattenkalk Formation, Aptian). Ichnos 10: 221-228.

29 Dong Z-M, Zhou Z-L, Wu S-Y (2003) Note on a hadrosaur footprint from Heilongjiang River Area of China. Vertebrata PalAsiatica 10: 324-326.

30 Llompart C, Casanovas ML, Santafé JV (1984) Un nuevo yacimiento de icnitas de dinosaurios en las facies garumnienses de la Conca de Tremp (Lleida, España). Acta Geológica Hispánica 19: 143-147.

31 Kuhn O (1958) Die fährten der vorzeitlichen Amphibien und reptilien. Verlagshaus Meisenbach. 64 p.

32 Leonardi G (1979) Nota preliminar sobre seis pistas de dinosaurios Ornithischia da Bacia do Rio do Peixe, em Souse, Paraiba, Brasil. Anais da Academia Brasileira de Ciências 51: 501-516.

33 Alonso RN, Marquillas RA (1986) Nueva localidad con huellas de dinosaurios y primer hallazgo de huellas de aves en la Formación Yacoraite (Maastrichtiense) del Norte Argentino. Actas 4º Congreso Argentino de Paleontología y Bioestratigrafía 2: 33-41.

34 Young CC (1960) Fossil footprints in China. Vertebrata PalAsiatica 4: 53-66.
